# Calcium Handling Remodeling Underlies Impaired Sympathetic Stress Response in Ventricular Myocardium from *Cacna1c* Haploinsufficient Rats

**DOI:** 10.3390/ijms24129795

**Published:** 2023-06-06

**Authors:** Hauke Fender, Kim Walter, Aytug K. Kiper, Jelena Plačkić, Theresa M. Kisko, Moria D. Braun, Rainer K. W. Schwarting, Susanne Rohrbach, Markus Wöhr, Niels Decher, Jens Kockskämper

**Affiliations:** 1 Institute of Pharmacology and Clinical Pharmacy, Faculty of Pharmacy, Biochemical and Pharmacological Center (BPC) Marburg, University of Marburg, 35032 Marburg, Germany; haukefender@gmx.de (H.F.); kimywalter@arcor.de (K.W.); jelenaplackic@yahoo.com (J.P.); 2 Institute of Physiology and Pathophysiology, Vegetative Physiology, University of Marburg, 35037 Marburg, Germany; aytug.kiper@staff.uni-marburg.de (A.K.K.); decher@staff.uni-marburg.de (N.D.); 3 Center for Mind, Brain and Behavior (CMBB), University of Marburg, 35032 Marburg, Germany; schwarti@staff.uni-marburg.de (R.K.W.S.); woehrm@staff.uni-marburg.de (M.W.); 4 Behavioral Neuroscience, Experimental and Biological Psychology, University of Marburg, 35032 Marburg, Germany; theresa.kisko@kuleuven.be (T.M.K.); moria.braun@gmx.de (M.D.B.); 5 Institute of Physiology, University of Gießen, 35392 Giessen, Germany; susanne.rohrbach@physiologie.med.uni-giessen.de; 6 Social and Affective Neuroscience Research Group, Laboratory of Biological Psychology, Research Unit Brain and Cognition, Faculty of Psychology and Educational Sciences, KU Leuven, B-3000 Leuven, Belgium; 7 Leuven Brain Institute, KU Leuven, B-3000 Leuven, Belgium

**Keywords:** CACNA1C, cardiomyocyte, calcium handling, remodeling

## Abstract

*CACNA1C* encodes the pore-forming α1C subunit of the L-type Ca^2+^ channel, Cav1.2. Mutations and polymorphisms of the gene are associated with neuropsychiatric and cardiac disease. Haploinsufficient *Cacna1c^+/−^* rats represent a recently developed model with a behavioral phenotype, but its cardiac phenotype is unknown. Here, we unraveled the cardiac phenotype of *Cacna1c^+/−^* rats with a main focus on cellular Ca^2+^ handling mechanisms. Under basal conditions, isolated ventricular *Cacna1c^+/−^* myocytes exhibited unaltered L-type Ca^2+^ current, Ca^2+^ transients (CaTs), sarcoplasmic reticulum (SR) Ca^2+^ load, fractional release, and sarcomere shortenings. However, immunoblotting of left ventricular (LV) tissue revealed reduced expression of Cav1.2, increased expression of SERCA2a and NCX, and augmented phosphorylation of RyR2 (at S2808) in *Cacna1c^+/−^* rats. The β-adrenergic agonist isoprenaline increased amplitude and accelerated decay of CaTs and sarcomere shortenings in both *Cacna1c^+/−^* and WT myocytes. However, the isoprenaline effect on CaT amplitude and fractional shortening (but not CaT decay) was impaired in *Cacna1c^+/−^* myocytes exhibiting both reduced potency and efficacy. Moreover, sarcolemmal Ca^2+^ influx and fractional SR Ca^2+^ release after treatment with isoprenaline were smaller in *Cacna1c^+/−^* than in WT myocytes. In Langendorff-perfused hearts, the isoprenaline-induced increase in RyR2 phosphorylation at S2808 and S2814 was attenuated in *Cacna1c^+/−^* compared to WT hearts. Despite unaltered CaTs and sarcomere shortenings, *Cacna1c^+/−^* myocytes display remodeling of Ca^2+^ handling proteins under basal conditions. Mimicking sympathetic stress with isoprenaline unmasks an impaired ability to stimulate Ca^2+^ influx, SR Ca^2+^ release, and CaTs caused, in part, by reduced phosphorylation reserve of RyR2 in *Cacna1c^+/−^* cardiomyocytes.

## 1. Introduction

The *CACNA1C* gene encodes the pore-forming α1C subunit of the L-type calcium (Ca^2+^) channel, Cav1.2, which is widely expressed in neurons, smooth muscle cells, and cardiac myocytes [1]. L-type Ca^2+^ channels (LTCCs) are macromolecular complexes consisting of the α1C subunit, which is the pore-forming subunit containing the voltage sensor, and auxiliary subunits (β2b, α2δ, γ), which modulate biophysical and trafficking properties of the cardiac Cav1.2 channel [2].

In neurons, Cav1.2 is primarily located on postsynaptic membranes, dendrites, and somata, where it controls activity-dependent Ca^2+^ entry and Ca^2+^-mediated regulation of transcription [3]. In cardiac myocytes, Cav1.2 is the primary source for Ca^2+^ entry upon depolarization during an action potential. This initial Ca^2+^ entry triggers the opening of cardiac ryanodine receptors (RyR2) located on the sarcoplasmic reticulum (SR), with subsequent SR Ca^2+^ release. Cytosolic Ca^2+^ binds to myofilaments and initiates contraction [4]. Afterward, cytosolic Ca^2+^ levels are decreased by Ca^2+^ re-uptake into the SR mediated by the sarcoplasmic/endoplasmic reticulum Ca^2+^ ATPase (SERCA2a), whose activity is regulated by the small inhibitory protein phospholamban (PLB), and by Ca^2+^ extrusion from the cell mediated by the sarcolemmal Na^+^/Ca^2+^ exchanger (NCX) [4]. During sympathetic activation, the above-mentioned Ca^2+^ handling proteins are modulated by β-adrenergic signaling, which results in the stimulation of protein kinase A (PKA) and Ca^2+^/calmodulin-dependent protein kinase II (CaMKII). PKA and CaMKII phosphorylate Cav1.2, RyR2, and PLB (among other proteins) to mediate the positive-inotropic and -lusitropic effects of sympathetic stimulation [4].

Due to its pivotal role in neurons and cardiac myocytes, *CACNA1C* has emerged as a major target of interest in neuropsychiatric and cardiovascular disease. In genome-wide association studies, single-nucleotide polymorphisms of the gene were associated with psychiatric disorders, such as major depression, bipolar disorder, and schizophrenia [1,3]. Therefore, the gene can be considered a common risk factor across many psychiatric disorders [5]. Furthermore, gain-of-function mutations in *CACNA1C* can lead to Timothy syndrome, a genetic disease comprising autism spectrum symptoms, arrhythmias and heart disease [6], Brugada syndrome, long QT syndrome, and early repolarization syndrome [2,7]. In cardiac myocytes, over-expression, but also down-regulation, of Cav1.2 can lead to hypertrophy and heart failure [8,9]. On the other hand, cardiac disease, such as hypertrophy, heart failure, or atrial fibrillation, may cause down-regulation of Cav1.2/L-type Ca^2+^ current in (atrial) cardiomyocytes [10,11,12]. Ca^2+^ channel blockers, such as verapamil, are inhibitors of Cav1.2/LTCCs, and they have been used for decades as anti-arrhythmic and anti-hypertensive drugs. A recent study involving patients with bipolar disorder, schizophrenia, and nonaffective psychosis has revealed that those patients taking LTCC blockers exhibited reduced rates (up to 70% reduction) of psychiatric hospitalizations and self-harm admissions [13]. Moreover, the use of verapamil was associated with reduced rates of depression in the Danish population [14]. Thus, there is ample evidence that altered expression and function of *CACNA1C*/Cav1.2 is involved in psychiatric and cardiac disease and that the two entities might be linked.

Haploinsufficient *Cacna1c^+/−^* rats represent a recently developed animal model for studying the role of Cav1.2 in psychiatric and cardiovascular disease [15]. Thus far, it has been used primarily to unravel the involvement of Cav1.2 in behavioral phenotypes. Results show that *Cacna1c^+/−^* rats exhibit deficits in pro-social behavior and communication together with other behavioral alterations consistent with autism-like symptoms [15,16,17], thus confirming a potential role of Cav1.2 in neuropsychiatric disorders. However, the cardiovascular phenotype of *Cacna1c^+/−^* rats has not been studied to date. Hence, we set out to characterize the cardiac phenotype of these animals with a focus on cardiomyocyte Ca^2+^ signaling. Our results reveal that, despite apparently normal L-type Ca^2+^ currents and Ca^2+^ transients, there is profound remodeling of Ca^2+^ handling proteins under baseline conditions, which underlies an impaired response to sympathetic stress.

## 2. Results

### 2.1. Cacna1c^+/−^ Rats Exhibit No Signs of Hypo- or Hypertrophy

**Figure 1:**
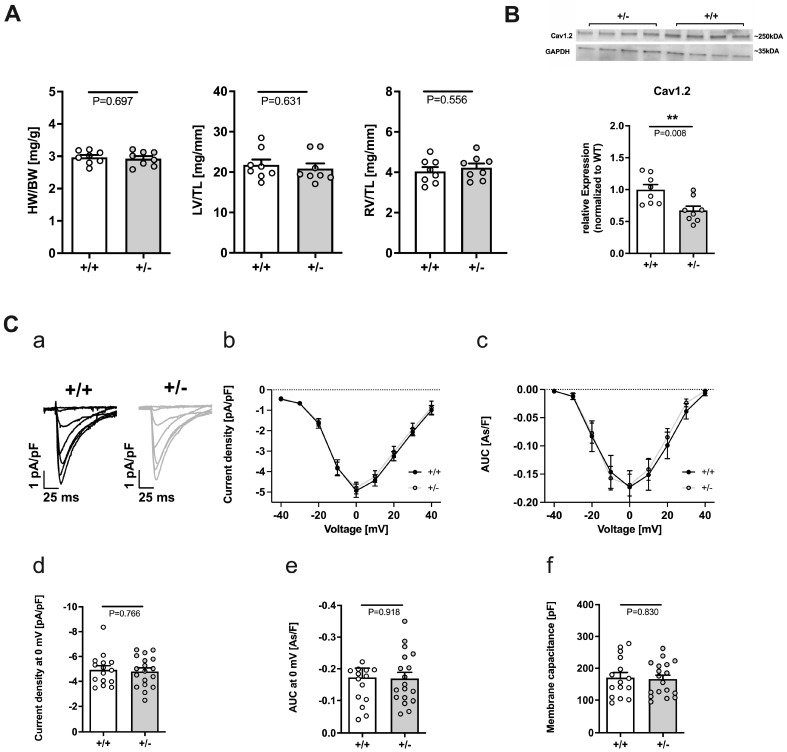
Characteristics of female *Cacna1c^+/−^* rats. (**A**) Heart weight to body weight ratio (HW/BW) and LV and RV weight normalized to tibia length (TL) did not differ between WT (+/+) and *Cacna1c^+/−^* (+/−) rats. Circles represent individual animals: N = 8 (WT); N = 8 (*Cacna1c^+/−^*); Student’s *t*-test, *p*-values as indicated. (**B**) Expression of L-type Ca^2+^ channels in *Cacna1c^+/−^* LV tissue. Western Blot analysis of expression of Cav1.2 (normalized to GAPDH) in *Cacna1c^+/−^* vs. WT (+/+) LV myocardium. Original immunoblot images show four LV samples from each genotype. Antibodies used are listed in Tables S1 and S2. First, the expression of Cav1.2 was normalized to the expression of GAPDH in each sample. Next, the Cav1.2/GAPDH ratios were averaged for the four WT samples on each Western Blot so that the mean value for WT amounts to 100%. Finally, the Cav1.2/GAPDH ratios of the four *Cacna1c^+/−^* samples were normalized to the averaged ratio of the four WT samples. Circles represent individual tissue samples. N = 8 for each genotype. Student’s *t*-test, *p*-values as indicated. In *Cacna1c^+/−^*, the expression of Cav1.2 was reduced. All original Western Blot images from this series are shown in Supplementary Material. (**C**) L-type Ca^2+^ current in ventricular myocytes. (**a**) Average current traces from WT (+/+) and *Cacna1c^+/−^* (+/−) ventricular myocytes during test voltages from −40 to +40 mV. (**b**) I_Ca_-V curves for WT and *Cacna1c^+/−^* myocytes. (**c**) Integrated L-type Ca^2+^ current (AUC, area under the curve) as a function of membrane voltage. (**d**) Maximal L-type Ca^2+^ current density at 0 mV. (**e**) Integrated L-type Ca^2+^ current at 0 mV. (**f**) Membrane capacitance. Circles represent individual myocytes. *n* = 15, N = 5 (WT); *n* = 18, N = 3 (*Cacna1c^+/−^*); Student’s *t*-test, *p*-values as indicated. No differences between genotypes were found with respect to L-type Ca^2+^ current, integrated L-type Ca^2+^ current, or membrane capacitance. **, *p* < 0.01.

For basal cardiac characterization of the animals, we investigated heart weight (Figure 1A). Neither the whole heart (normalized to body weight) nor left ventricular (LV) or right ventricular (RV) (normalized to tibia length) weight of *Cacna1c^+/−^* rats differed from WT (Figure 1A).

### 2.2. Calcium Handling in Cacna1c^+/−^ Ventricular Myocardium under Baseline Conditions

#### 2.2.1. Reduced Expression of Cav1.2 but Unaltered L-Type Ca^2+^ Current in *Cacna1c^+/−^*

First, we studied the expression of Cav1.2 in LV tissue from *Cacna1c^+/−^* rats. As shown in Figure 1B, the expression of Cav1.2 was reduced significantly by ≈30% in *Cacna1c^+/−^* rats. Second, we studied L-type Ca^2+^ current in isolated ventricular myocytes by means of whole-cell patch clamp. Ca^2+^ currents (I_Ca_) from WT and *Cacna1c^+/−^* myocytes obtained during the recording of a current-voltage (I-V) relationship were similar (Figure 1C(a)), and I_Ca_-V curves were nearly superimposable with peak current occurring at 0 mV (Figure 1C(b)). In addition, also the integrated L-type Ca^2+^ current at the various membrane voltages did not differ between the genotypes (Figure 1C(c)). Finally, there was no difference in maximal I_Ca_ (Figure 1C(d)), integrated I_Ca_ (Figure 1C(e)), or membrane capacitance, a measure of cell size, between WT and *Cacna1c^+/−^* rats (Figure 1C(f)).

Taken together, these results show that, in *Cacna1c^+/−^* rats under baseline conditions, Cav1.2 exhibits reduced expression, whereas both peak and integrated L-type Ca^2+^ current are unchanged.

#### 2.2.2. Electrically Stimulated Ca^2+^ Transients and Sarcomere Shortenings Are Unaltered in *Cacna1c^+/−^* Ventricular Myocytes

**Figure 2:**
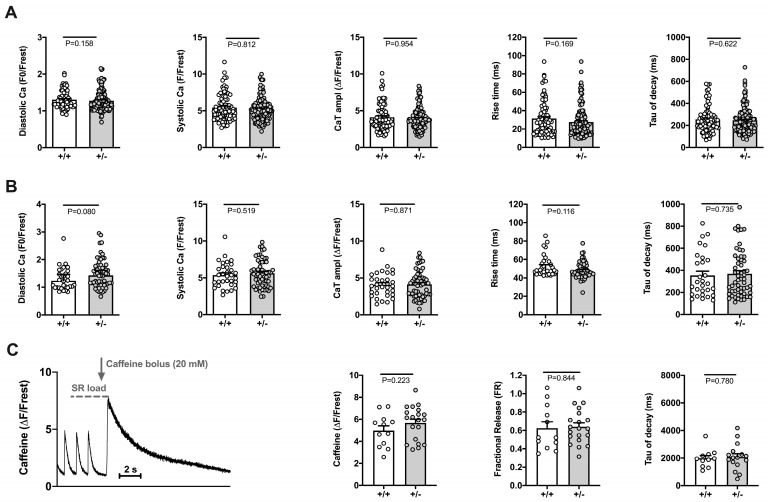
Characteristics of electrically stimulated CaTs, SR Ca^2+^ load, and fractional release in WT (+/+) and *Cacna1c^+/−^* (+/−) ventricular myocytes. Data from linescan imaging (**A**) and 2D confocal imaging (**B**) are shown. From left to right: diastolic Ca^2+^, systolic Ca^2+^, CaT amplitude, rise time, and tau of CaT decay. Circles represent individual myocytes: (**A**) *n* = 85, N = 8 (WT); *n* = 145, N = 14 (*Cacna1c^+/−^*); (**B**) *n* = 32, N = 3 (WT); *n* = 59, N = 6 (*Cacna1c^+/−^*); Mann–Whitney U-test, *p*-values as indicated. (**C**) Data from linescan imaging. Example trace of the caffeine protocol (left) used to estimate SR Ca^2+^ load and fractional release. Three electrically stimulated CaTs are followed by bolus application of caffeine (20 mM). Summarized data to the right: caffeine-induced CaT amplitude, fractional release, and tau of caffeine transient decay. Circles represent individual myocytes: *n* = 12, N = 4 (WT); *n* = 20, N = 3 (*Cacna1c^+/−^*); the number of cells is reduced for tau of decay (*n* = 11 WT; *n* = 16 *Cacna1c^+/−^*) because one of the following occurred: the recording ended before the decay was completed; the decay did not follow an exponential trend; the cell moved out of the field of view after caffeine application. Student’s *t*-test, *p*-values as indicated. (**A**–**C**) No differences between groups were found for any of the parameters studied.

**Figure 3:**
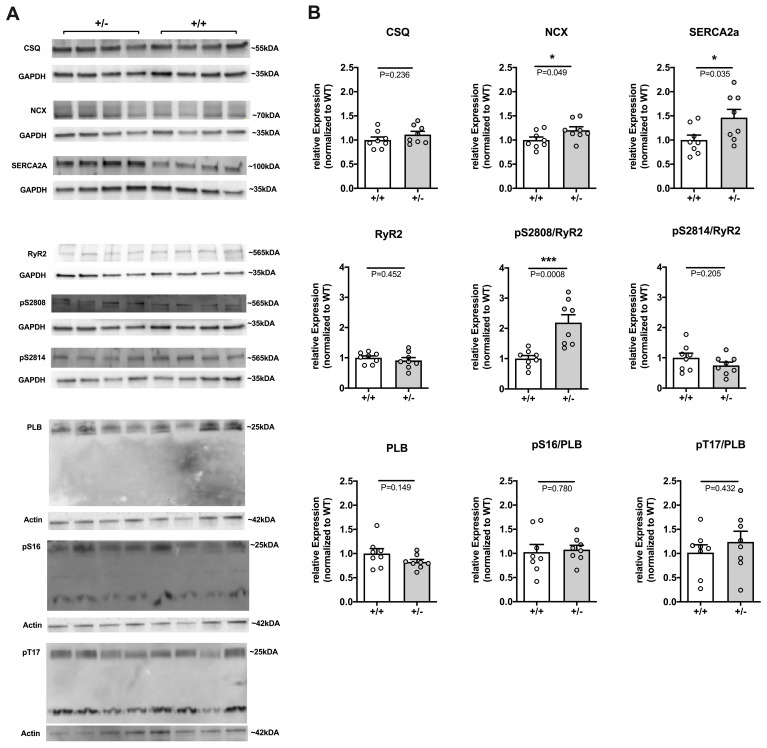
Expression and phosphorylation of major Ca^2+^ handling proteins in WT and *Cacna1c^+/−^* LV myocardium. (**A**) Original Western Blot images of four *Cacna1c^+/−^* (+/−) and 4 WT (+/+) samples. Proteins used for normalization (GAPDH, actin) and derived from the same membrane are shown below the protein of interest. Note that some of the loading controls are identical (see GAPDH control for CSQ and pS2808 and actin control for PLB and pS16), as the respective proteins were derived from the same membrane, and in case of PLB/pS16 stripping was performed. (**B**) Summarized data normalized to the expression of WT. Expression of CSQ, NCX, and SERCA2a (top row), expression of RyR2 and phosphorylation of RyR2 at S2808 (pS2808) and S2814 (pS2814) (middle), and expression of PLB and phosphorylation of PLB at S16 (pS16) and T17 (pT17) (bottom row). In *Cacna1c^+/−^*, expression of NCX and SERCA2a is up-regulated, and phosphorylation of RyR2 at S2808 is elevated. Circles represent individual animals: N = 8 (WT); N = 8 (*Cacna1c^+/−^*); Student’s *t*-test, *p*-values as indicated. Further information and all original Western Blot images from this series are shown in Supplementary Material. *, *p* < 0.05; *** *p* < 0.001.

**Figure 4:**
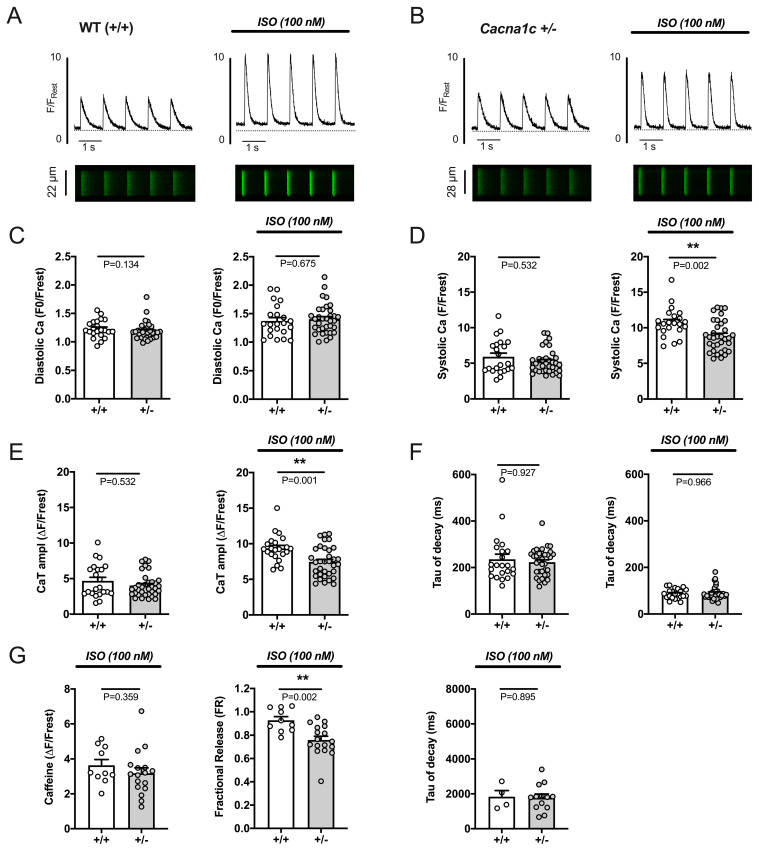
Effects of isoprenaline (ISO) on electrically stimulated CaTs, SR Ca^2+^ load, and fractional release in WT and *Cacna1c^+/−^* ventricular myocytes. Representative CaTs with their respective linescan images (edited as described in Supplementary Methods) of a WT (**A**) and *Cacna1c^+/−^* (**B**) ventricular myocyte before and during exposure to 100 nM ISO. ISO increased and accelerated CaTs in both cells. (**C**–**F**) Comparison of CaT characteristics between WT (+/+) and *Cacna1c^+/−^* (+/−) ventricular myocytes before (left panels) and during ISO exposure (right panels): (**C**) diastolic Ca^2+^, (**D**) systolic Ca^2+^, (**E**) CaT amplitude, (**F**) tau of CaT decay. In the presence of ISO, systolic Ca^2+^ and CaT amplitude were significantly lower in *Cacna1c^+/−^*. Circles represent individual cardiomyocytes: *n* = 22, N = 5 (WT); *n* = 34, N = 8 (*Cacna1c^+/−^*); Mann–Whitney U-test, *p*-values as indicated. (**G**) The amplitude of caffeine-induced CaTs (a measure for SR Ca^2+^ load), fractional SR Ca^2+^ release, and tau of decay of the caffeine-induced CaT in the presence of ISO. Fractional release in the presence of ISO is smaller in *Cacna1c^+/−^* myocytes, while SR Ca^2+^ load and tau of decay are comparable between genotypes. Circles represent individual cardiomyocytes: *n* = 10, N = 2 (WT); *n* = 17, N = 3 (*Cacna1c^+/−^*); the number of cells is reduced for tau of decay (*n* = 4 WT; *n* = 13 *Cacna1c^+/−^*), because one of the following occurred: the recording ended before the decay was completed; the decay did not follow an exponential trend; the cell moved out of the field of view after caffeine application. Student’s *t*-test, *p*-values as indicated. **, *p* < 0.01.

Electrically stimulated CaTs (1 Hz) were studied in isolated ventricular myocytes loaded with Fluo-4 either by means of linescan confocal imaging or by fast 2D confocal imaging. The results are shown in Figure 2A,B, respectively. Figure 3 shows expression of Ca^2+^-handling proteins. Examples of original linescan images and derived CaTs can be seen in Figure 4A,B (left panels), respectively. At 1 Hz stimulation, there were no differences in CaTs between WT and *Cacna1c^+/−^* rats. Diastolic and systolic Ca^2+^ levels, CaT amplitude and rise time and tau of decay of the CaT (from left to right) were all comparable between WT and *Cacna1c^+/−^* rats for both linescan (Figure 2A) and 2D imaging (Figure 2B) data. By means of caffeine bolus application (20 mM), we estimated SR Ca^2+^ load and fractional SR Ca^2+^ release in a subset of myocytes studied by linescan imaging (Figure 2C). Neither SR Ca^2+^ load nor fractional release differed significantly between WT and *Cacna1c^+/−^* myocytes. Moreover, tau of decay of the caffeine-induced CaT, a measure of NCX activity, was similar in WT and *Cacna1c^+/−^* myocytes.

CaTs underlie contraction. Hence, we also studied the contractile activity of ventricular myocytes by means of sarcomere shortening. The protocol was identical to the one used for imaging of CaTs, i.e., 1 Hz electrical stimulation, except the myocytes were not loaded with any Ca^2+^ indicator. Supplementary Figure S1A,B illustrate original sarcomere shortenings of WT and *Cacna1c^+/−^* myocytes under baseline conditions (left panels), and average results are shown in Supplementary Figure S1C (left panels). In line with the CaT results, sarcomere shortenings showed similar characteristics in WT and *Cacna1c^+/−^* myocytes. Diastolic sarcomere length (SL), fractional shortening, time-to-peak 90%, and relaxation time 90% were all comparable between WT and *Cacna1c^+/−^* myocytes.

Thus, under baseline conditions, *Cacna1c^+/−^* myocytes exhibit Ca^2+^ handling (CaTs, SR Ca^2+^ load, and fractional release) and contractions very similar to WT myocytes.

#### 2.2.3. Remodeling of Major Ca^2+^ Handling Proteins in *Cacna1c^+/−^* LV Myocardium

Next, we performed a Western Blot analysis of LV tissue to investigate expression levels of the most important Ca^2+^ handling proteins and their major phosphorylation sites in WT and *Cacna1c^+/−^* rats. Figure 3A shows original Western Blots whereas Figure 3B summarizes results for expression of CSQ, NCX, SERCA2a, RyR2, and PLB and for phosphorylation of RyR2 at S2808 and S2814 and of PLB at S16 and T17. Expression of RyR2, CSQ, and PLB remained unchanged between WT and *Cacna1c^+/−^* rats. However, *Cacna1c^+/−^* rats developed a moderate but significant up-regulation of both major Ca^2+^ extrusion proteins, i.e., NCX (by ≈20%) and SERCA2a (by ≈50%). Furthermore, the phosphorylation of PLB at both S16 and T17 was unaltered. By contrast, RyR2 exhibited altered phosphorylation. While phosphorylation at S2814 did not differ, there was a highly significant up-regulation of phosphorylation at S2808 by ≈120% in *Cacna1c^+/−^* rats.

In summary, despite unaltered L-type Ca^2+^ current (Figure 1), CaTs (Figure 2), and contraction (Supplementary Figure S1), *Cacna1c^+/−^* exhibit remodeling of Ca^2+^ handling proteins under baseline conditions, consisting of reduced expression of Cav1.2 (Figure 1), increased expression of NCX and SERCA2a (Figure 3), and augmented phosphorylation of RyR2 at S2808 (Figure 3), a site predominantly phosphorylated by PKA.

### 2.3. Calcium Handling in Cacna1c^+/−^ Ventricular Myocardium during Sympathetic Stress

#### 2.3.1. Attenuated Response of Ca^2+^ Transients and Sarcomere Shortenings to Stimulation by ISO in *Cacna1c^+/−^* Ventricular Myocytes

*Cacna1c^+/−^* rats exhibited altered PKA-dependent phosphorylation of RyR2 in the ventricular myocardium under baseline conditions. Furthermore, previous studies revealed deficits in the pro-social behavior of *Cacna1c^+/−^* rats [15,16,17] and, in the mouse model, behavior suggestive of heightened anxiety levels and altered stress response [18]. Therefore, we studied the response of WT and *Cacna1c^+/−^* ventricular myocardium and myocytes to sympathetic stress. Sympathetic stress was mimicked by exposure to ISO (β-adrenergic agonist) and/or an increase in stimulation frequency.

CaTs were measured in ventricular myocytes electrically stimulated at 1 Hz and exposed to ISO using linescan imaging. A high ISO concentration of 100 nM was used in these experiments to obtain a near-maximal effect. The results are shown in Figure 4. Figure 4A,B illustrate original linescan images and derived CaTs from WT and *Cacna1c^+/−^* myocytes before (left panels) and during (right panels) stimulation with ISO. In both groups, ISO greatly increased systolic Ca^2+^ and CaT amplitude and accelerated CaT decay. In the presence of ISO, diastolic Ca^2+^ did not differ between WT and *Cacna1c^+/−^* myocytes (Figure 4C). However, during β-adrenergic stimulation, both systolic Ca^2+^ (Figure 4D) and CaT amplitude (Figure 4E) were significantly smaller in *Cacna1c^+/−^* compared to WT myocytes. With respect to CaT decay kinetics, the ISO-mediated reduction in tau of decay was similarly pronounced in both genotypes (Figure 4F).

We also studied the frequency dependence of CaTs and the ISO effect (Supplementary Figure S2). Under baseline conditions (Supplementary Figure S2A), an increase in stimulation frequency from 1 Hz to 2 Hz and 4 Hz (mimicking the positive-chronotropic effect of sympathetic stress) caused increases in diastolic Ca^2+^ and acceleration of CaT decay that were similarly pronounced in WT and *Cacna1c^+/−^* myocytes. At 4 Hz, CaT amplitude was larger in *Cacna1c^+/−^* than in WT myocytes. In the presence of ISO (Supplementary Figure S2B), the frequency-dependent increase in diastolic Ca^2+^ persisted, and systolic Ca^2+^ and CaT amplitude showed almost no alterations with increasing frequencies in both genotypes. Importantly, the attenuated ISO effect on systolic Ca^2+^ and CaT amplitude in *Cacna1c^+/−^* myocytes was also observed at higher frequencies. By contrast, the ISO-mediated acceleration of CaT decay was essentially identical in both genotypes at all frequencies studied.

SR Ca^2+^ regulation in the presence of ISO was studied in WT and *Cacna1c^+/−^* ventricular myocytes stimulated at 1 Hz (Figure 4G). Under these conditions, SR Ca^2+^ load, i.e., the amplitude of the caffeine transient, was similar in WT and *Cacna1c^+/−^* myocytes. In both genotypes, the fractional release was augmented upon ISO treatment (compare Figure 2C); however, this effect was less pronounced in the *Cacna1c^+/−^* group resulting in a significantly smaller fractional release (≈76%) compared to WT (≈92%) myocytes. Decay of the caffeine-induced CaT, again, was comparable between WT and *Cacna1c^+/−^* myocytes and similar to baseline conditions (compare Figure 2C).

Finally, we measured sarcomere shortenings of WT and *Cacna1c^+/−^* ventricular myocytes stimulated at 1 Hz during β-adrenergic stimulation with 100 nM ISO (Supplementary Figure S1). In line with the results of CaTs (Figure 4), we found that ISO greatly increased shortening amplitude (fractional shortening) and accelerated relaxation (re-lengthening) in both genotypes, while under baseline conditions, there were no major differences between WT and *Cacna1c^+/−^* myocytes; in the presence of ISO, *Cacna1c^+/−^* myocytes exhibited larger diastolic and systolic sarcomere length and reduced fractional shortening. Relaxation time, however, did not show any differences between both genotypes.

Taken together, these results show that β-adrenergic stimulation using a high concentration of ISO (100 nM) causes a large, Ca^2+^-dependent, positive-inotropic and positive-lusitropic effect in both WT and *Cacna1c^+/−^* ventricular myocytes. However, the positive-inotropic effect is attenuated in *Cacna1c^+/−^* myocytes because of an attenuated increase in CaTs caused by reduced fractional SR Ca^2+^ release at similar SR Ca^2+^ load in the presence of ISO. The positive-lusitropic effect by ISO, on the other hand, is unaltered in *Cacna1c^+/−^* myocytes.

#### 2.3.2. Reduced Potency of ISO to Stimulate Ca^2+^ Transients and Sarcomere Shortenings in *Cacna1c^+/−^* Ventricular Myocytes

**Figure 5:**
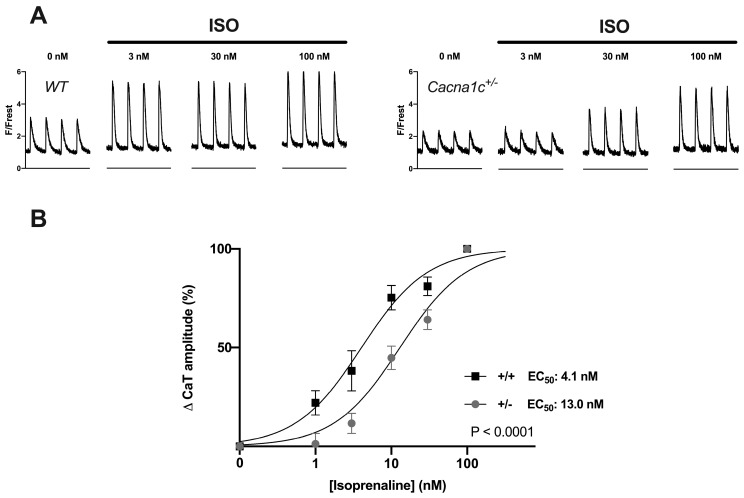
Concentration dependence of isoprenaline (ISO) effect on CaT amplitude in WT and *Cacna1c^+/−^* ventricular myocytes. (**A**) Example CaT traces from one WT (**left**) and one *Cacna1c^+/−^* (**right**) ventricular myocyte treated with increasing concentrations of ISO (as indicated). (**B**) Resulting concentration-response curves for WT (+/+, squares) and *Cacna1c^+/−^* (+/−, circles) ventricular myocytes. Values for CaT amplitude were rundown-corrected and normalized (0% = CaT amplitude before ISO; 100% = CaT amplitude at 100 nM ISO) as detailed in Supplementary Methods. Number of cells/animals (WT/*Cacna1c^+/−^*): 0 nM: *n* = 52/55, N = 10/10, 1 nM: *n* = 27/17, N = 5/4, 3 nM: *n* = 17/26, N = 4/6, 10 nM: *n* = 22/24, N = 5/6, 30 nM: *n* = 21/31, N = 5/6, 100 nM: *n* = 52/55, N = 10/10. In *Cacna1c^+/−^* myocytes, the concentration-response curve was shifted to the right. EC_50_ values: 4.1 nM (WT, +/+) and 13.0 nM (*Cacna1c^+/−^*, +/−).

Using linescan imaging, we investigated the concentration dependence of the ISO-induced increase in CaTs in WT and *Cacna1c^+/−^* myocytes stimulated at 1 Hz. Ventricular myocytes from both genotypes were treated with progressively increasing ISO concentrations ranging from 1 to 100 nM. Following rundown correction, the ISO response on CaT amplitude was normalized to the response at 100 nM ISO (see Supplementary Methods). Figure 5 shows the results. Original CaTs from WT and *Cacna1c^+/−^* myocytes are illustrated in Figure 5A, and the corresponding concentration-response curves are shown in Figure 5B. There was a significant rightward shift of the concentration-response curve in *Cacna1c^+/−^* myocytes (EC_50_ = 13.0 nM) as compared to WT (EC_50_ = 4.1 nM).

In similar experiments, we determined the concentration dependence of the ISO-induced increase in fractional shortening in WT and *Cacna1c^+/−^* myocytes stimulated at 1 Hz. The results are shown in Supplementary Figure S3. Here, we also observed a rightward shift in the concentration-response curve of *Cacna1c^+/−^* myocytes (EC_50_ = 6.1 nM) as compared to WT (EC_50_ = 3.9 nM).

Thus, ISO exhibits a reduced potency to stimulate CaT amplitude and fractional shortening in *Cacna1c^+/−^* compared to WT myocytes.

#### 2.3.3. Reduced Efficacy of ISO to Increase RyR2 Phosphorylation in *Cacna1c^+/−^* LV Myocardium

**Figure 6:**
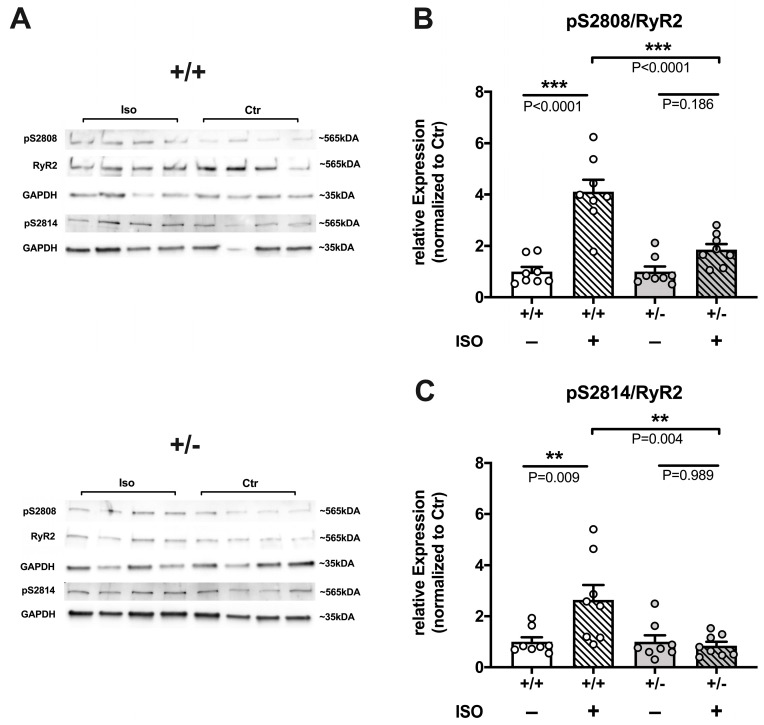
Phosphorylation of RyR2 in WT (+/+) and *Cacna1c^+/−^* (+/−) LV myocardium treated with 100 nM isoprenaline (ISO). Untreated LV myocardium served as control (Ctr). Phosphorylation of RyR2 at S2808 and at S2814. Original immunoblot images (**A**) and summarized data (**B**,**C**) is shown. Phosphorylation increases of RyR2 in ISO-treated hearts were stronger at (**B**) S2808 and (**C**) S2814 in WT compared to *Cacna1c^+/−^*. One representative Western Blot is presented for each protein/phosphorylation site and genotype with four Ctrl and four ISO-treated samples. GAPDH was used for normalization. For immunoblot images in (**A**), i.e., RyR2 and pS2808, GAPDH is the same for both proteins in each genotype because stripping was performed. Data is normalized to the average value of Ctr (=100%). Circles represent individual animals: N = 8 for each genotype (WT, *Cacna1c^+/−^*) and treatment (Ctrl vs. ISO); one-way ANOVA followed by Tukey’s post hoc test, *p*-values as indicated. All original Western Blot images from this series are shown in Supplementary Material. **, *p* < 0.01; ***, *p* < 0.001.

In order to examine the effect of sympathetic stress on the phosphorylation of Ca^2+^ handling proteins, Langendorff-perfused hearts were treated with ISO (100 nM), and LV tissue homogenates of these hearts were probed for phosphorylation of RyR2 and PLB at PKA and CaMKII sites by means of Western blotting. The results are shown in Figure 6 and Supplementary Figure S4. With regard to RyR2 (Figure 6), ISO increased RyR2 phosphorylation by ≈310% at S2808 in LV myocardium from WT rats. By contrast, the ISO effect was greatly attenuated in LV myocardium from *Cacna1c^+/−^* rats, where phosphorylation at S2808 was not increased in a statistically significant manner (Figure 6B). Similarly, phosphorylation of RyR2 at S2814 was increased by ISO by ≈160% in WT LV myocardium, whereas it was not changed at all in LV myocardium from *Cacna1c^+/−^* rats (Figure 6C).

On the other hand, ISO greatly increased the phosphorylation of PLB in both WT and *Cacna1c^+/−^* LV tissue (Supplementary Figure S4A). The effect was similar in both genotypes, with increases in PLB phosphorylation of ≈540% and ≈330% at S16 and T17 in *Cacna1c^+/−^* and ≈980% and ≈450% at S16 and T17 in WT LV myocardium (Supplementary Figure S4B).

Thus, a high concentration of ISO increased phosphorylation of PLB at PKA and CaMKII sites in both WT and *Cacna1c^+/−^* LV myocardium by a similar extent, but the effect on RyR2 phosphorylation differed with the phosphorylation increase at S2808 and S2814 being much larger in WT as compared to *Cacna1c^+/−^* LV myocardium.

#### 2.3.4. Reduced Efficacy of ISO to Increase Sarcolemmal Ca^2+^ Influx in *Cacna1c^+/−^* Ventricular Myocytes

**Figure 7:**
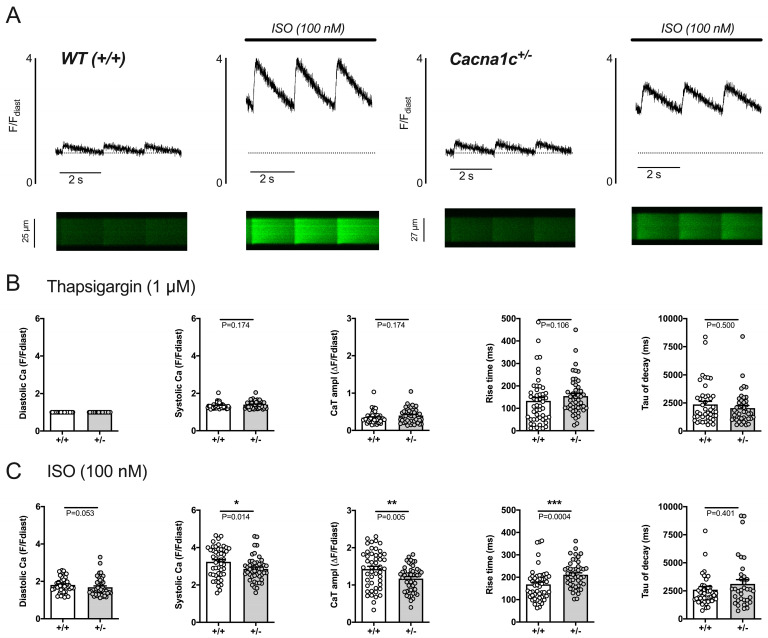
Sarcolemmal Ca^2+^ influx in WT (+/+) and *Cacna1c^+/−^* (+/−) ventricular myocytes before and during exposure to 100 nM isoprenaline (ISO). (**A**) Representative CaTs with their respective linescan images (edited as described in Supplementary Methods) of thapsigargin-treated myocytes of WT (left) and *Cacna1c^+/−^* (right) before and during exposure to 100 nM ISO. (**B**) CaT of *Cacna1c^+/−^* and WT myocytes did not differ after treatment with thapsigargin. SR depletion and inhibition of SERCA are evident from the small CaT amplitude and slow tau of decay. (**C**) ISO leads to a significantly greater increase in CaT amplitude and systolic Ca in WT myocytes. Circles represent individual cardiomyocytes: (**B**): *n* = 49/47, N = 5/5 (WT/*Cacna1c^+/−^*); tau of decay: *n* = 44/42 (WT/Cacna1c^+/−^). (**C**): *n* = 49/47, N = 5/5 (WT/*Cacna1c^+/−^*); tau of decay: *n* = 47/35 (WT/*Cacna1c^+/−^*). Mann–Whitney U test, *p*-values as indicated. Cell counts for tau of decay are reduced because, in some cases, the decline did not follow an exponential trend. *, *p* < 0.05; **, *p* < 0.01; ***, *p* < 0.001.

Thus far, results have shown an impaired ability (potency and efficacy) of ISO to increase CaTs and contractions in *Cacna1c^+/−^* ventricular myocytes that could be explained, in part, by impaired ISO-induced phosphorylation of RyR2 and attenuated ISO-induced increase in fractional SR Ca^2+^ release. In order to examine the effects of ISO on sarcolemmal Ca^2+^ influx, which is mainly carried by Ca^2+^ influx via L-type Ca^2+^ channels and which represents the major trigger for SR Ca^2+^ release via RyR2, we imaged sarcolemmal Ca^2+^ influx in intact ventricular myocytes with SR function blocked (see details in Supplementary Methods) before and during exposure to 100 nM ISO. Figure 7A illustrates original linescan images and corresponding CaT traces for a WT (left) and a *Cacna1c^+/−^* (right) myocyte before and during ISO exposure. Baseline CaTs were very small in amplitude, and CaT decay was slow in both cells because SERCA function was blocked, the SR was depleted of Ca^2+^, and sarcolemmal Ca^2+^ influx contributes only a minor fraction (≈7%) to the cytosolic CaT (with intact SR function) in rat ventricular myocytes [19]. Application of ISO increased both diastolic and systolic Ca^2+^ in both cells, but the increase appeared to be less pronounced in the *Cacna1c^+/−^* myocyte. Figure 7B,C shows average results for CaTs obtained under these conditions before (Figure 7B, thapsigargin) and during ISO exposure (Figure 7C, ISO). At baseline (Figure 7B), CaTs elicited by sarcolemmal Ca^2+^ influx did not differ between WT (+/+) and *Cacna1c^+/−^* (+/−) myocytes, in line with our observation of identical L-type Ca^2+^ currents (see Figure 1C). Following ISO stimulation, sarcolemmal Ca^2+^ influx was greatly increased in both genotypes, but the increase in both systolic Ca^2+^ and CaT amplitude was significantly smaller in *Cacna1c^+/−^* myocytes, suggesting impaired ISO-dependent stimulation of sarcolemmal Ca^2+^ influx carried by L-type Ca^2+^ channels in *Cacna1c^+/−^* compared to WT myocytes.

## 3. Discussion

*Cacna1c* haploinsufficient rats represent a unique animal model with a global heterozygous knockout of the gene encoding the Cav1.2 channel, found in neurons, cardiomyocytes, and other cell types. Previous studies have established a behavioral phenotype of the animals [15,16,17]. Here, we demonstrate a cardiac phenotype with regard to myocyte Ca^2+^ handling, which is unmasked during sympathetic stress-induced changes in RyR2-mediated SR Ca^2+^ release. The results imply that alterations in *CACNA1C* per se can predispose to alterations in both behavior and cardiac function, providing a potential molecular link between neuropsychiatric disorders and cardiac disease.

### 3.1. Cardiomyocyte Function under Basal Conditions Appears Unaltered in Cacna1c^+/−^ Rats

Despite reduced expression of Cav1.2 (by ≈30%), cardiomyocyte function of *Cacna1c^+/−^* rats appeared unaltered under basal conditions. L-type Ca^2+^ current, sarcolemmal Ca^2+^ influx, electrically stimulated CaTs, SR Ca^2+^ load and release, and sarcomere shortenings were all very similar in *Cacna1c^+/−^* and WT myocytes. Moreover, ventricular myocyte size (membrane capacitance) was also very similar, as was heart (and LV and RV) size, suggesting no gross structural and functional alterations of the heart and its cardiomyocytes. Therefore, it appears that *Cacna1c^+/−^* rats were able to compensate for reduced expression of Cav1.2 to maintain cardiomyocyte and heart function under basal conditions.

This is consistent with the findings of a mouse model with an inducible knockout of one Cav1.2 allele [20]. Another study conducted with a heterozygous α1C knockout mouse model observed the development of mild cardiac hypertrophy by 32 weeks of age [9]. The reduced (≈30%) cardiac protein levels of Cav1.2/α1C, together with the essentially unaltered L-type Ca^2+^ current observed here, are in line with findings of the mouse model with an inducible knockout of one Cav1.2 allele [20], in which a ≈20% reduction in Cav1.2 protein expression and unaltered L-type Ca^2+^ current were found. However, Goonasekera et al. [9], observed ≈25% less L-type Ca^2+^ current in their haploinsufficient mouse model, along with reduced SR Ca^2+^ load, CaTs, and contractions.

How is it possible that *Cacna1c^+/−^* myocytes exhibit reduced expression of Cav1.2 but unaltered L-type Ca^2+^ current? Our present results do not provide an answer to this question, and we can only speculate on the underlying cellular mechanisms. Cav1.2 expression, location, and function are regulated in a complex fashion by auxiliary subunits, regulatory proteins (such as RAD), phosphorylation events (e.g., by PKA), membrane trafficking, turnover or degradation of the channel, e.g., [2,21,22,23]. Potentially, alterations in some of these regulation processes may have led to an unaltered membrane density of Cav1.2 in *Cacna1c^+/−^* myocytes despite reduced global expression. Alternatively, Cav1.2 channels in *Cacna1c^+/−^* myocytes may exhibit increased single-channel conductance or open probability. This latter scenario seems unlikely, however, as it would result in a leftward shift of the I_Ca_-V curve, which we did not observe.

### 3.2. Remodeling of Ca^2+^ Handling Proteins under Basal Conditions in Cacna1c^+/−^ LV Myocardium

Despite unaltered cardiomyocyte Ca^2+^ handling (L-type Ca^2+^ currents, sarcolemmal Ca^2+^ influx, CaTs, and SR Ca^2+^ load and release) under basal conditions, LV myocardium from *Cacna1c^+/−^* rats exhibited profound alterations in the expression and phosphorylation of major Ca^2+^ handling proteins. These alterations consisted of reduced expression (by ≈30%) of Cav1.2, increased expression of Ca^2+^ removal proteins NCX (by ≈20%) and SERCA2a (by ≈50%), and increased phosphorylation of RyR2 at S2808 (by ≈120%). This remodeling of Ca^2+^ handling proteins may be the reason why *Cacna1c^+/−^* myocytes were able to maintain normal CaTs under basal conditions. RyR2 exhibited larger phosphorylation at S2808, a site predominantly phosphorylated by PKA, in *Cacna1c^+/−^* myocytes. By contrast, phosphorylation of PLB at either S16 or T17 was unaltered. Thus, only proteins in the junctional cleft, i.e., RyR2, exhibited higher PKA-dependent phosphorylation suggesting subcellularly restricted regulation of protein activity in *Cacna1c^+/−^* myocytes possibly to promote Ca^2+^-induced SR Ca^2+^ release in order to compensate for the reduced Cav1.2 protein levels.

### 3.3. Sympathetic Stress Unmasks a Defective Ca^2+^ Handling Phenotype in Cacna1c^+/−^ Ventricular Myocytes

Under basal conditions, *Cacna1c^+/−^* myocytes were able to maintain normal CaTs and contractions. Mimicking sympathetic stress by applying the β-adrenergic receptor agonist isoprenaline, however, unmasked a Ca^2+^ handling phenotype in *Cacna1c^+/−^* myocytes. Following β-adrenergic receptor activation by isoprenaline, rising cAMP levels and PKA activity lead to positive-inotropic and -lusitropic effects through phosphorylation of Ca^2+^ handling proteins. Isoprenaline exhibited both lower potency and efficacy in increasing CaTs and sarcomere shortenings in *Cacna1c^+/−^* myocytes. Thus, the isoprenaline-induced positive-inotropic effect was attenuated in *Cacna1c^+/−^* myocytes, and this could be explained by a smaller increase in sarcolemmal Ca^2+^ influx and fractional SR Ca^2+^ release resulting in a smaller increase in CaTs and hence contractions. Sarcolemmal Ca^2+^ influx is largely carried by L-type Ca^2+^ channels and acts as a trigger for SR Ca^2+^ release. Sarcolemmal Ca^2+^ influx was identical between WT and *Cacna1c^+/−^* myocytes under baseline conditions, but the isoprenaline-mediated increase in sarcolemmal Ca^2+^ influx was attenuated in *Cacna1c^+/−^* myocytes suggesting impaired β-adrenergic regulation of L-type Ca^2+^ channels in *Cacna1c^+/−^* myocytes. This also means reduced Ca^2+^-induced SR Ca^2+^ release in *Cacna1c^+/−^* myocytes. SR Ca^2+^ release is mediated by RyR2, and RyR2 activity, in turn, is regulated by SR Ca^2+^ load (intraluminal Ca^2+^) and RyR2 phosphorylation (among other regulators) [24,25]. SR Ca^2+^ load in the presence of isoprenaline was similar in *Cacna1c^+/−^* and WT myocytes and is thus unlikely to account for the observed difference in a fractional release. As the PKA-dependent phosphorylation of RyR2 at S2808 was elevated already under basal conditions in *Cacna1c^+/−^* myocytes, we suggest that a smaller phosphorylation reserve was accountable for the attenuated response to isoprenaline. In line with this hypothesis, experiments with Langendorff-perfused hearts directly showed that, in *Cacna1c^+/−^* LV myocardium, isoprenaline caused no significant increase in RyR2 phosphorylation at S2808, but a highly significant increase in WT LV myocardium (by ≈310%), matching our cellular findings. Similarly, isoprenaline elicited increased RyR2 phosphorylation at S2814 in WT (by ≈160%) but not *Cacna1c^+/−^* LV myocardium.

In contrast to the positive-inotropic effect, the positive-lusitropic effect elicited by isoprenaline did not exhibit any differences between *Cacna1c^+/−^* and WT myocytes. In both genotypes, isoprenaline caused a comparable acceleration of CaT decay and relaxation time. The main actor for CaT decay and relaxation is the SERCA pump, whose activity is regulated by PLB phosphorylation [26]. PLB phosphorylation at S16 (by PKA) and at T17 (by CaMKII) was comparable under basal conditions, and it was increased to a similar extent at both sites by isoprenaline treatment, matching the functional cellular data. This observation is important for two reasons: (1) It shows that β-adrenergic stimulation per se is not impaired in *Cacna1c^+/−^* hearts suggesting a full-blown increase in cAMP levels and PKA and CaMKII activities with subsequent phosphorylation of certain target proteins following stimulation with isoprenaline. (2) It implies that there are compartmentalized alterations of PKA- and CaMKII-dependent phosphorylation in the junctional cleft and the microenvironment of the RyR2 in *Cacna1c^+/−^* myocytes following stimulation with isoprenaline. The latter notion is in line with previous studies demonstrating local regulation of RyR2 activity by enzymes (e.g., kinases, phosphatases, or phosphodiesterases) in its microenvironment, e.g., [27,28].

### 3.4. CACNA1C Gene as a Mechanistic Link between Psychiatric Disorders and Cardiac Disease?

Clinically, there are mutual associations between neuropsychiatric disorders and cardiac diseases. For example, depression or anxiety are associated with several cardiac diseases, including coronary artery disease, myocardial infarction, heart failure, and atrial fibrillation [29,30,31,32,33]. Polymorphisms and mutations in the *CACNA1C* gene are associated with both psychiatric and cardiac disease [3,5,6]. In both neurons and cardiomyocytes, Cav1.2 exerts pivotal functions, including Ca^2+^-dependent regulation of transcription and excitation-contraction coupling. Therefore, alterations in expression and function of Cav1.2 are likely to impair both neuronal and cardiac function, suggesting that Cav1.2 may be a mechanistic link between psychiatric disorders and cardiac disease.

Here, we provide further evidence for this notion using a unique rat model, i.e., *Cacna1c* haploinsufficient rats. Previous studies have unraveled a behavioral phenotype of this animal model, including deficits in pro-social behavior and communication together with other behavioral alterations consistent with autism-like symptoms [15,16,17]. This includes the reduced emission of affiliative calls during rough-and-tumble play and weaker responses to such important socio-affective signals. Our study extends these findings and shows that *Cacna1c^+/−^* rats also exhibit a cardiac phenotype. Hence, reduced expression of Cav1.2 appears to be sufficient to induce both a behavioral and a cardiac phenotype. The cardiac phenotype occurred in the absence of gross structural alterations as both ventricular myocytes and whole hearts (LV and RV) of *Cacna1c^+/−^* rats were similar in size compared to WT.

Despite the apparently normal function of cardiomyocytes under basal conditions, remodeling of Ca^2+^ handling proteins was observed in *Cacna1c^+/−^* hearts, suggesting that alterations in Cav1.2 expression are sufficient to induce remodeling. Some features of this Ca^2+^ handling remodeling in *Cacna1c^+/−^* myocytes are reminiscent of the remodeling observed in other cardiac diseases. In particular, increased expression/activity of NCX and elevated PKA-dependent phosphorylation of RyR2 are hallmarks of heart failure [24,34,35,36]. In hypertensive heart disease, an increase in NCX expression/activity and an increase in RyR2 phosphorylation at S2808 occurs late during the disease course [37,38]. Both increased NCX expression/activity and elevated RyR2 phosphorylation has been implicated in an increased propensity for arrhythmias [35,39]. Moreover, a blunted positive-inotropic response to β-adrenergic stimulation, as seen in *Cacna1c^+/−^* myocytes, is also found in hypertrophy and heart failure [35,40,41,42], although the underlying cellular mechanisms may differ. However, other features of the *Cacna1c^+/−^* model clearly differ from the Ca^2+^ handling remodeling in hypertrophy and heart failure, such as the increased expression of SERCA (decreased in heart failure [43]) or the lack of altered phosphorylation of RyR2 at S2814 (increased in heart failure [44,45]). Increased phosphorylation of RyR2 at S2814 (by CaMKII) in heart failure appears to be the dominant mechanism rendering RyR2 leaky with detrimental effects on contractility and arrhythmogenesis [45,46,47,48]. Thus, the Ca^2+^ handling remodeling phenotype observed in *Cacna1c^+/−^* hearts appears to be unique and includes both potentially adverse and protective features.

### 3.5. Limitations

Action potential duration and shape are important determinants of sarcolemmal Ca^2+^ influx and the CaT. In addition, CaMKII and PKA have various targets other than Ca^2+^-regulating proteins. Notably, these targets include sarcolemmal ion channels, such as voltage-dependent Na^+^ and K^+^ channels [49,50,51]. Altered regulation of these ion channels, in turn, may alter action potential duration and shape and Ca^2+^ regulation [51,52]. We did not address these issues in our present investigation, but future studies on Cacna1c^+/−^ myocytes should certainly aim at characterizing a potential electrophysiological phenotype in this model.

### 3.6. Conclusions

Under baseline conditions, *Cacna1c^+/−^* myocytes are able to maintain normal CaTs and contractions. There is remodeling of Ca^2+^ handling proteins though, consisting of increased expression of NCX and SERCA and elevated phosphorylation of RyR2 at S2808. *Cacna1c^+/−^* myocytes display an attenuated positive-inotropic—but not positive-lusitropic—response to isoprenaline stimulation mediated by an impaired ability to increase sarcolemmal Ca^2+^ influx and SR Ca^2+^ release. This attenuated β-adrenergic response is caused by a reduced phosphorylation reserve of RyR2 at S2808 and S2814. This Ca^2+^ handling remodeling in *Cacna1c^+/−^* rats under basal conditions may alter the heart’s response to stressors and thus cause an increased susceptibility to cardiac disease under conditions of mechanical or mental stress. Since *CACNA1C* is a prominent risk factor for many psychiatric disorders, which often go along with heightened stress or anxiety levels, it is interesting to see that a heterozygous knockout of the gene in rats also leads to an altered reaction to stress at the level of the heart. Thus, alterations in *CACNA1C*/Cav1.2 may constitute a molecular link between neuropsychiatric disorders and cardiac disease.

## 4. Materials and Methods

A detailed description of methods can be found in the Supplementary Material.

### 4.1. Animals

The study was approved by local animal welfare authorities and was performed in accordance with the European Union Council Directive 2010/63/EU and the German Animal Welfare Act. *Cacna1c^+/−^* rats were generated by means of zinc finger technology by SAGE Labs (now Horizon Discovery Ltd., Cambridge, UK) on a Sprague Dawley (SD) background, following a previously established protocol [53]. *Cacna1c^+/−^* rats [15] exhibit a 4-base-pair (bp) deletion at 460,649–460,652 bp in genomic sequence resulting in an early stop codon in exon 6. Homozygous *Cacna1c^-/-^* rats are embryonically lethal [54]. Rats were bred at the Faculty of Psychology, University of Marburg. A heterozygous breeding protocol was used to obtain offspring from both genotypes, as described previously [15]. The day of birth was designated postnatal day 0. On postnatal day 5 ± 1, rats were marked by paw tattoos and tail snips were collected for genotyping as described previously [15]. The following primers were used: GCTGCTGAGCCTTTTATTGG (*Cacna1c* Cel-1 F) and CCTCCTGGATAGCTGCTGAC (*Cacna1c* Cel-1 R). After weaning on postnatal day 21, rats were socially housed in groups of 4–6 with same-sex littermate partners under standard laboratory conditions (22 ± 2 °C and 40–70% humidity) with free access to standard rodent chow and water. In this study, female rats were investigated at the age of 9–15 months. All experiments, except for immunoblotting, were performed with the experimenter blinded to the genotype of the animals/cells. Rats were anesthetized by isoflurane inhalation (≈2%; FORENE^®^ 100% (*V*/*V*), AbbVie, Wiesbaden, Germany). Deep anesthesia was confirmed by abolished pain reflexes. Rats were sacrificed by decapitation using a guillotine. Following the sacrifice of the animals, hearts were excised quickly and used for tissue or cell isolation.

### 4.2. Left Ventricular Tissue Isolation

Following decapitation, hearts were quickly excised and placed in an ice-cold solution. The chambers (LV) were separated, weighed, frozen in liquid nitrogen, and stored at −80 °C. Tissue lysates were used for investigating protein expression using a standard Western Blot technique.

### 4.3. Isolation of Ventricular Myocytes and Treatment of Whole Hearts

Ventricular myocytes were isolated by means of a standard Langendorff protocol with enzymatic perfusion as previously described [55,56]. For analysis of protein phosphorylation levels, whole hearts were perfused at the Langendorff system with either Isoprenaline (ISO, 100 nM) or control solution (5 min) and afterward cut and frozen as in Section 4.2.

### 4.4. Recording of Isolated Cardiomyocytes

L-type Ca^2+^ current of isolated ventricular myocytes was recorded using whole-cell patch clamp. Ca^2+^ transients (CaTs) were recorded using the linescan mode of a confocal microscope (LSM510, Zeiss, Oberkochen, Germany) or at a 2D-array scanning microscope (VT-Hawk, Visitech, Sunderland, UK). Myocytes were loaded with 6.6 μM Fluo-4/AM (20 min, 20 min de-esterification), field stimulated (40 V) at 1 Hz, and superfused with recording solution containing (mM): 140 NaCl, 5.4 KCl, 1.5 CaCl_2_, 0.5 MgCl_2_, 10 HEPES, 10 glucose, pH 7.4, at RT. By means of a caffeine bolus application (20 mM), SR Ca^2+^ content and fractional SR Ca^2+^ release were estimated. Sarcomere shortening was measured in unloaded myocytes using an IonOptix setup (IonOptix, Dublin, Ireland).

### 4.5. Stressed Conditions

Stress was mimicked by increasing frequencies of stimulation (2 Hz, 4 Hz) or by adding Isoprenaline (ISO, 100 nM) to the recording solution. A concentration-response curve was obtained by usage of ISO concentrations ranging from 1–100 nM.

### 4.6. Statistics

Statistical analysis was performed with GraphPad Prism (San Diego, CA, USA). Data are presented as scatter plots with bar graphs indicating mean ± SEM. The number of cells is provided as “*n*”, and the number of animals as “N”. Normally distributed data sets were compared by means of an unpaired, two-tailed Student’s *t*-test, not-normally distributed data with a Mann–Whitney U test and considered significant when *p* < 0.05. For multiple group comparisons, ANOVA was applied. Concentration-response curves were analyzed with a non-linear four-parameter (4PL) regression model.

## Data Availability

Data is contained within the article or Supplementary Material. Original data sets not shown here are available from the corresponding author upon reasonable request.

## Supplementary Materials

The supporting information can be downloaded at: https://www.mdpi.com/article/10.3390/ijms24129795/s1.

## Abbreviations

AUC, area under the curve; CaMKII, Ca^2+^/calmodulin-dependent protein kinase II; CaT, Ca^2+^ transient; CSQ, calsequestrin; GAPDH, glyceraldehyde 3-phosphate dehydrogenase; ISO, isoprenaline; LTCC, L-type Ca^2+^ channel; LV, left ventricular or left ventricle; NCX, Na^+^/Ca^2+^ exchanger; PKA, protein kinase A or cAMP-dependent protein kinase; PLB, phospholamban; RV, right ventricular or right ventricle; RyR2, ryanodine receptor type 2; SERCA, sarco/endoplasmic reticulum Ca^2+^-ATPase; SL, sarcomere length; SR, sarcoplasmic reticulum; WT, wild-type.
