# Supplementary material for: Calcium Handling Remodeling Underlies Impaired Sympathetic Stress Response in Ventricular Myocardium from Cacna1c Haploinsufficient Rats
Source: Int J Mol Sci. 2023 Jun 6;24(12):9795. doi: 10.3390/ijms24129795 (PMC10298289; doi:10.3390/ijms24129795)
Supplement: Supplementary file 1 [file ijms-24-09795-s001.zip › ijms-2394028-supplementary.pdf]

Supplementary Material

Calcium handling remodeling underlies impaired sympathetic stress response in ventricular myocardium from *Cacna1c* haploinsufficient rats

Hauke Fender[1], Kim Walter[1], Aytug K. Kiper[2,3], Jelena Plačkić[1], Theresa M. Kisko[4], Moria D. Braun[4], Rainer K.W. Schwarting[3,4], Susanne Rohrbach[5], Markus Wöhr[3,4,6,7], Niels Decher[2,3] & Jens Kockskämper[1 *]

[1] Institute of Pharmacology and Clinical Pharmacy, Faculty of Pharmacy,
Biochemical and Pharmacological Center (BPC) Marburg, University of Marburg, Germany
[2] Institute of Physiology and Pathophysiology, Vegetative Physiology, University of Marburg, Germany
[3] Center for Mind, Brain and Behavior (CMBB), University of Marburg, Germany
[4] Behavioral Neuroscience, Experimental and Biological Psychology, University of Marburg, Germany
[5] Institute of Physiology, University of Gießen, Germany
[6] KU Leuven, Faculty of Psychology and Educational Sciences, Research Unit Brain and Cognition, Laboratory of Biological Psychology, Social and Affective Neuroscience Research Group, Belgium
[7] KU Leuven, Leuven Brain Institute, B-3000 Leuven, Belgium

1. Supplementary Methods

1.1 Cardiac tissue isolation for gravimetric analysis and immunoblotting

After decapitation, the heart was excised and the chambers separated. First, the left and right atria were cut off and then the left ventricle (with septum) was separated from the right ventricle, rapidly frozen in liquid nitrogen and stored at -80°C for later analysis.

1.2 Isolation of ventricular myocytes

Ventricular myocytes were isolated by means of a standard Langendorff perfusion protocol with enzymes as described previously [1, 2]. All solutions were based on a standard Tyrode's solution containing (in mM): 130 NaCl, 5.4 KCl, 0.5 $MgCl_2$, 0.33 NaH_2PO_4, 22 Glucose, 25 HEPES, adjusted to pH 7.4 with 1 M NaOH.

Briefly, after decapitation of the rat, the heart was excised and quickly put into ice-cold cardioplegic solution. Here, the heart was cannulated through the aorta and the coronary vessels were flushed with oxygenated cannulation solution (containing 0.15 mM $CaCl_2$ and 2 U/ml heparin) until they were free of blood. The heart was then attached to the Langendorff perfusion system, at which, via a peristaltic pump to enable constant flow, retrograde perfusion was started. The first solution was a Ca^{2+}-free solution (0.4 mM EGTA, 10 mM 2,3-butanedione monoxime (BDM), 2 U/ml heparin, 37°C). After that, an enzyme solution (0.2 mM $CaCl_2$, 10 mM BDM, 37°C) containing a mixture of collagenase II (0.8 mg/ml, Worthington, USA) und protease type XIV (0.05 mg/ml, Sigma-Aldrich, Germany) was used to digest the heart. After sufficient digestion the ventricles were cut off and placed in a bovine serum albumin (BSA)-containing solution to inhibit further enzyme activity (2 mg/ml BSA, 10 mM BDM, 0.5 mM $CaCl_2$, 37°C). The cut ventricles were then filtered through sieve cloths (300 μm pore size, Kobe, Germany) and the cell suspension was given time (10 min) to settle down. The supernatant was exchanged with 1 mM $CaCl_2$ solution (2 mg/ml BSA, without BDM, room temperature (RT)) and, after further 10 minutes, with 1.5 mM $CaCl_2$ solution (without BSA and BDM, RT) to allow for a slow adaptation of the cells to higher Ca^{2+} concentrations.

1.3 Recording of L-type Ca^{2+} current

Isolated ventricular myocytes were placed on 35 mm dishes (Corning). After 15 minutes of settling, whole-cell patch clamp recordings were performed at RT (21-22°C). Pipettes, which were pulled via DMZ Universal Puller (Zeitz, Germany), had a tip resistance of 2.0-3.5 MΩ when filled with the pipette solution containing (in mM): CsCl 140, $MgCl_2$ 2, $CaCl_2$ 1, EGTA 11, and HEPES 10; pH was adjusted to 7.3 with CsOH. Cells were superfused with a bath solution containing (in mM): NaCl 132, CsCl 4.8, $MgCl_2$ 1.2, $CaCl_2$ 1, glucose 5, and HEPES 10; pH was adjusted to 7.3 with CsOH. Series resistance was automatically compensated by 70%. In all experiments, the patch pipette current was zeroed before seal formation. Currents were measured with an EPC10 amplifier (HEKA, Germany). The holding potential was -80 mV. Current-voltage (*I*–*V*) relationships were recorded by a voltage step to -40 mV for 100 ms, followed by 300 ms voltage steps ranging from −40 to +40 mV in 10 mV increments, with a sweep time interval of 7 s. Steady-state L-type Ca currents were measured by a voltage step to -40 mV for 40 ms, followed by a 300 ms voltage step to 0 mV, with a sweep time interval of 1 s. The net Ca^{2+} influx was quantified as area under the curve (AUC) of the inward Ca^{2+} current. The AUC was calculated with Clampfit software (Molecular Devices, USA) as integral function of the inward current over the whole voltage step and normalized to the respective membrane capacitance. The sampling rate was 5 kHz. Data acquisition and command potentials were controlled with a commercial software program, Patchmaster (HEKA). Data analysis was done using the Fitmaster software (HEKA).

1.4 Recording of electrically-stimulated Ca^{2+} transients (CaTs)

Isolated ventricular myocytes were plated on laminin-coated (50 μg/ml) glass-bottomed culture dishes (WillCo, Netherlands) for about 20 minutes. Myocytes were loaded with 6.6 μM Fluo-4/AM (Thermo Fisher Scientific) for 20 minutes and another 20 minutes were allowed for de-esterification. To measure Ca^{2+} transients (CaTs), cells were field stimulated (40 V) at 1 Hz via two platinum electrodes and superfused with recording solution containing (in mM): 140 NaCl, 5.4 KCl, 1.5 $CaCl_2$, 0.5 $MgCl_2$, 10 HEPES, 10 glucose adjusted to pH 7.4 with 1 M NaOH. CaTs were recorded at RT at two different setups.

1.4.1 Linescan confocal imaging

A confocal laser scanning microscope (LSM510, Zeiss, Germany) was utilised in linescan mode to record subcellular CaTs. We used an oil immersion objective lens (Plan-Apochromat, 63x/1.4 Oil) while excitation, by means of an argon ion laser, took place at a wavelength of 488 nm, and fluorescence emission was detected at >505 nm. In linescan mode, a line was placed transversely to the longitudinal axis of the myocyte and this line was then scanned repetitively. Scanning time for one line was 3.07 ms and duration of the scan was set to 5 seconds. Image depth was 12 bit. The diameter of the pinhole was set to result in a thickness of the confocal plane of ≈1 μm.
For illustration purposes (Fig.4A,B, Fig.7A), linescan images were edited using FIJI (ImageJ, Bethesda, USA). Editing consisted of modifying the Display Range and smoothing the image. The same Display Range was used for all linescan images in a given figure.

1.4.2 Fast two-dimensional confocal imaging

In addition, a subset of cells was measured using a fast two-dimensional (2D) array scanning confocal microscope (VT-Hawk, Visitech, UK). With this setup a confocal plane of the cell could be scanned via an array of laser beams. Scanning time (we achieved a frame rate of 55 fps) is still slower than the linescan mode of the LSM510 but the advantage of this setup is to be able to visualize a larger area of the cell. Cells were viewed through an oil immersion objective lens (UPlanSApo, 60x/1.35 Oil) and also excited at 488 nm with an argon laser, field stimulated at 1 Hz and bathed with recording solution. At each time point in the protocol videos of 5 seconds duration were recorded to be able to average at least four transients.

1.5 Analysis of Ca^{2+} transients

CaTs were analysed with FIJI (ImageJ, Bethesda, USA). Firstly, the background was subtracted and then 4-5 CaT traces were averaged. The following parameters of the CaT were analysed: diastolic Ca^{2+} (F_0), which relates to the baseline fluorescence of the CaT; systolic Ca^{2+} (F), which is the peak of the CaT; amplitude (dF), the difference between peak F and baseline F_0; rise time (the time elapsed from 10% to 90% of the shift from baseline to peak), and the time constant of CaT decay, tau of decay, obtained by a mono-exponential fit to the decaying part of the CaT. Fluorescence of the transients was normalized to the resting fluorescence (F_{rest}) of the respective cell obtained in the absence of electrical stimulation.

1.6 Estimating SR Ca^{2+} load and fractional release

For estimation of the SR Ca^{2+} content and fractional SR Ca^{2+} release, we applied a caffeine bolus (20 mM) into the perfusion chamber. To this end, the stimulation of the cells was stopped and, thereafter, the caffeine bolus was quickly applied. Caffeine, at higher millimolar concentrations, leads to the opening of all ryanodine

receptors within the cell, which results in the release of the Ca^{2+} stored within the SR. Thus, the caffeine transient is a measure for SR Ca^{2+} load. Fractional release was estimated by dividing the CaT amplitude by the caffeine amplitude.

1.7 Sarcomere shortening

Unloaded sarcomere shortening of field-stimulated ventricular myocytes was measured using an IonOptix setup (IonOptix, Dublin, Ireland). Cells were field-stimulated (40 V) at 1 Hz by means of a MyoPacer unit and two platinum electrodes and superfused with recording solution. Myocytes were viewed through an objective lens (UApo N340, 40x/1.35 Oil), the region-of-interest (ROI) was chosen so that at least seven sarcomeres inside the myocyte were included. The striation pattern was aligned vertically within the ROI, so that the cell lay parallel within the field of view. The software algorithm recognises the striated pattern of the sarcomeres and determines the average sarcomere length within the ROI. The shortening transients were analysed using the IonWizard software (IonOptix). The following parameters were obtained from the shortening transients: diastolic sarcomere length, systolic sarcomere length, shortening amplitude (i.e. the difference between diastolic and systolic sarcomere length). Fractional shortening (FS) was calculated by relating shortening amplitude to diastolic sarcomere length. Kinetic parameters assessed included rise time (i.e. time to peak 90%) and relaxation time of the shortening (time to baseline 90%).

1.8 Sympathetic stimulation

Sympathetic stress was mimicked by applying the β-adrenergic receptor agonist isoprenaline (ISO, Sigma-Aldrich, Germany) at concentrations from 1-100 nM and/or by increasing stimulation frequency from 1 Hz to 2 Hz and then 4 Hz (for 20 seconds). A stock solution of ISO was made freshly each day before the recordings (1 mM ISO in 5 mM ascorbic acid, protected from light, stored on ice). For analysis of the frequency dependence of CaTs, only those cells which completed the entire protocol, i.e. 1, 2 and 4 Hz stimulation, were included in the analysis.

1.8.1 Isoprenaline concentration-response curves in isolated ventricular myocytes

At the LSM510, CaTs of ventricular myocytes treated with progressively increasing concentrations of ISO were measured in linescan mode to establish a concentration-response curve. In this protocol, cells were continuously stimulated at 1 Hz and ISO concentrations ranging from 1 to 100 nM (1 nM – 3 nM – 10 nM – 30 nM – 100 nM) were applied. Each myocyte was treated with 3 different ISO concentrations for 4 min each. An ISO concentration of 100 nM was always used at the end of the protocol in order to normalise the ISO response (see below). Untreated control cells were subject to rundown of CaT amplitude over the duration of the protocol. CaT rundown averaged 15% after 4 minutes, 24% after 8 minutes, 32% after 12 minutes, or 3.1%/min in WT (n=17) and 12% after 4 minutes, 22% after 8 minutes, 32% after 12 minutes, or 2.8%/min in *Cacna1c*$^{+/-}$ myocytes (n=22). To compensate for this rundown, CaT amplitudes in ISO-treated cells were rundown-corrected. For each ventricular myocyte, the increase in CaT amplitude elicited by a given ISO concentration was normalized to the corresponding effect of 100 nM ISO (= 100%). In order to obtain the concentration-response curves, a non-linear regression analysis was performed in GraphPad Prism using a four parameter logistic (4PL) regression model. The bottom plateau was constrained to 0 and the top plateau to 1. The non-linear regression curves for *Cacna1c*$^{+/-}$ and WT myocytes were then compared by means of an extra sum-of-squares F test.

Similarly, a concentration-response curve was determined for the ISO-mediated increase in fractional shortening at the IonOptix setup. Here, myocytes were stimulated at 1 Hz and treated with progressively

increasing ISO concentrations. No rundown correction was necessary and values for the ISO-induced increase in fractional shortening were not normalized, but rather absolute increases in fractional shortening were determined. Calculation of the concentration-response curves was as described for CaT amplitudes.

1.8.2 Sarcolemmal Ca influx in thapsigargin-treated isolated ventricular myocytes

To measure sarcolemmal Ca influx, myocytes were treated with the SERCA inhibitor thapsigargin (1 µM). For this purpose, cells were incubated with thapsigargin for 20 min after Fluo-4 loading and superfused with thapsigargin and electrically-stimulated at the LSM510 for another 6-12 min before the actual protocol. The goal here was to obtain CaTs that resulted solely from extracellular Ca influx and without the involvement of any Ca release from the SR. After basal measurement of CaT at a stimulation frequency of 0.5 Hz in the presence of thapsigargin, cells were treated with 100 nM ISO for 4 minutes. To confirm that the SR was free of Ca, two caffeine boli were applied at the end of the protocol. CaTs in this protocol were normalized to diastolic fluorescence (Fdiast) in the presence of thapsigargin, as no measurement of resting fluorescence was performed.

1.8.3 Isoprenaline treatment of whole hearts

Whole hearts were isolated as described above and attached to the Langendorff perfusion apparatus. They were perfused with either 100 nM ISO-containing or with control solution to study the ISO-mediated phosphorylation increase of Ca^{2+} handling proteins. Initially, hearts were perfused for 5 minutes with a BDM-containing (1 mg/ml) solution followed by perfusion with either ISO-containing or control solution for another 5 minutes. Strength and frequency of contraction was larger in ISO-containing solution compared to control solution (as judged by eye) confirming the effectiveness of ISO treatment. Afterwards, hearts were removed from the Langendorff apparatus and quickly separated into left and right atrium and left and right ventricle and shock-frozen in liquid nitrogen. For each genotype and condition 8 rats were used. The phosphorylation status of the Ca^{2+} handling proteins from LV tissue was investigated by immunoblotting as described below.

1.9 Immunoblotting (Western blotting)

Left ventricular (LV) tissue was homogenised in a homogenisation buffer containing a mixture of protease and phosphatase inhibitors using micro tissue grinders (Wheaton, UK). Subsequently, protein concentration of homogenates was quantified by means of a BCA assay (Thermo Scientific, USA) in combination with a BSA standard curve. Protein expression/phosphorylation of homogenates was determined using standard immunoblotting (Western blotting).

Proteins were separated by means of SDS-PAGE. For most of the proteins studied, precast gradient gels (4-20% Mini-PROTEAN TGX, Bio-Rad, Germany) were used. For proteins <30 kDa, electrophoresis was performed with Tris-Tricine gels (6% stacking gel, 16% running gel). The samples contained 20 µg of total protein and were prepared with Laemmli buffer containing 5% β-mercaptoethanol. For gradient gels, voltage was set to 90 V for 1 h and then increased to 120 V until sufficient separation was achieved. For Tris-Tricine gels, we started with 100 V for 30 min and then reduced voltage to 80 V until sufficient separation was achieved.

A wet electroblotting technique was used to transfer the proteins to a nitrocellulose membrane with a pore size of 0.45 µm (Bio-Rad). Electrical current was set to 100 mA per gel for 2 hours and subsequently reduced to 15 mA/gel overnight. Transfer took place in a cooling chamber.

Following transfer, membranes were cut between protein bands of interest, so that each part of the membrane could be incubated with the antibody of interest only. This allowed for simultaneous detection of various, differently sized proteins on a single membrane, in order to optimally use and save sample material. This also means that one loading control (e.g. GAPDH) was used for up to three proteins of interest derived from the same membrane. The membranes were then washed with TBST-buffer (3x 10 min) on a rocking platform. Blocking was performed with 5% milk in TBST (skim milk powder, Sigma-Aldrich) for 1 hour at RT. Membranes were washed again (TBST, 3x 10 min) and then incubated with the primary antibody (in 0.5% milk in TBST) overnight. The next day, membranes were washed (TBST, 3x 10 min), then incubated with the secondary antibody (in 0.5% milk in TBST, 1 h, RT) and washed again (TBST, 3x 10 min). Tables S1 and S2 display the list of primary and secondary antibodies used, including source, host species, and dilution. The chemiluminescence reaction was detected using a Chemidoc-XRS system (Bio-Rad) after incubation of the membranes for 1 min with the reagent HRP-Juice or the more sensitive HRP-Juice Plus (PJK GmbH, Germany). Following detection, in some cases the secondary antibody was stripped from the membranes by washing with a stripping buffer (containing glycine, SDS, Tween at pH 2.2, 1x 10 min). Subsequently, membranes were washed again (TBST, 2x 10 min) and incubated over night with a different primary antibody. This was only performed if the two primary antibodies derived from different host species. This allowed for detection of different proteins/phosphorylation sites on a single membrane. In this case, the same loading control was used for both the protein and the phosphorylation site (e.g. actin as control for PLB and pS16 in Figure 3).

Images were analysed with FIJI (ImageJ). Intensity of the protein bands was calculated and normalized to a housekeeping protein (GAPDH, actin, calsequestrin). For comparison of expression and phosphorylation of Ca^{2+} handling proteins between genotypes, each gel was loaded with 4 WT and 4 *Cacna1c*$^{+/-}$ samples. *Cacna1c*$^{+/-}$ signals were normalized to the averaged WT signal of the same membrane (= 100%). The related values from two membranes were then analysed together (8 samples for each genotype). For investigation of the ISO-mediated increase in phosphorylation of Ca^{2+} handling proteins, 4 control samples and 4 ISO-treated samples of the same genotype were blotted on one membrane. Here, the signals from the ISO-treated samples were normalized to the averaged signal from the control samples (= 100%). The related values from two membranes were then analysed together (8 samples for each condition). The genotypes were then analysed for differences in the increase of phosphorylation upon ISO treatment.

All original Western Blot images underlying this study are shown below (see 3. Supplementary Western Blot images). For illustration purposes, from some of these blots areas with the protein bands of interest have been cropped and are presented in Figures 1, 3 and 6 as well as Supplementary Figure S4. Pieces are separated by a white dividing line. In some cases, mirror images of the original Western Blots are shown in order to adhere to a given scheme of probes in the respective figure (e.g. PLB, pS16 and the corresponding actin control in Figure 3).

1.10 Statistics

Statistical analysis was performed with GraphPad Prism (GraphPad Software, San Diego, USA). Data sets were tested for normality with a D'Agostino & Pearson test. Normally distributed data sets were compared by means of an unpaired, two-tailed Student's t test. Not normally distributed data was compared using a Mann-Whitney U test. When multiple groups were compared, ANOVA was applied. Concentration-response curves were analysed with a non-linear regression analysis using a four parameter logistic (4PL) regression model. The fitted curves were then tested for the null hypothesis that logEC50 is the same for all data sets and compared via extra-sum-of-squares F-test. All data is presented as scatter plots with bar graphs indicating mean$\pm$SEM, or as mean$\pm$SEM. Individual p-values are provided in the figures. Differences are considered statistically significant when $p<0.05$. Number of cells is provided as "n", and number of animals as "N". * indicates $p<0.05$, ** indicates $p<0.01$, and *** indicates $p<0.001$.

Table S1. Primary antibodies

Protein (MW)	Antibody	Company, Catalogue number	Host	Dilution	Gel
Actin (44 kDa)	Mouse Anti-Actin, mAB (Clone:C4)	MP Biomedicals LLC, #69100	Mouse	1:100,000	16% Tricine gel
GAPDH (35 kDa)	Anti-GAPDH Mouse mAB (6c5)	Calbiochem, #CB1001	Mouse	1:50,000	4-20% gradient gel
Cav1.2 (250 kDa)	Anti Cav1.2 (*CACNA1C*) Antibody	Alomone, #ACC-003	Rabbit	1:1,000	4-20% gradient gel
CSQ (55 kDa)	Anti-Calsequestrin pAb	Thermo Scientific, #PA1-913	Rabbit	1:2,500	4-20% gradient gel
NCX1 (70-120 kDa)	Anti-NCX1 mAb	Thermo Scientific, #MA1-4672	Mouse	1:1,000	4-20% gradient gel
RyR (565 kDa)	Anti-RyR mAB (C3-33)	Thermo Scientific, #MA3-916	Mouse	1:5,000	4-20% gradient gel
RyR2 pSer2808	Anti-phospho-RyR2 (Ser-2808) pAb	Badrilla, #A010-30	Rabbit	1:5,000	4-20% gradient gel
RyR2 pSer2814	Anti-phospho-RyR2 (Ser-2814) pAb	Badrilla, #A010-31	Rabbit	1:5,000	4-20% gradient gel
SERCA2a (100 kDa)	Anti-SERCA2a pAb	Badrilla, #A010-20	Rabbit	1:5,000	4-20% gradient gel
PLB (6-30 kDa)	Anti-Phospholamban (PLN, PLB) mAb (clone A1)	Badrilla, #A010-14	Mouse	1:5,000	16% Tricine gel
PLB pSer16	Anti-Phospholamban Phospho-Ser16 pAb	Badrilla, #A010-12	Rabbit	1:5,000	16% Tricine gel
PLB pThr17	Anti-Phospholamban Phospho-Thr17 pAb	Badrilla, #A010-13	Rabbit	1:5,000	16% Tricine gel

Table S2. Secondary antibodies

Antibody	Company, Catalogue number	Dilution
Immunopure Goat Anti-Mouse IgG, Peroxidase Conjugated	Thermo Scientific, #31430	1:5,000
Immunopure Goat Anti-Rabbit IgG, Peroxidase Conjugated	Thermo Scientific, #31460	1:5,000

2. Supplementary Figures

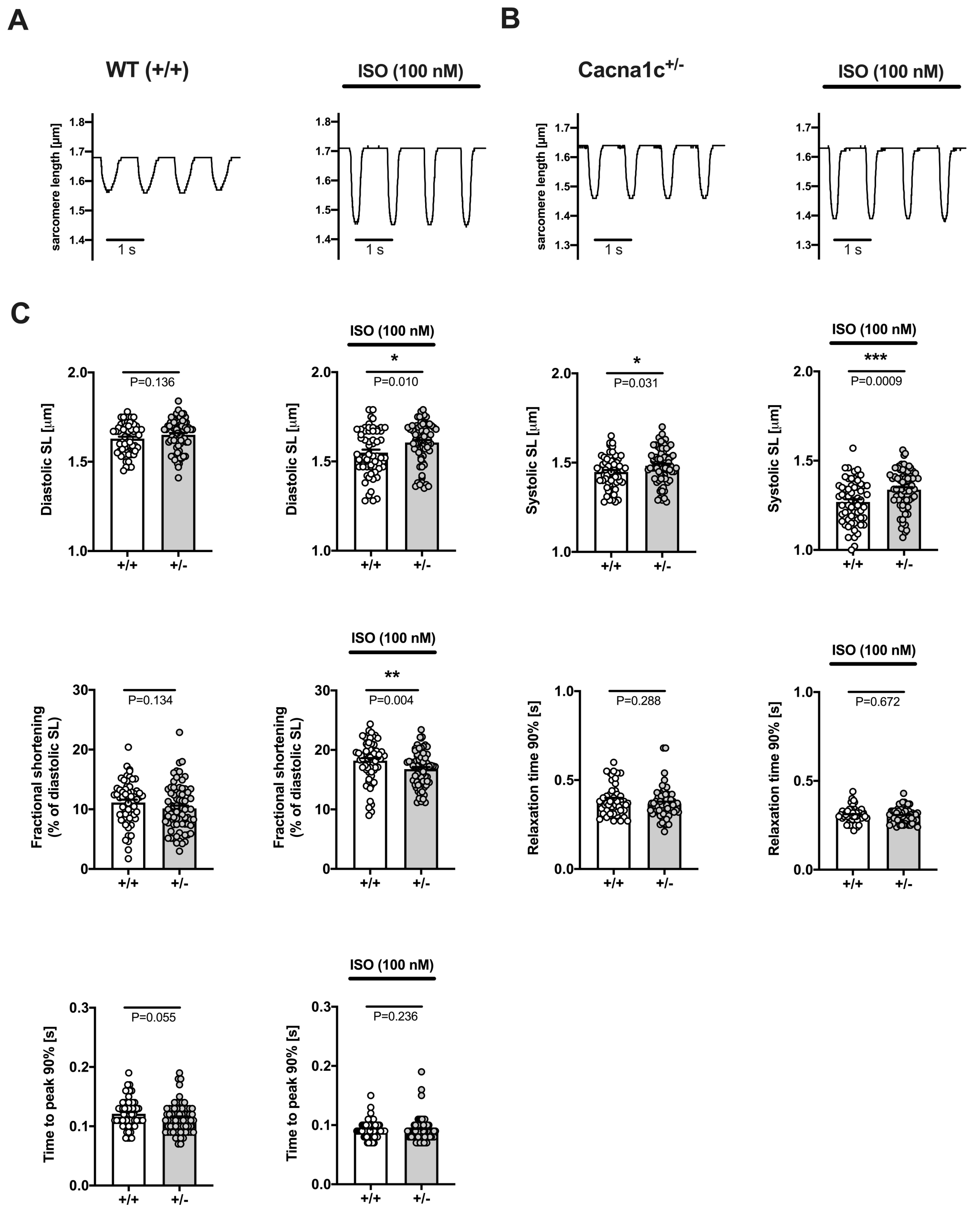

Supplementary Figure S1

Effects of isoprenaline (ISO) on sarcomere shortening of electrically-stimulated ventricular myocytes in WT and *Cacna1c*$^{+/-}$. Representative sarcomere shortenings of a WT (*A*) and *Cacna1c*$^{+/-}$ (*B*) ventricular myocyte before and during exposure to 100 nM ISO. ISO increased and accelerated sarcomere shortenings in both cells. (*C*) Comparison of sarcomere shortening characteristics between WT (+/+) and *Cacna1c*$^{+/-}$ (+/-) ventricular myocytes before (left panels) and during ISO exposure (right panels): (*C*) diastolic SL, systolic SL, fractional shortening, relaxation time 90% and time to peak 90%. In the presence of ISO, systolic SL shortened less and fractional shortening was significantly lower in *Cacna1c*$^{+/-}$. Circles represent individual cardiomyocytes: n=61, N=7 (WT); n=72, N=8 (*Cacna1c*$^{+/-}$); Mann-Whitney U-test; except for systolic SL, relaxation time 90 % (100 nM) and fractional shortening (Student's t-test), p-values as indicated.

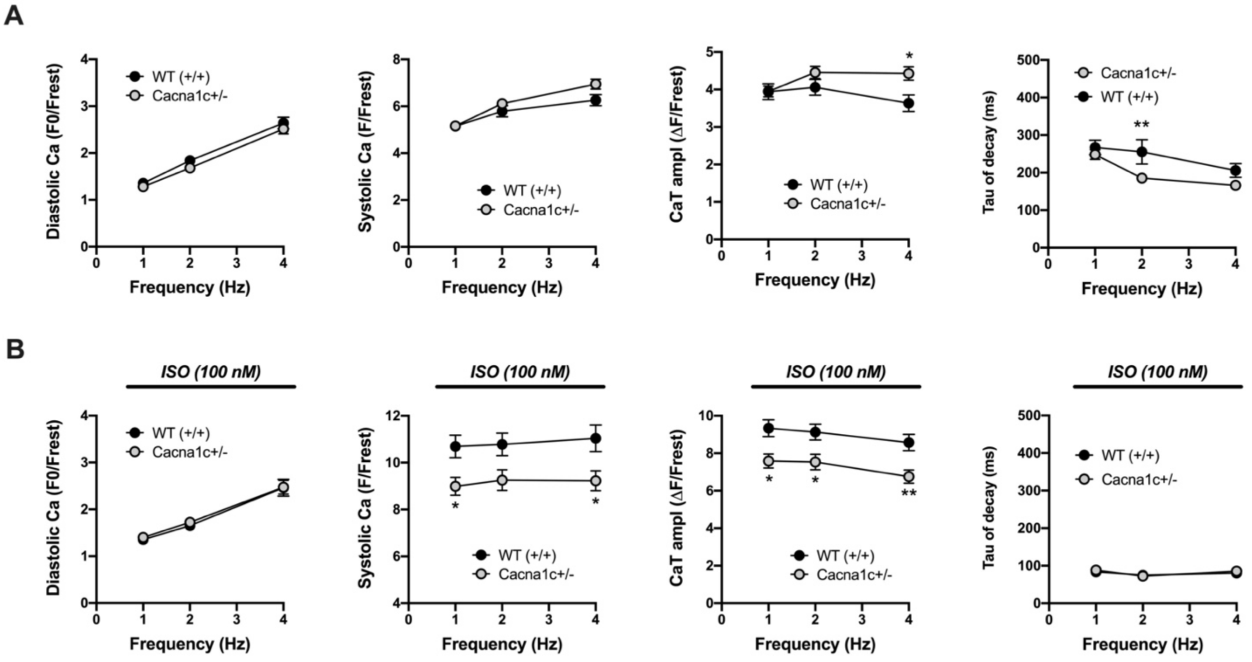

Supplementary Figure S2

Frequency dependence of CaTs in untreated ventricular myocytes (*A*) and during exposure to 100 nM ISO (*B*). Comparison of diastolic Ca, systolic Ca, CaT amplitude and tau of decay in WT (+/+, black) and *Cacna1c*$^{+/-}$ (+/-, grey). (*A*) At 4 Hz frequency, CaT amplitude was significantly higher in *Cacna1c*$^{+/-}$. (*B*) In the presence of ISO, systolic Ca as well as CaT amplitude was significantly smaller in *Cacna1c*$^{+/-}$ at most frequencies studied (1 Hz, 2 Hz, 4 Hz). Number of cardiomyocytes (*A*): n=49, N=8 (WT); n=91, N=11 (*Cacna1c*$^{+/-}$) and (*B*): n=18, N=5 (WT); n=32, N=8 (*Cacna1c*$^{+/-}$). Two-way ANOVA followed by Bonferroni's multiple comparisons test. * $p<0.05$, ** $p<0.01$

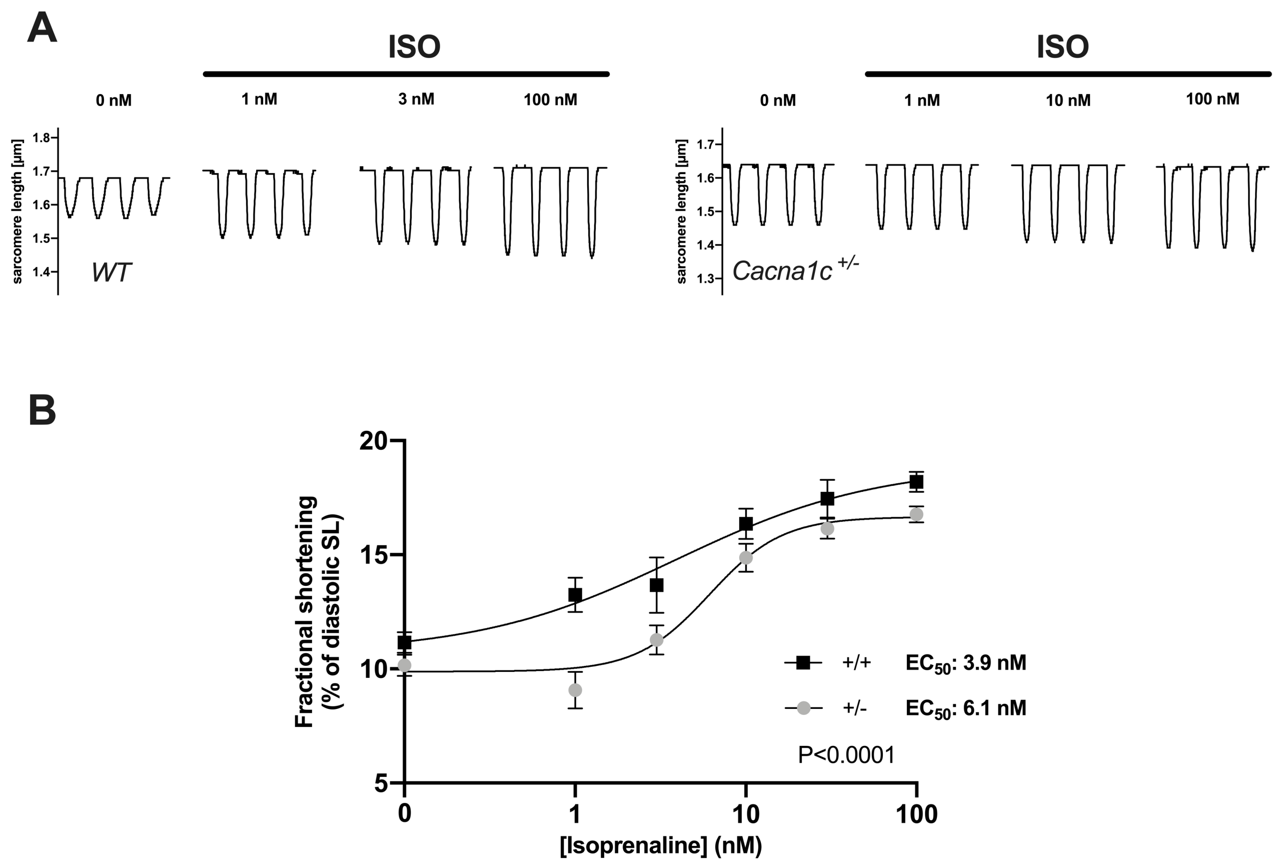

Supplementary Figure S3

Concentration dependence of isoprenaline (ISO) effect on fractional sarcomere shortening in WT and *Cacna1c*$^{+/-}$ ventricular myocytes. (*A*) Example sarcomere shortenings from one WT (left) and one *Cacna1c*$^{+/-}$ (right) ventricular myocyte treated with increasing concentrations of ISO (as indicated). (*B*) Resulting concentration-response curves for WT (+/+, squares) and *Cacna1c*$^{+/-}$ (+/-, circles) ventricular myocytes. Number of cells and animals (WT/*Cacna1c*$^{+/-}$): 0 nM: n=61/72, N=7/8, 1 nM: n=27/25, N=3/3, 3 nM: n=19/43, N=2/5, 10 nM: n=36/23, N=5/3, 30 nM: n=28/42, N=4/5, 100 nM: n=61/72, N=7/8. In *Cacna1c*$^{+/-}$ myocytes, concentration-response curve was shifted to the right. EC_{50} values: 3.9 nM (WT, +/+) and 6.1 nM (*Cacna1c*$^{+/-}$, +/-).

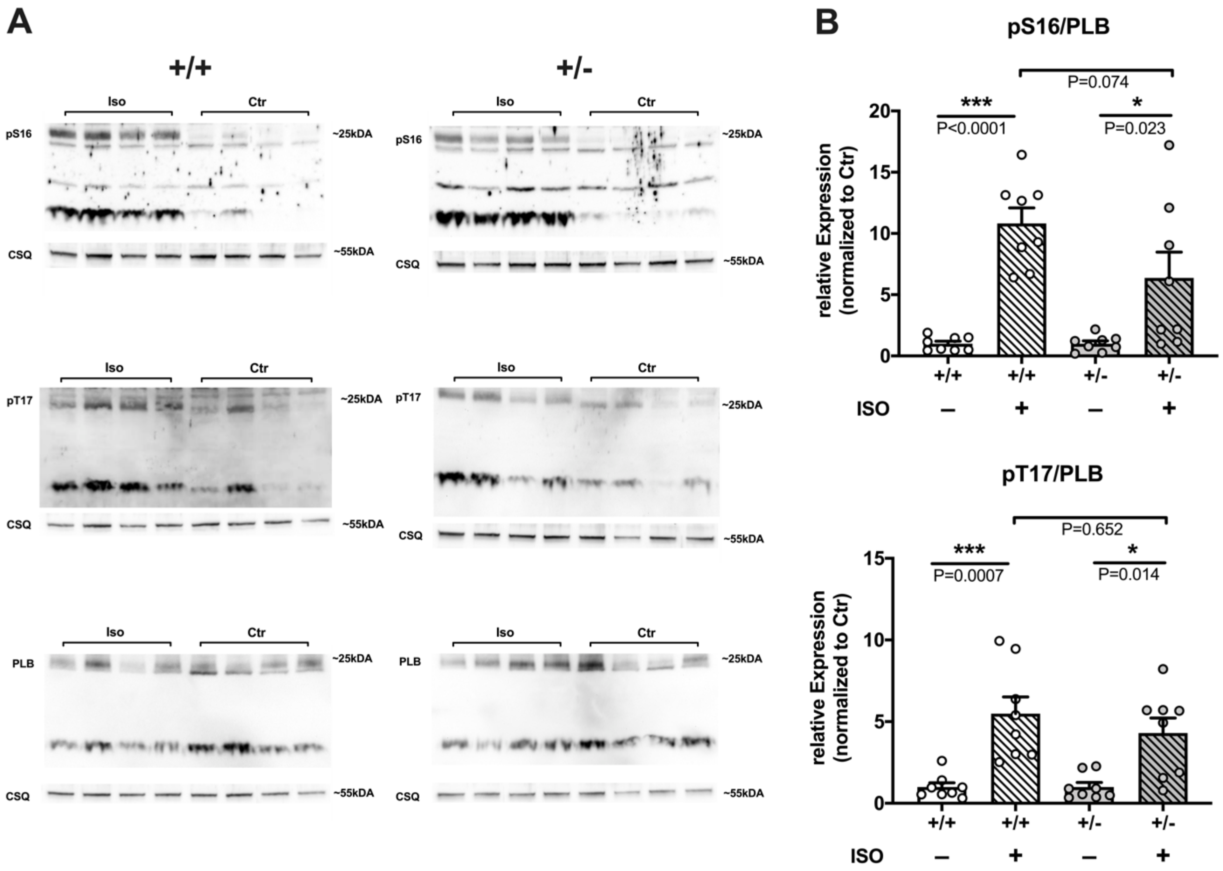

Supplementary Figure S4

Phosphorylation of PLB in WT (+/+) and *Cacna1c*$^{+/-}$ (+/-) LV myocardium treated with 100 nM isoprenaline (ISO). Untreated LV myocardium served as control (Ctr). (*A*) Phosphorylation of PLB at S16 (top) and at T17 (middle) as well as total PLB (bottom). Original immunoblot images (*A*) and summarised data (*B*) is shown. One representative Western Blot is presented for each protein/phosphorylation site and genotype with 4 Ctr and 4 ISO-treated samples. CSQ was used for normalisation. Data is normalized to the average value of Ctr (= 100%). Circles represent number of animals: N=8 for each genotype (WT, *Cacna1c*$^{+/-}$) and treatment (Ctrl vs ISO); One-way ANOVA followed by Tukey's post-hoc test, p-values as indicated.

3. Supplementary Western Blot images

3.1 Original Western Blot images from Figures 1 and 3

Cav1.2 (from right to left: 4 wildtype samples and 4 *Cacna1c*$^{+/-}$ samples from 8 different animals on each blot, two blots. Membranes were cut before antibody application. Upper membrane contains samples #1-4 of each genotype, lower membrane samples #5-8 of each genotype. Upper membrane (cropped) is presented in Figure 1.)

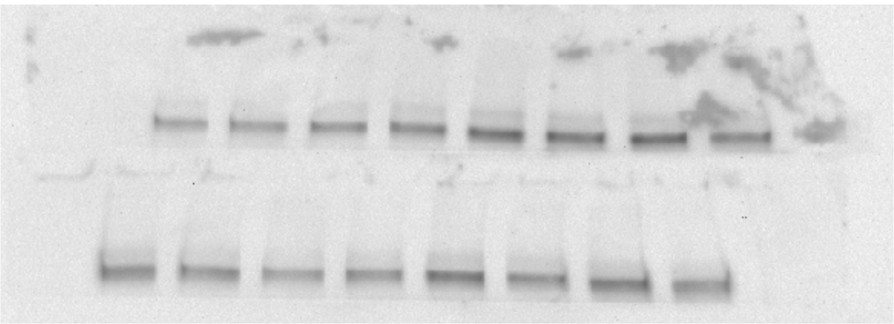

Associated GAPDH membranes (Upper membrane contains samples #1-4 of each genotype, lower membrane samples #5-8 of each genotype. Upper membrane (cropped) is presented in Figure 1.)

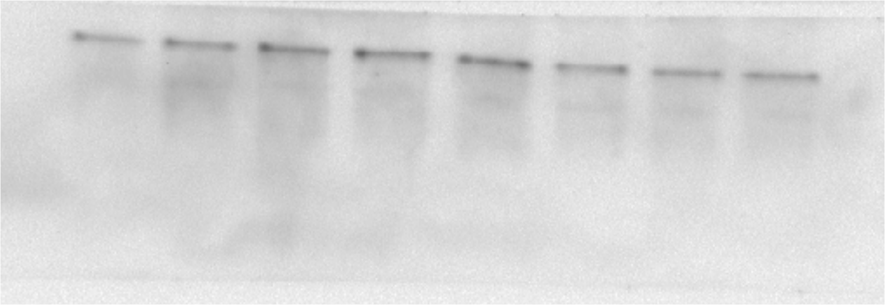

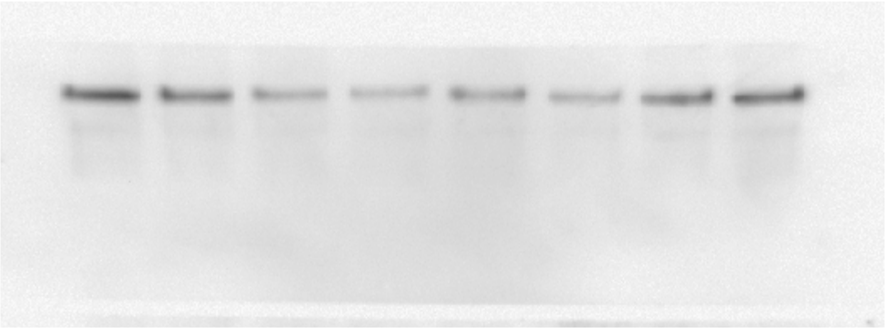

Calsequestrin (from right to left: 4 wildtype samples and 4 *Cacna1c*$^{+/-}$ samples from 8 different animals on each blot, two blots. Membranes were cut before antibody application. The sample far left is a ventricular pool sample. Upper membrane contains samples #1-4 of each genotype, lower membrane samples #5-8 of each genotype. Upper membrane (cropped) is presented in Figure 3.)

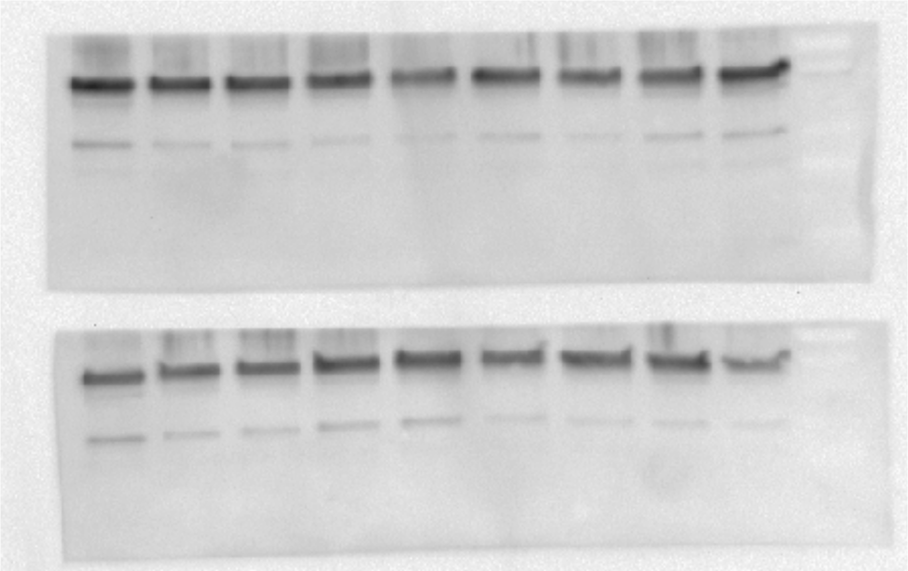

SERCA2a (from right to left: 4 wildtype samples and 4 *Cacna1c*$^{+/-}$ samples from 8 different animals on each blot, two blots. Membranes were cut before antibody application. The sample far left is a ventricular pool sample. Upper membrane contains samples #1-4 of each genotype, lower membrane samples #5-8 of each genotype. Lower membrane (cropped) is presented in Figure 3.)

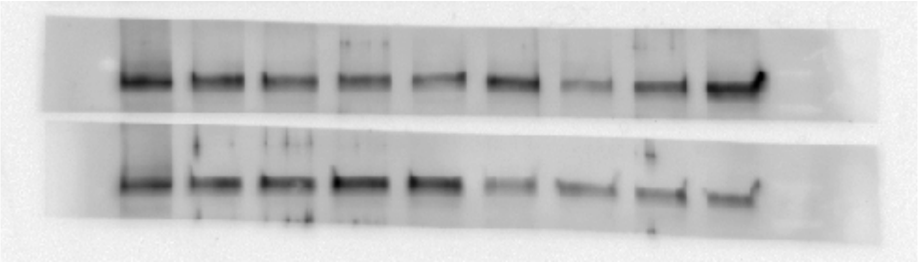

Associated GAPDH membranes (CSQ, SERCA2a and pS2808 (see below) share the same GAPDH signal, because they are derived from the same blot. The sample far left is a ventricular pool sample. Upper membrane contains samples #1-4 of each genotype, lower membrane samples #5-8 of each genotype. Upper membrane (cropped) is presented in Figure 3 as control for CSQ and pS2808. Lower membrane (cropped) is presented in Figure 3 as control for SERCA2a.)

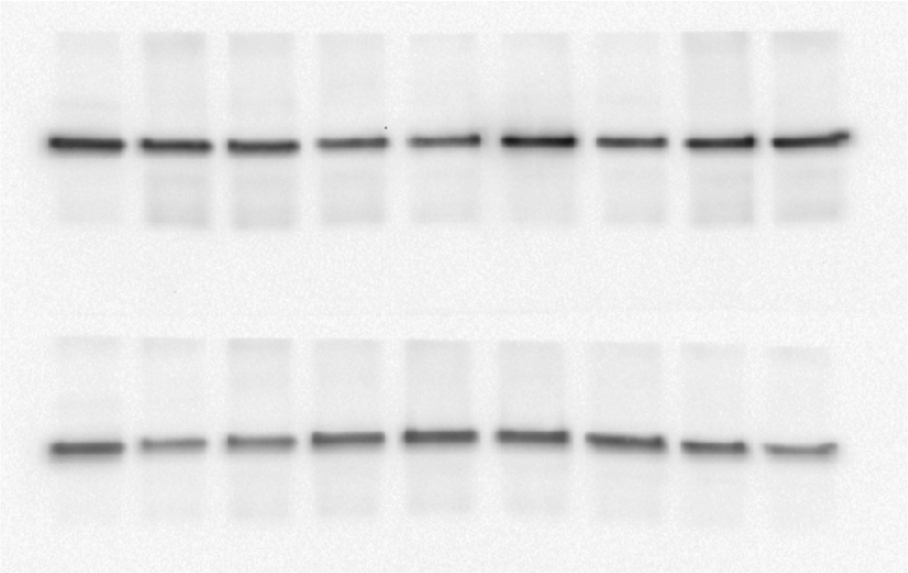

NCX (from right to left: 4 wildtype samples and 4 *Cacna1c*$^{+/-}$ samples from 8 different animals on each blot, two blots. Membranes were cut before antibody application. The sample far left is a ventricular pool sample. Upper membrane contains samples #1-4 of each genotype, lower membrane samples #5-8 of each genotype. Upper membrane (cropped) is presented in Figure 3.)

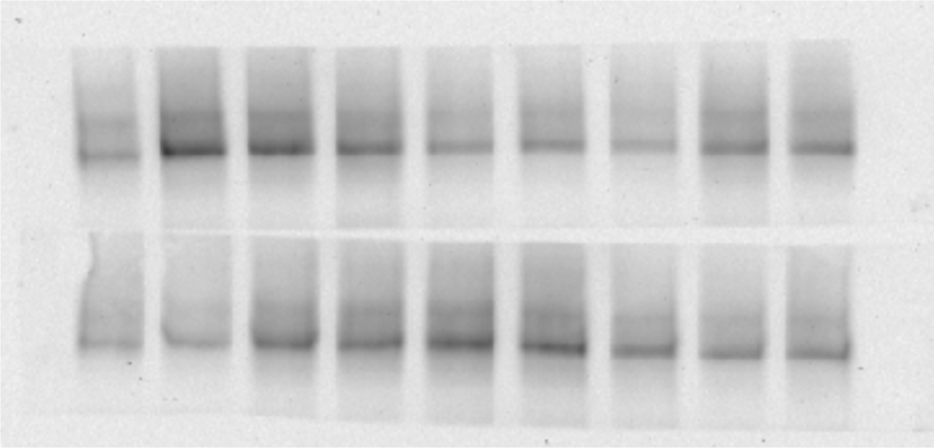

Associated GAPDH membranes. The sample far left is a ventricular pool sample. (Upper membrane contains samples #1-4 of each genotype, lower membrane samples #5-8 of each genotype. Upper membrane (cropped) is presented in Figure 3.)

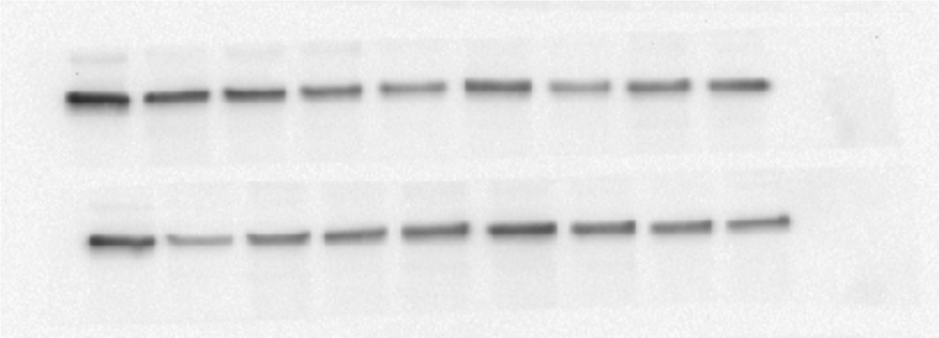

Phospholamban (from left to right: 4 wildtype samples and 4 *Cacna1c*$^{+/-}$ samples from 8 different animals on each blot, two blots. Membranes were cut before antibody application. Upper membrane contains samples #1-4 of each genotype, lower membrane samples #5-8 of each genotype. Upper membrane (cropped, mirror image) is presented in Figure 3.)

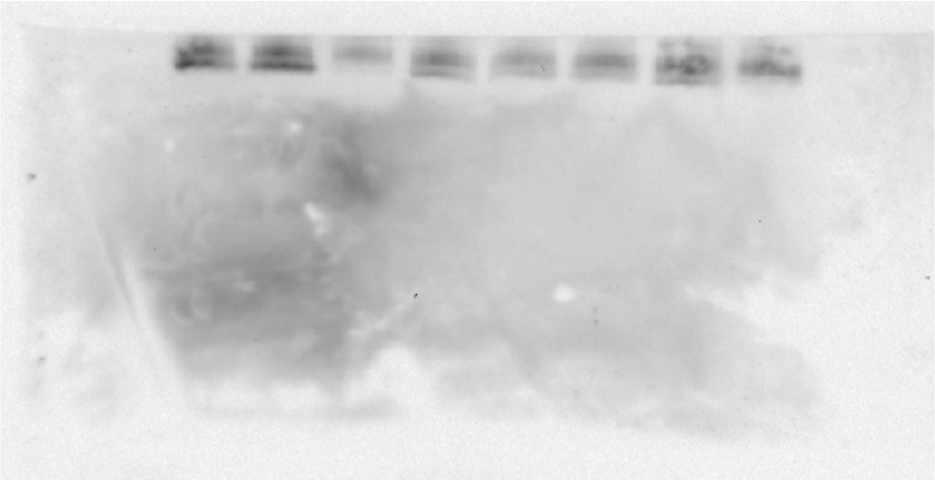

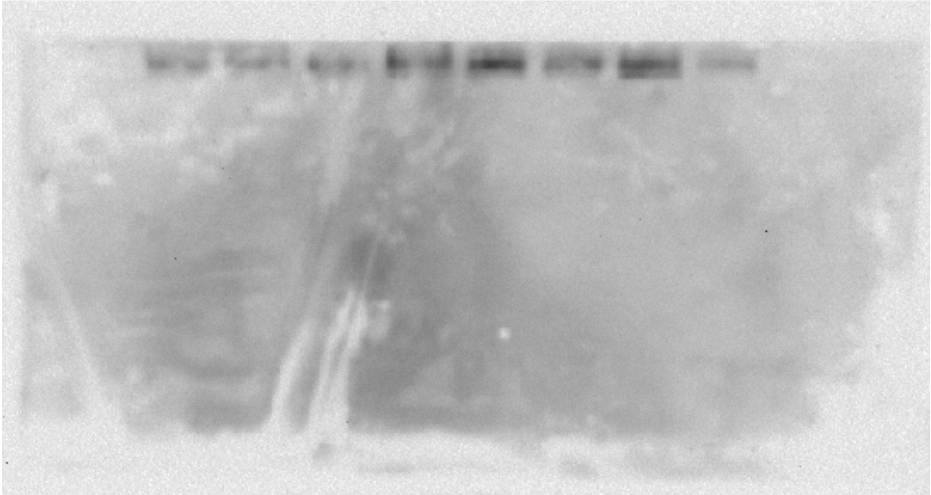

Associated Actin membranes (These are the same actin membranes as for pS16, because in this case stripping was performed. Upper membrane contains samples #1-4 of each genotype, lower membrane samples #5-8 of each genotype. Upper membrane (cropped, mirror image) is presented in Figure 3.)

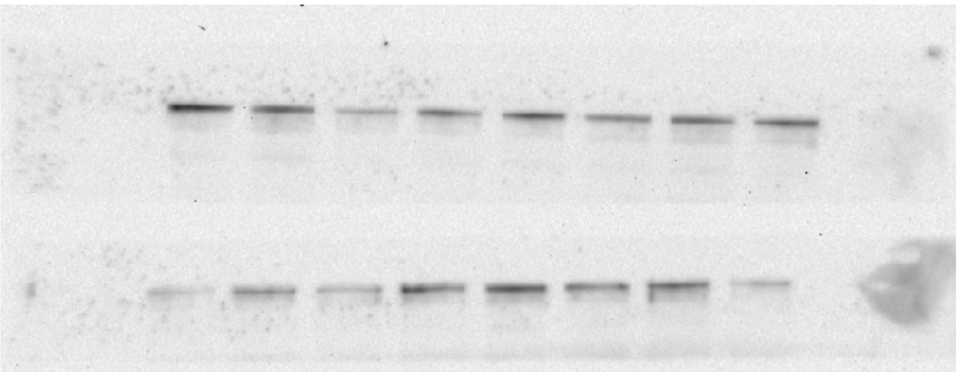

RyR2 (from right to left: 4 wildtype samples and 4 *Cacna1c*$^{+/-}$ samples from 8 different animals on each blot, two blots. Membranes were cut before antibody application. Upper membrane contains samples #1-4 of each genotype, lower membrane samples #5-8 of each genotype. Upper membrane (cropped) is presented in Figure 3.)

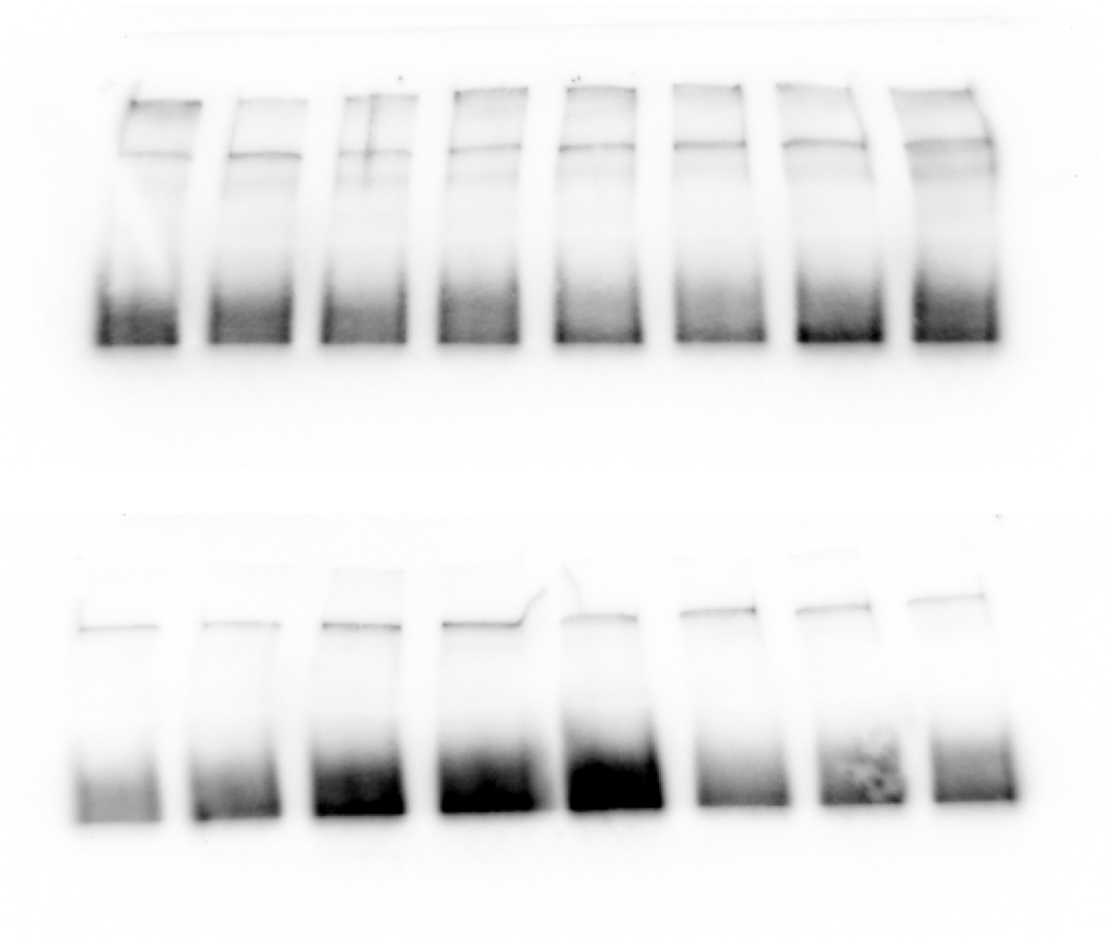

Associated GAPDH membranes.Upper membrane contains samples #1-4 of each genotype, lower membrane samples #5-8 of each genotype. Upper membrane (cropped) is presented in Figure 3.)

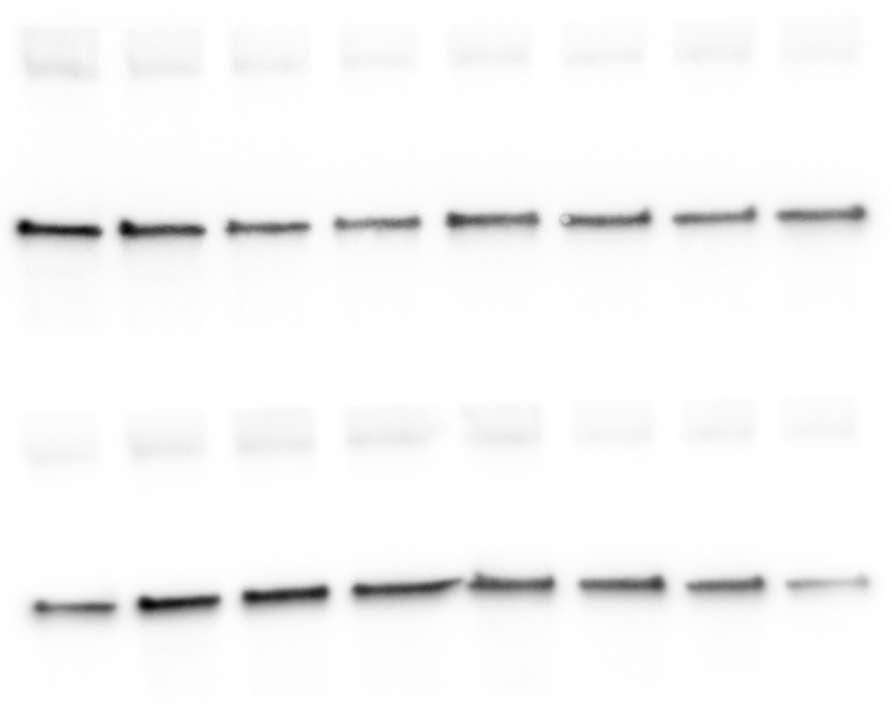

pS2808 (from right to left: 4 wildtype samples and 4 *Cacna1c*$^{+/-}$ samples from 8 different animals on each blot, two blots. Membranes were cut before antibody application. Upper band was analyzed. The sample far left is a ventricular pool sample. Upper membrane contains samples #1-4 of each genotype, lower membrane samples #5-8 of each genotype. Upper membrane (cropped) is presented in Figure 3.)

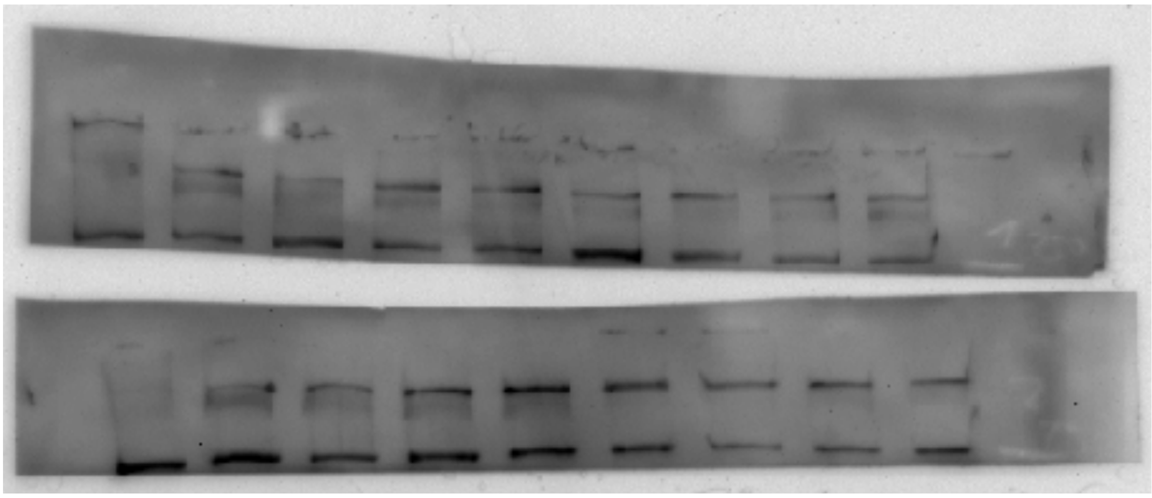

Associated GAPDH membranes (CSQ, SERCA2a (see above) and pS2808 share the same GAPDH signal, because they are derived from the same blot. The sample far left is a ventricular pool sample. Upper membrane contains samples #1-4 of each genotype, lower membrane samples #5-8 of each genotype. Upper membrane (cropped) is presented in Figure 3 as control for CSQ and pS2808. Lower membrane (cropped) is presented in Figure 3 as control for SERCA2a.)

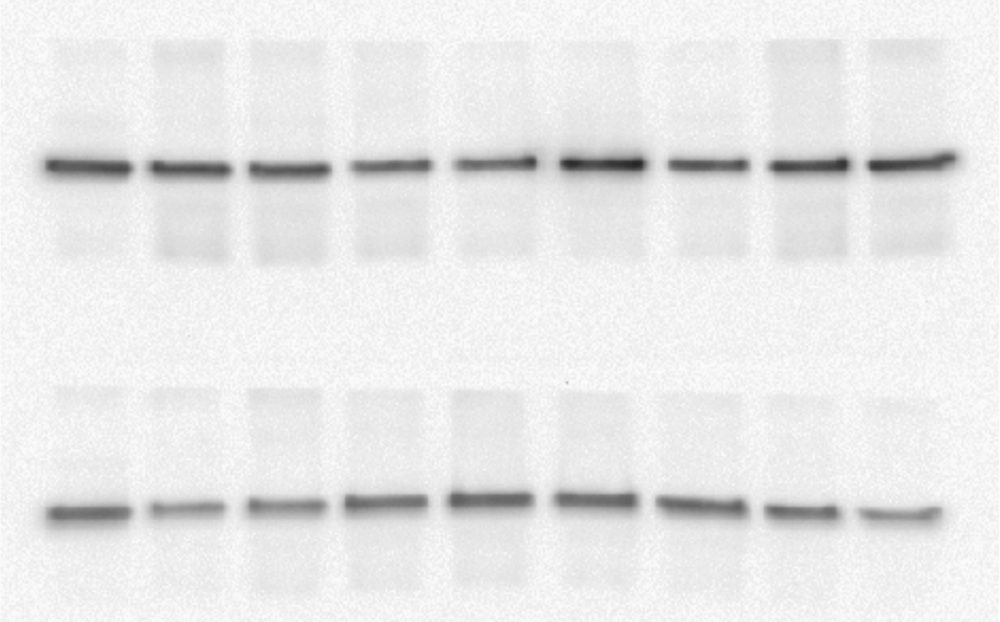

pS2814 (from left to right: 4 wildtype samples and 4 *Cacna1c*$^{+/-}$ samples from 8 different animals on each blot, two blots. Membranes were cut before antibody application. Upper band was analyzed. Upper membrane contains samples #1-4 of each genotype, lower membrane samples #5-8 of each genotype. Upper membrane (cropped, mirror image) is presented in Figure 3.)

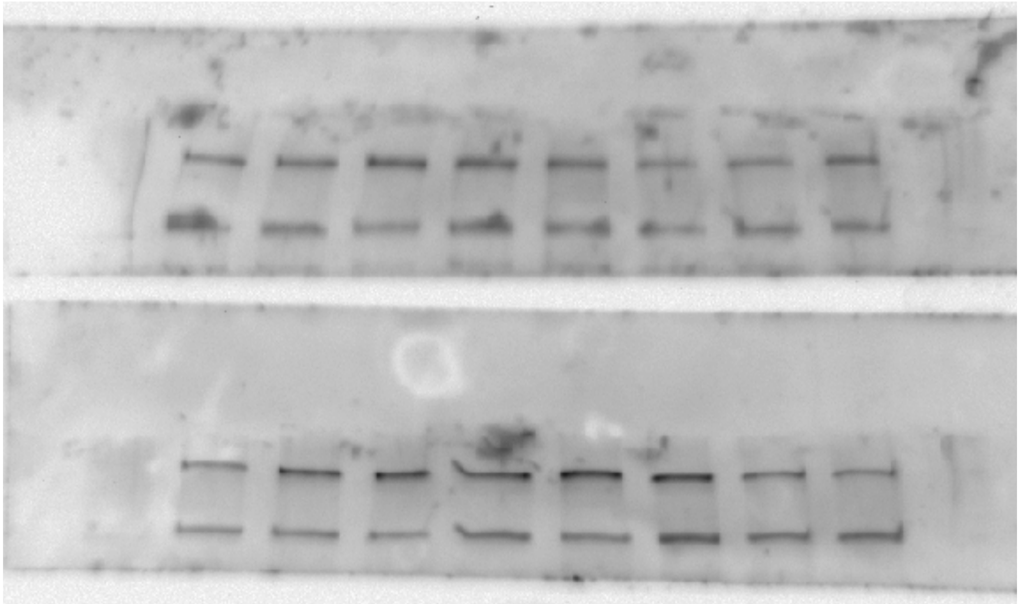

Associated GAPDH membranes (Upper membrane contains samples #1-4 of each genotype, lower membrane samples #5-8 of each genotype. Upper membrane (cropped, mirror image) is presented in Figure 3.)

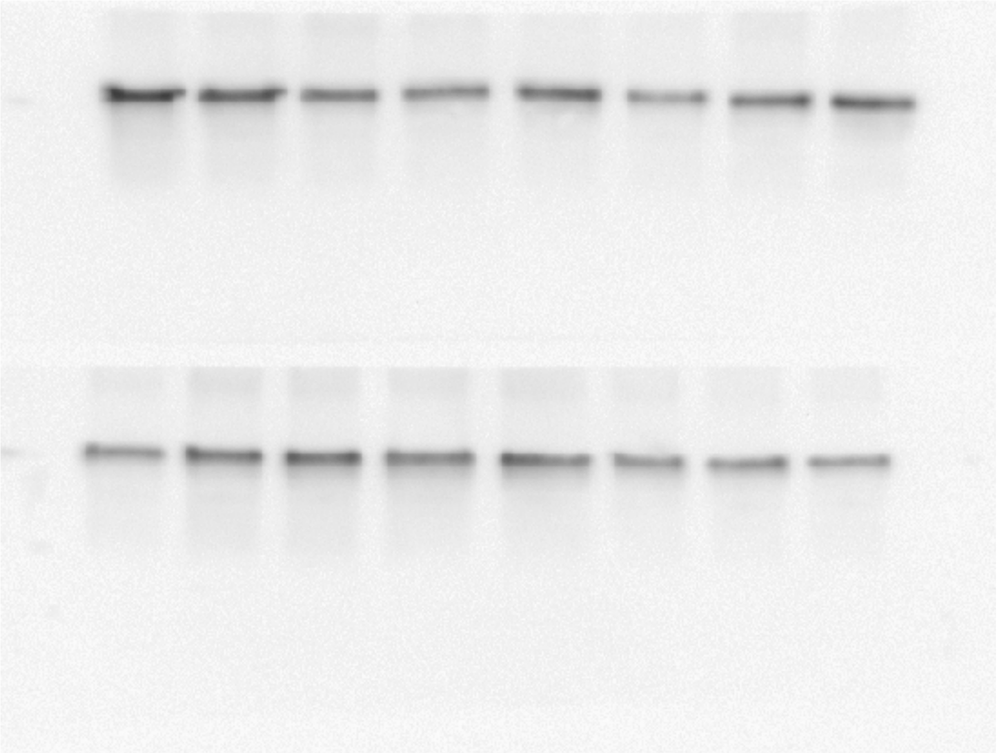

pS16 (from left to right: 4 wildtype samples and 4 *Cacna1c*$^{+/-}$ samples from 8 different animals on each blot, two blots. Membranes were cut before antibody application. Upper membrane contains samples #1-4 of each genotype, lower membrane samples #5-8 of each genotype. Upper membrane (cropped, mirror image) is presented in Figure 3.)

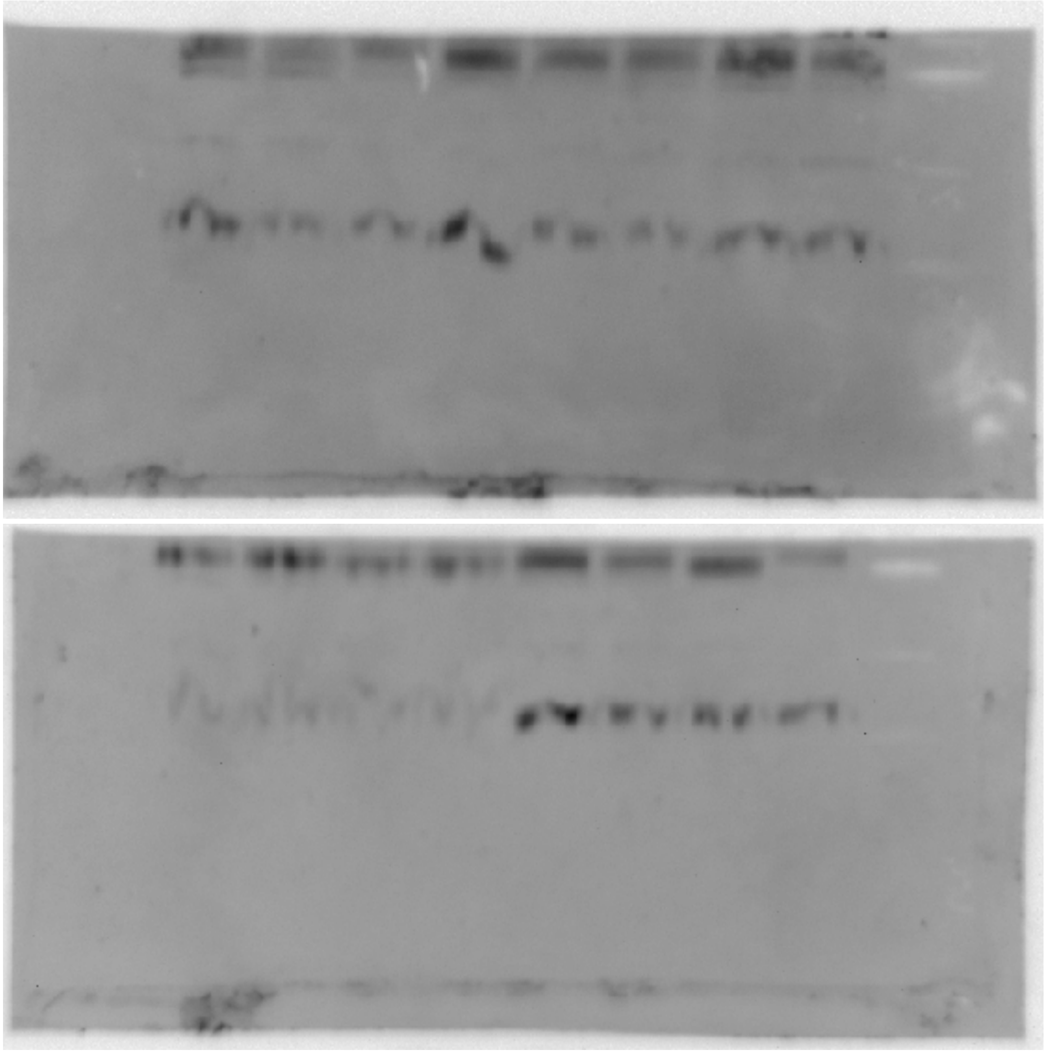

Associated Actin membranes (These are the same actin membranes as for PLB, because in this case stripping was performed. Upper membrane contains samples #1-4 of each genotype, lower membrane samples #5-8 of each genotype. Upper membrane (cropped, mirror image) is presented in Figure 3.)

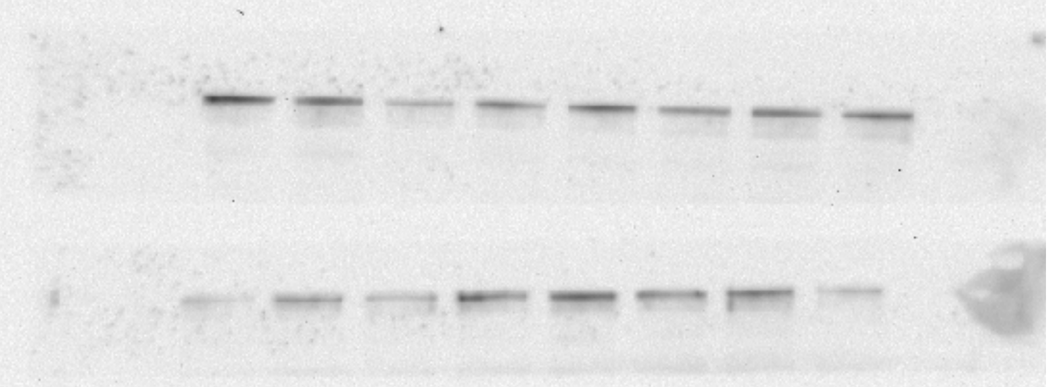

pT17 (from right to left: 4 wildtype samples and 4 *Cacna1c*$^{+/-}$ samples from 8 different animals on each blot, two blots. Lane far right is protein ladder. Upper membrane contains samples #1-4 of each genotype, lower membrane samples #5-8 of each genotype. Upper membrane (cropped) is presented in Figure 3.)

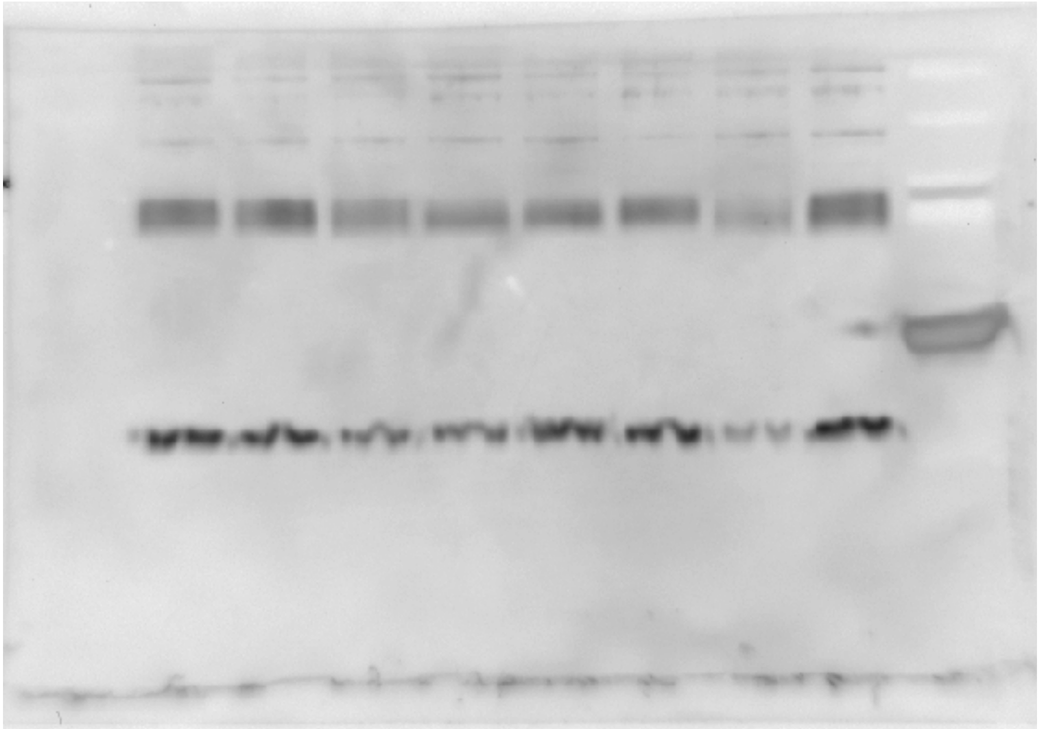

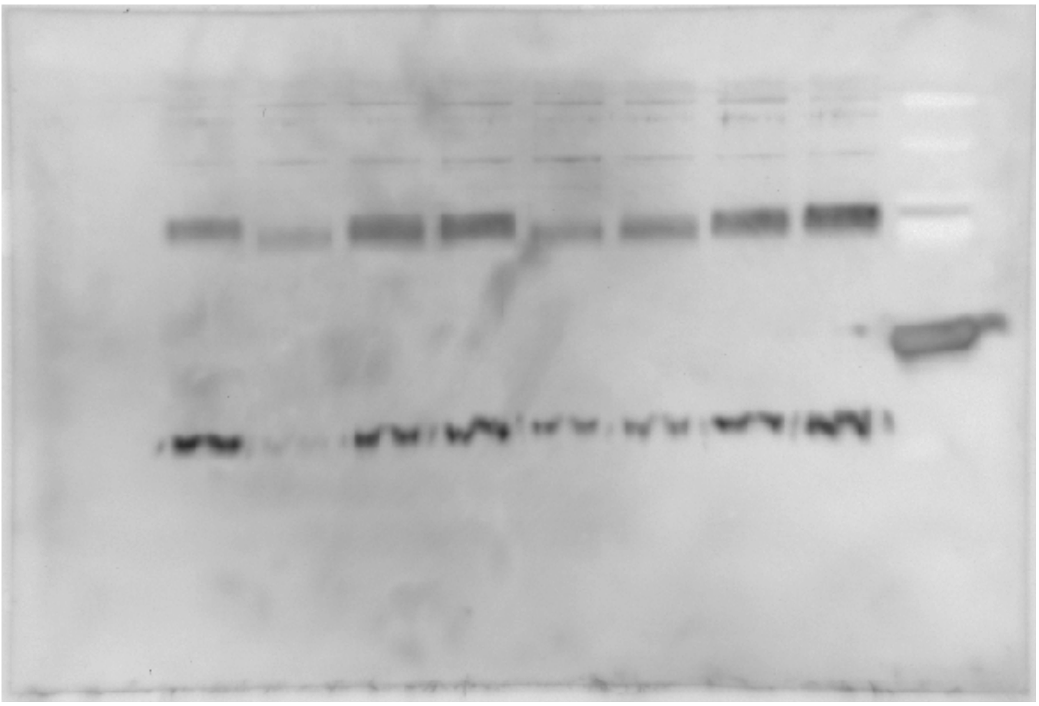

Associated Actin membranes: (after detection of pT17 the anti-actin antibody was put on the same membrane. The second highest band (at approx. 42 kDA) was analyzed. Upper membrane contains samples #1-4 of each genotype, lower membrane samples #5-8 of each genotype. Upper membrane (cropped) is presented in Figure 3.)

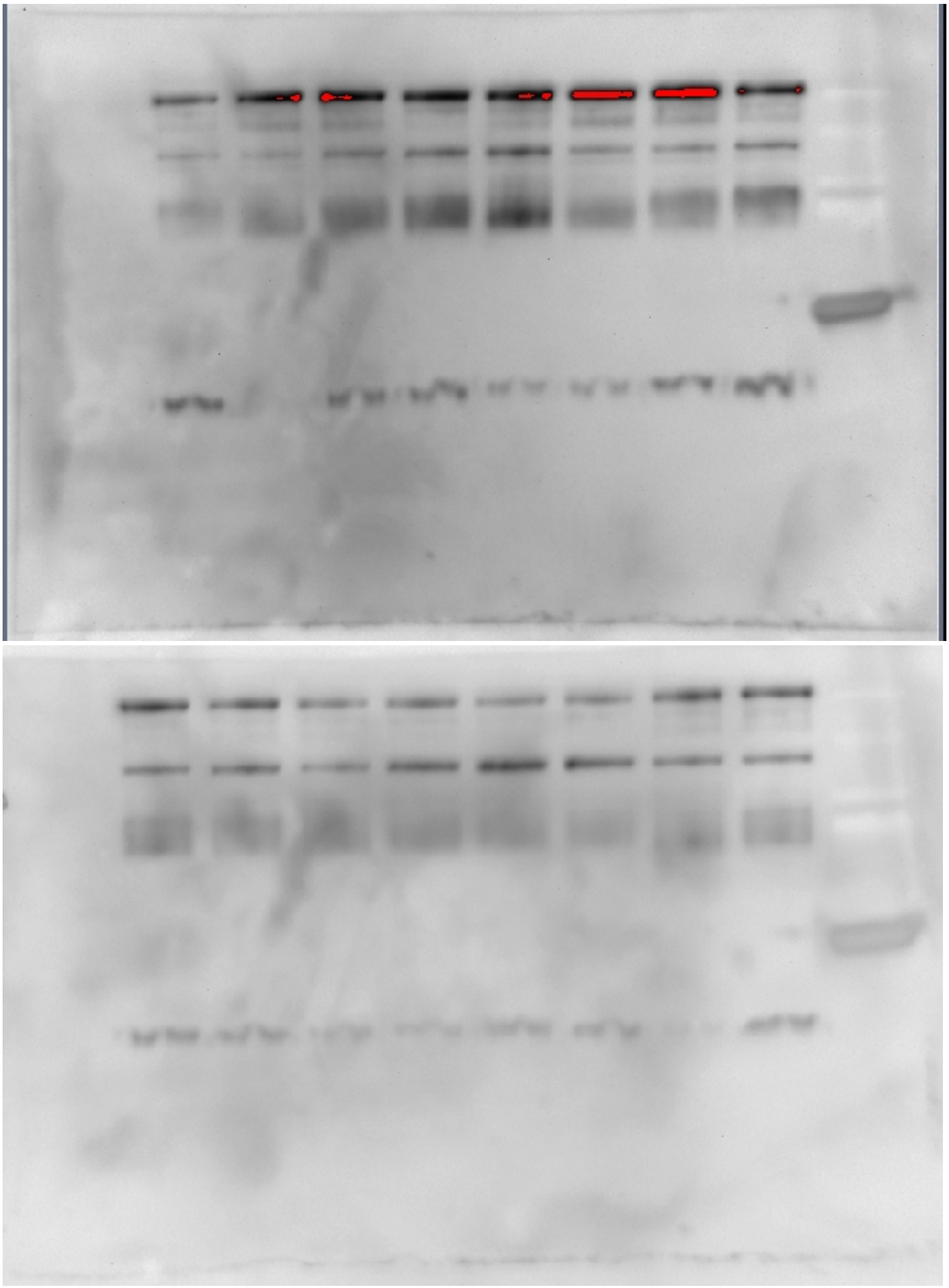

3.2 Original Western Blot images from Figure 6 and Supplementary Figure S4

RyR2 (Wildtype samples. From right to left: 4 control condition samples and 4 isoprenaline treated samples from 8 different animals on each blot, two blots. Membranes were cut before antibody application. The upper membrane contains samples #1-4 for each condition, the lower membrane samples #5-8 for each condition. The lower membrane (cropped) is presented in Figure 6A.)

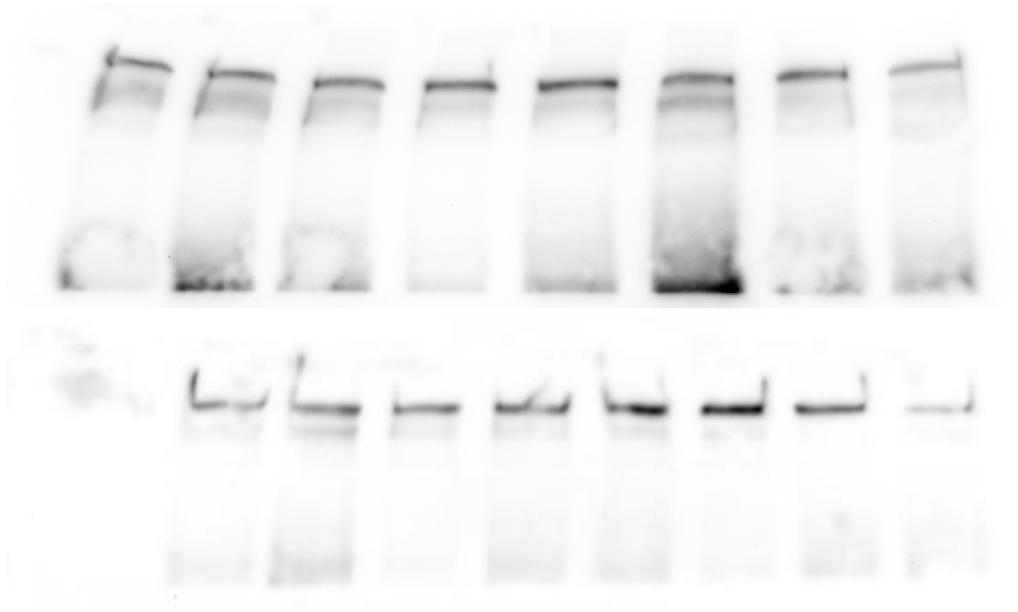

Associated GAPDH membranes: (These are the same GAPDH membranes as for pS2808, because in this case stripping was performed. The upper membrane contains samples #1-4 for each condition, the lower membrane samples #5-8 for each condition. The lower membrane (cropped) is presented in Figure 6A.)

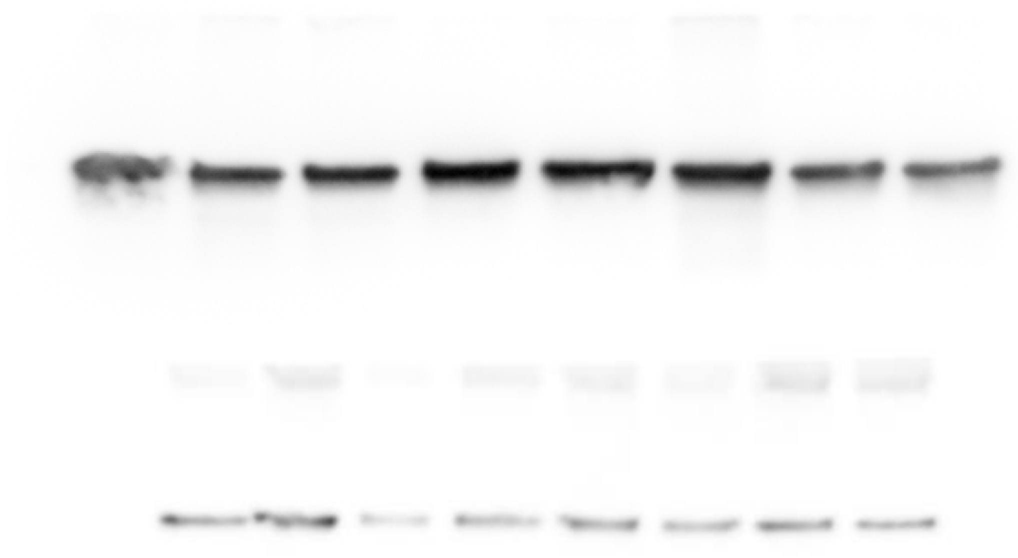

RyR2 (*Cacna1c*$^{+/-}$ samples. From right to left: 4 control condition samples and 4 isoprenaline treated samples from 8 different animals on each blot, two blots. Membranes were cut before antibody application. The sample far left on the second membrane is a ventricular pool sample. The upper membrane contains samples #1-4 for each condition, the lower membrane samples #5-8 for each condition. The lower membrane (cropped) is presented in Figure 6A.)

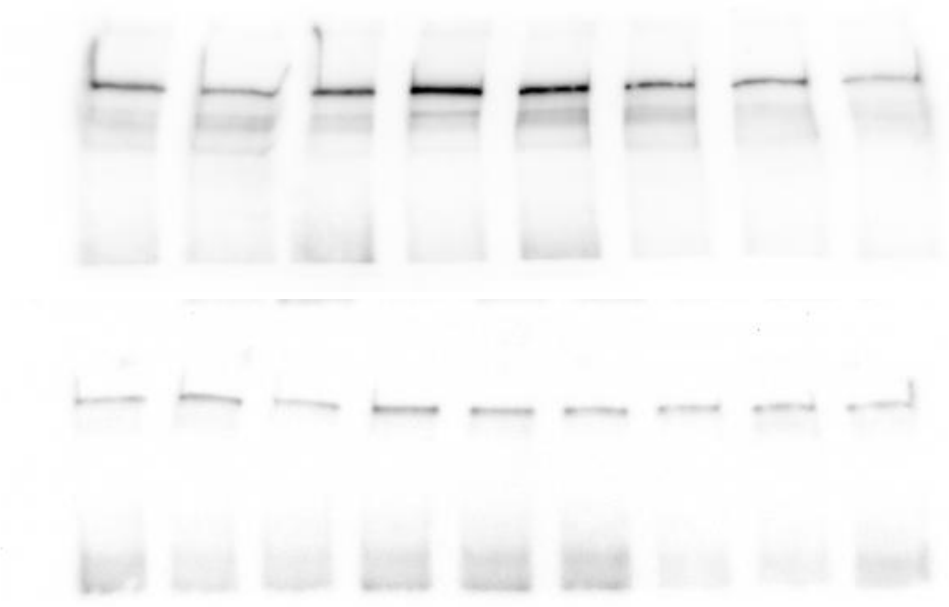

Associated GAPDH membranes (The upper membrane is the same GAPDH membrane as for pS2814 and the lower membrane is the same GAPDH membrane as for pS2808, because in these cases stripping was performed. The sample far left on the second membrane is a ventricular pool sample. The upper membrane contains samples #1-4 for each condition, the lower membrane samples #5-8 for each condition. The lower membrane (cropped) is presented in Figure 6A.)

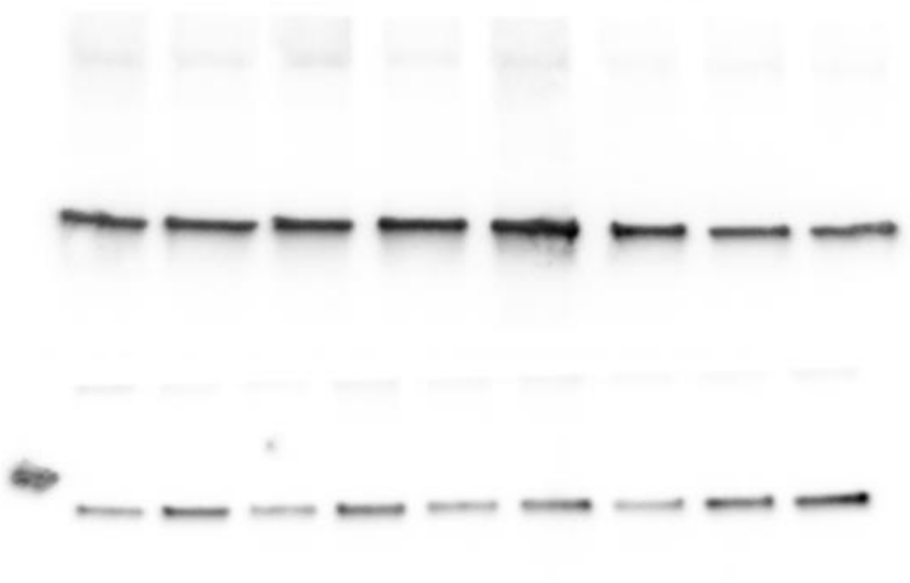

pS2814 (Wildtype samples. From right to left: 4 control condition samples and 4 isoprenaline treated samples from 8 different animals on each blot, two blots. Membranes were cut before antibody application. The upper band was analyzed. The upper membrane contains samples #1-4 for each condition, the lower membrane samples #5-8 for each condition. The lower membrane (cropped) is presented in Figure 6A.)

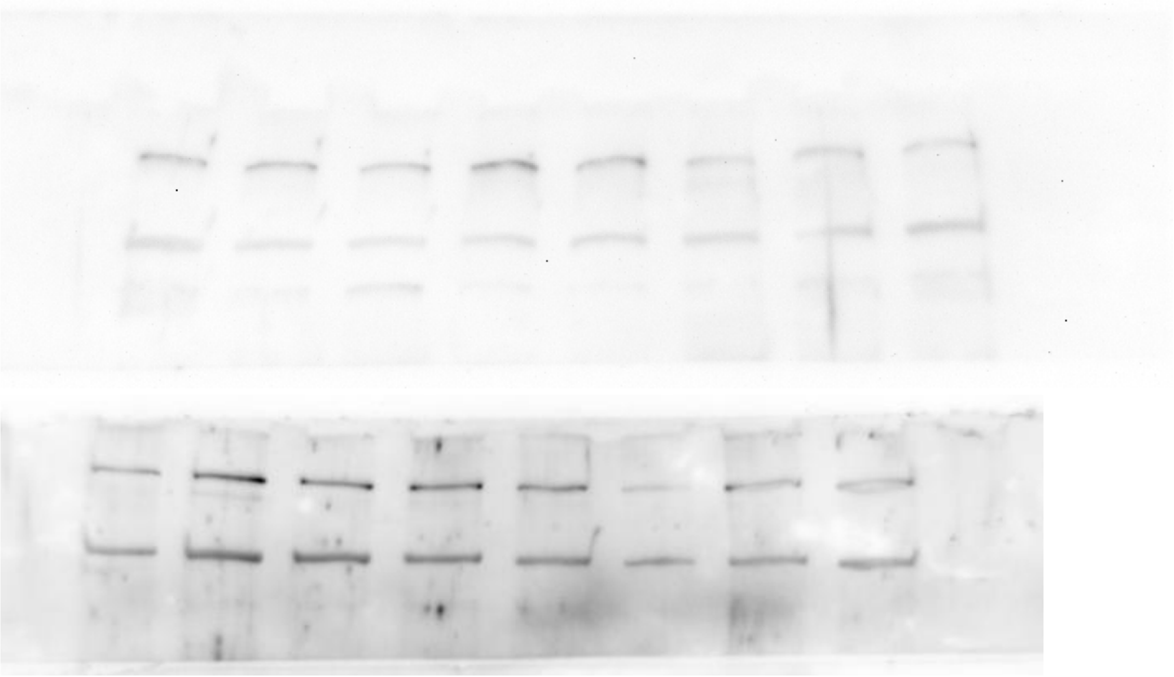

Associated GAPDH membranes (The upper membrane contains samples #1-4 for each condition, the lower membrane samples #5-8 for each condition. The lower membrane (cropped) is presented in Figure 6A.)

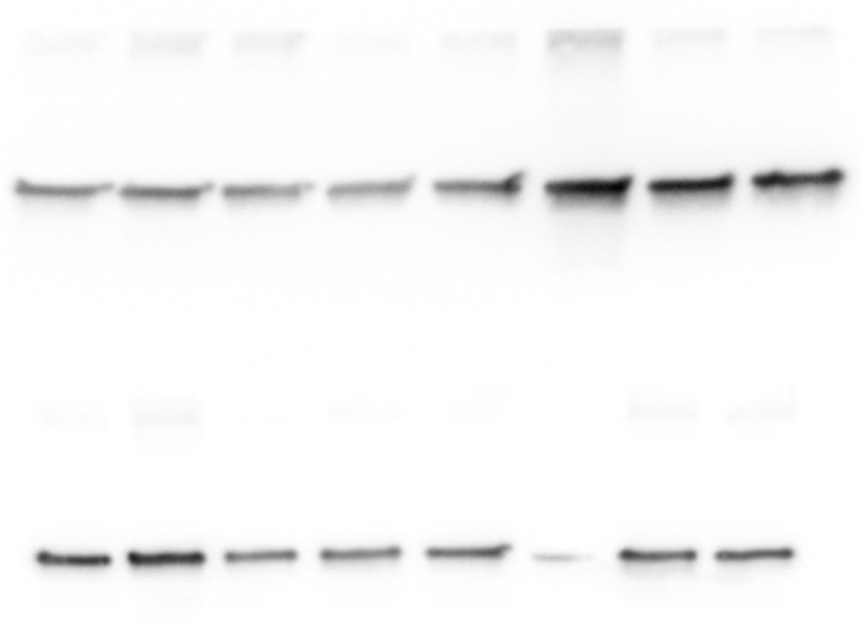

pS2814 (*Cacna1c*$^{+/-}$ samples. From right to left: 4 control condition samples and 4 isoprenaline treated samples from 8 different animals on each blot, two blots. Membranes were cut before antibody application. The upper band was analyzed. The upper membrane contains samples #1-4 for each condition, the lower membrane samples #5-8 for each condition. The lower membrane (cropped) is presented in Figure 6A.)

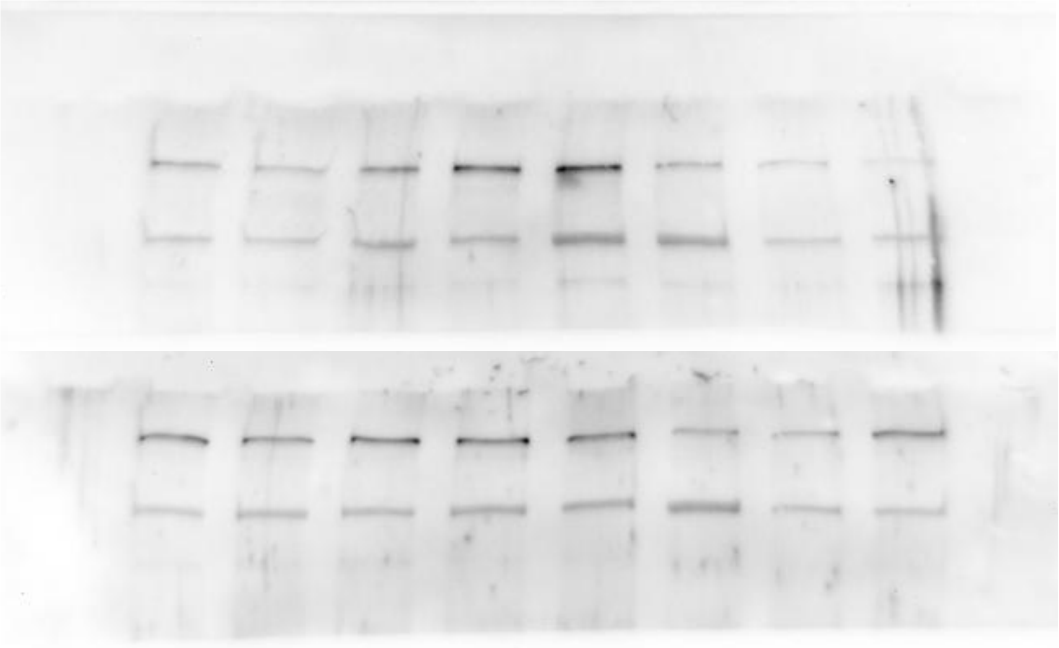

Associated GAPDH membranes (The upper membrane is the same GAPDH membrane as for RyR2, because is this case stripping was performed. The upper membrane contains samples #1-4 for each condition, the lower membrane samples #5-8 for each condition. The lower membrane (cropped) is presented in Figure 6A.)

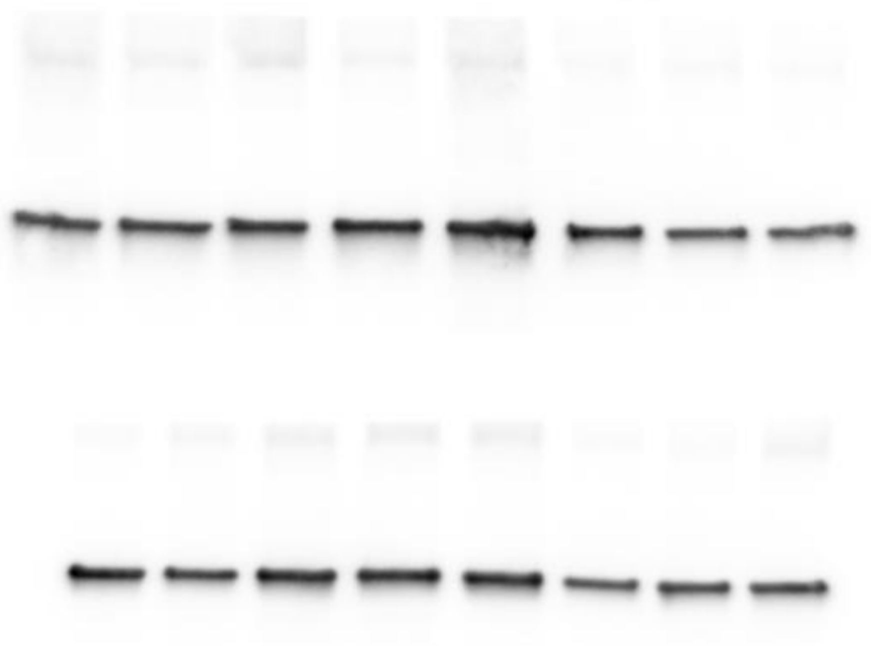

pS2808 (Wildtype samples. From right to left: 4 control condition samples and 4 isoprenaline treated samples from 8 different animals on each blot, two blots. Membranes were cut before antibody application. The upper band was analyzed. The upper membrane contains samples #1-4 for each condition, the lower membrane samples #5-8 for each condition. The lower membrane (cropped) is presented in Figure 6A.)

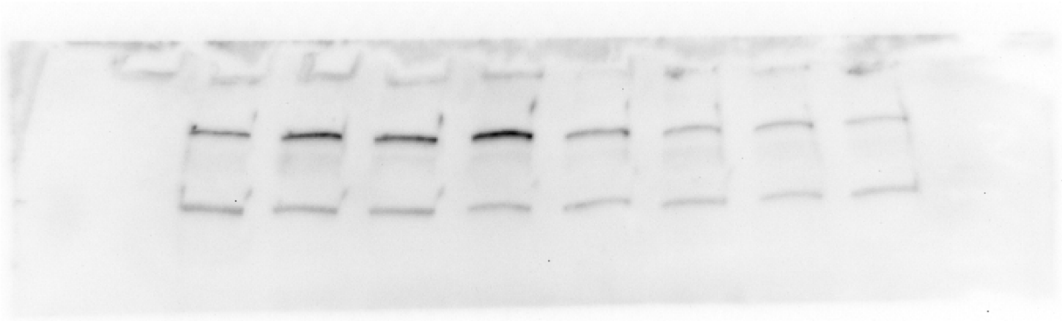

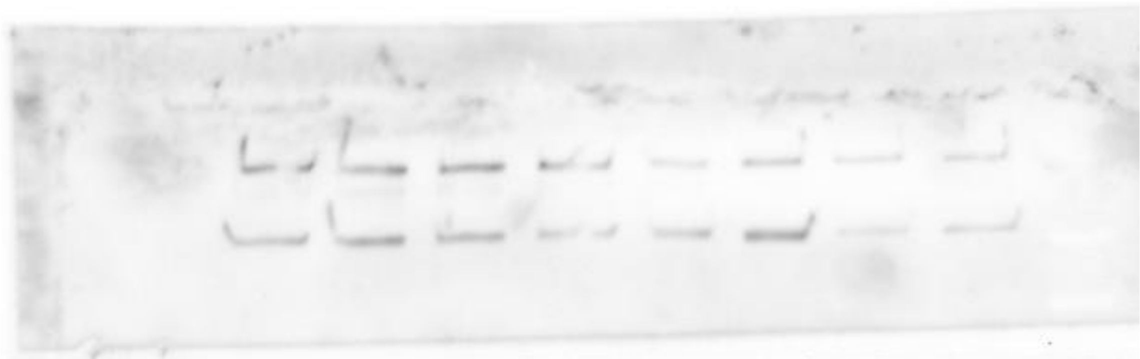

Associated GAPDH membranes: (These are the same GAPDH membranes as for RyR2, because in this case stripping was performed only for the lower membrane.) The upper membrane contains samples #1-4 for each condition, the lower membrane samples #5-8 for each condition. The lower membrane (cropped) is presented in Figure 6A.)

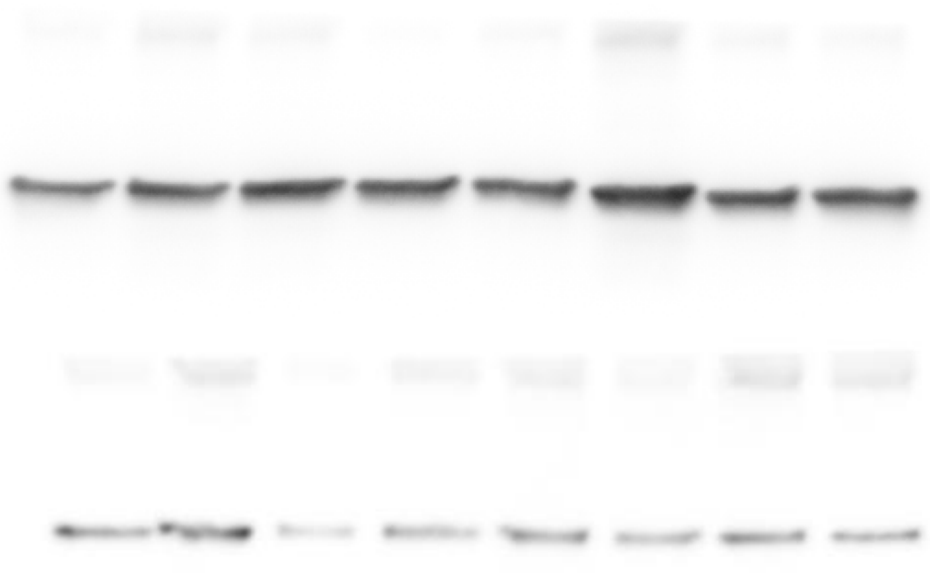

pS2808 (*Cacna1c*$^{+/-}$ samples. From right to left: 4 control condition samples and 4 isoprenaline treated samples from 8 different animals on each blot, two blots. Membranes were cut before antibody application. The upper band was analyzed. The sample far left on the second membrane is a ventricular pool sample. The upper membrane contains samples #1-4 for each condition, the lower membrane samples #5-8 for each condition. The lower membrane (cropped) is presented in Figure 6A.)

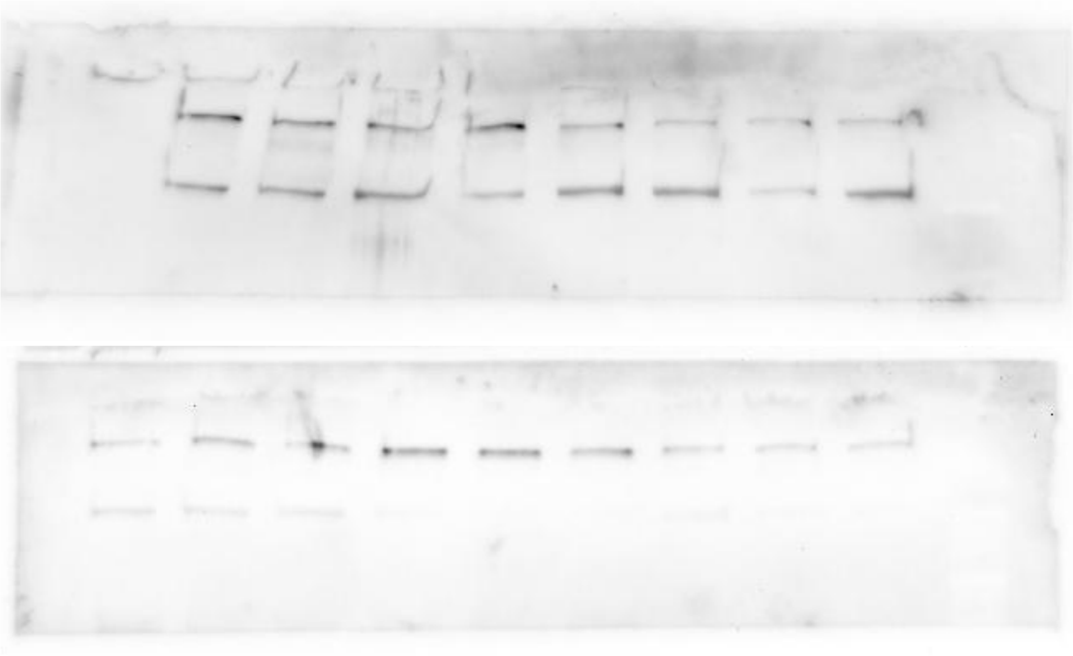

Associated GAPDH membranes:
(The lower membrane is the same GAPDH membrane as for RyR2, because in this case stripping was performed. The sample far left on the lower membrane is a ventricular pool sample. The upper membrane contains samples #1-4 for each condition, the lower membrane samples #5-8 for each condition. The lower membrane (cropped) is presented in Figure 6A.)

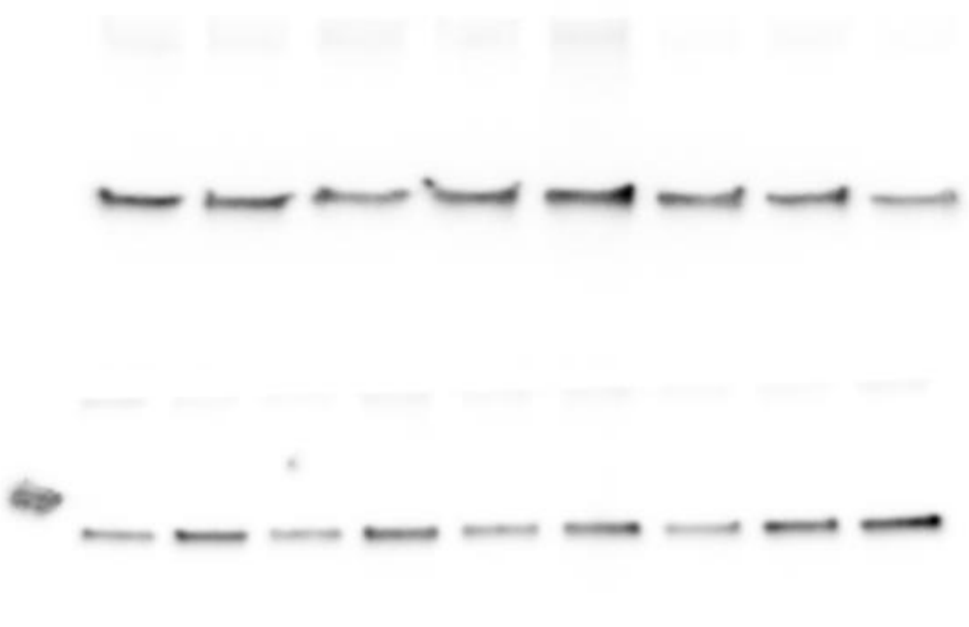

PLB (Wildtype samples. From right to left: 4 control condition samples and 4 isoprenaline treated samples from 8 different animals on each blot, two blots. Membranes were cut before antibody application. The upper membrane contains samples #5-8 for each condition, the lower membrane samples #1-4. The upper membrane (cropped) is presented in Supp. Figure S4.)

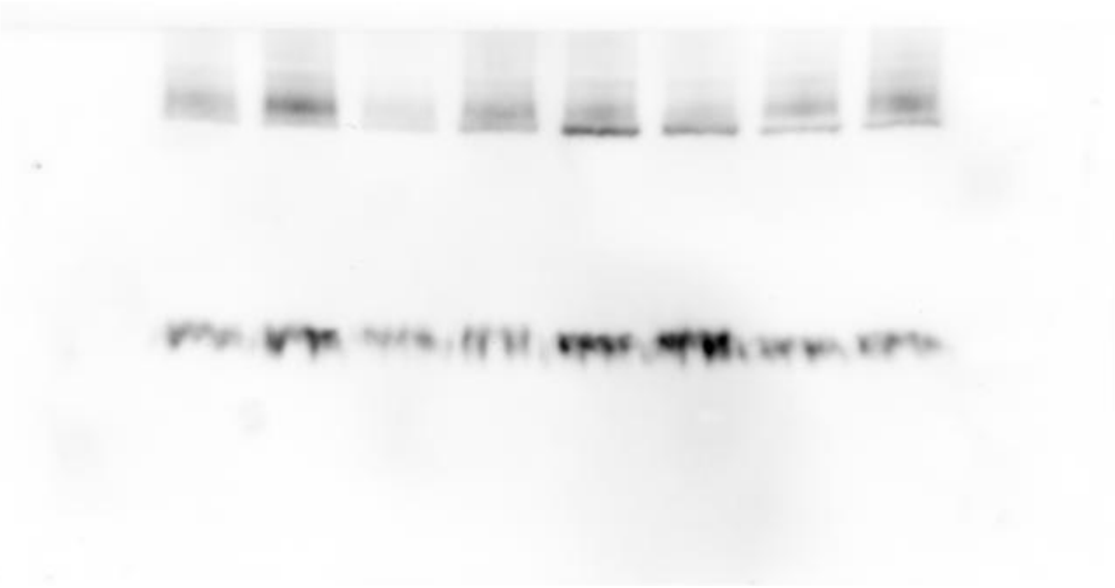

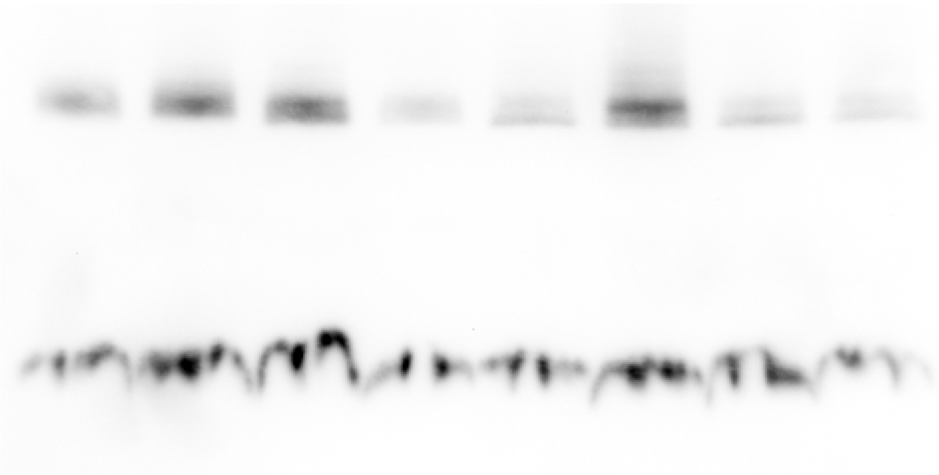

Associated CSQ membranes (The upper membrane contains samples #5-8 for each condition, the lower membrane samples #1-4 for each condition. The upper membrane (cropped) is presented in Supp. Figure S4.)

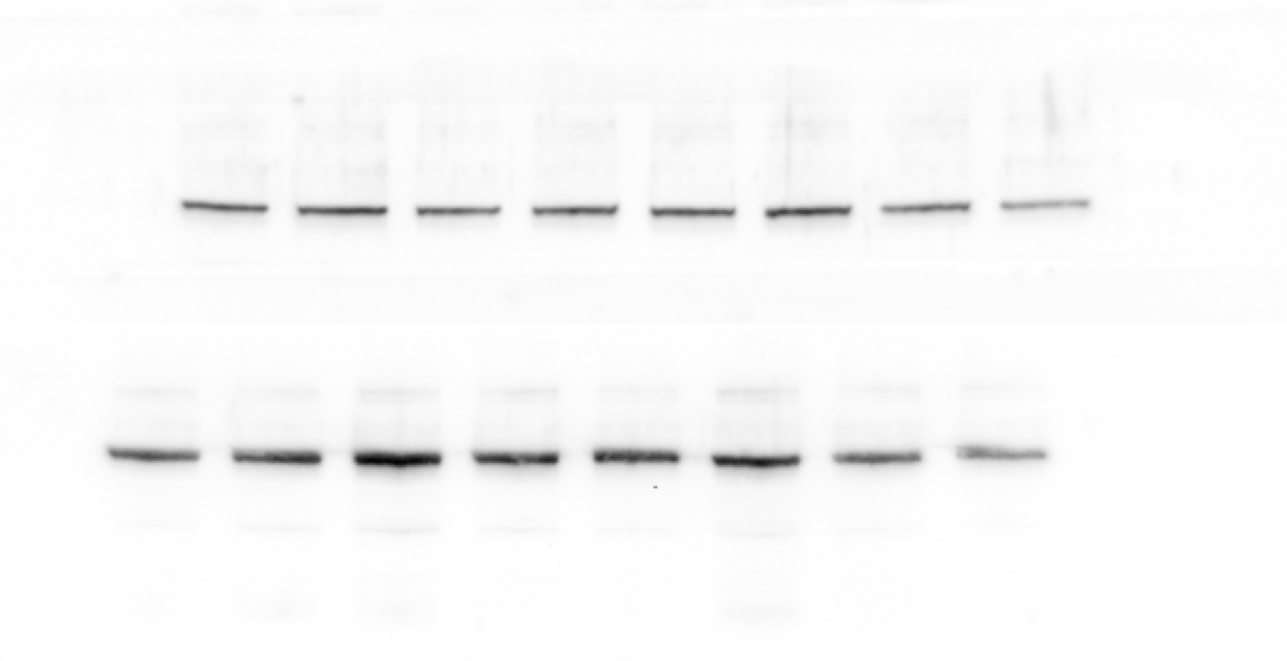

PLB (*Cacna1c*$^{+/-}$ samples. From right to left: 4 control condition samples and 4 isoprenaline treated samples from 8 different animals on each blot, two blots. Membranes were cut before antibody application. The upper membrane contains samples #5-8 for each condition, the lower membrane samples #1-4 for each condition. The upper membrane (cropped) is presented in Supp. Figure S4.)

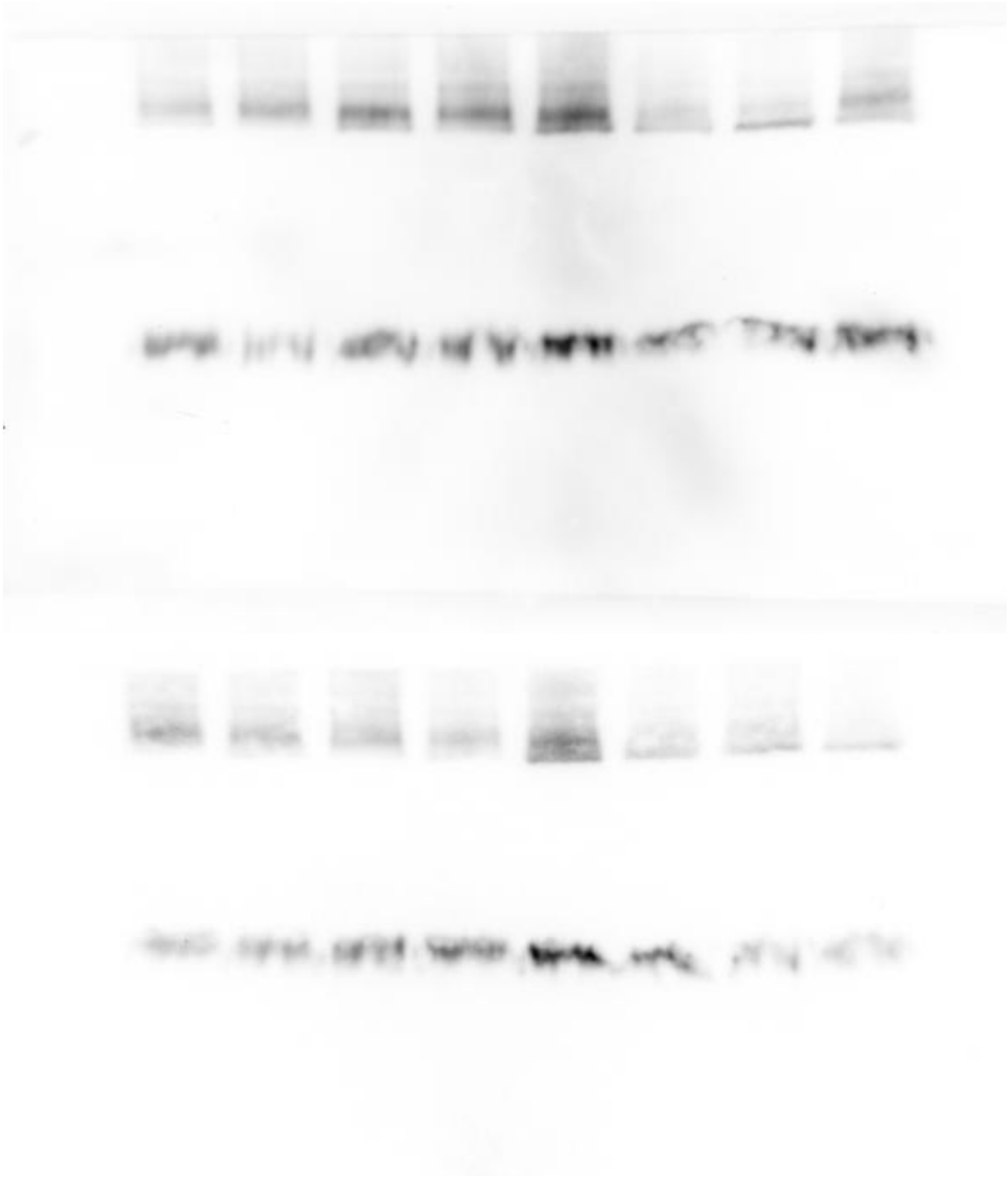

Associated CSQ membranes (The upper membrane contains samples #5-8 for each condition, the lower membrane samples #1-4 for each condition. The upper membrane (cropped) is presented in Supp. Figure S4.)

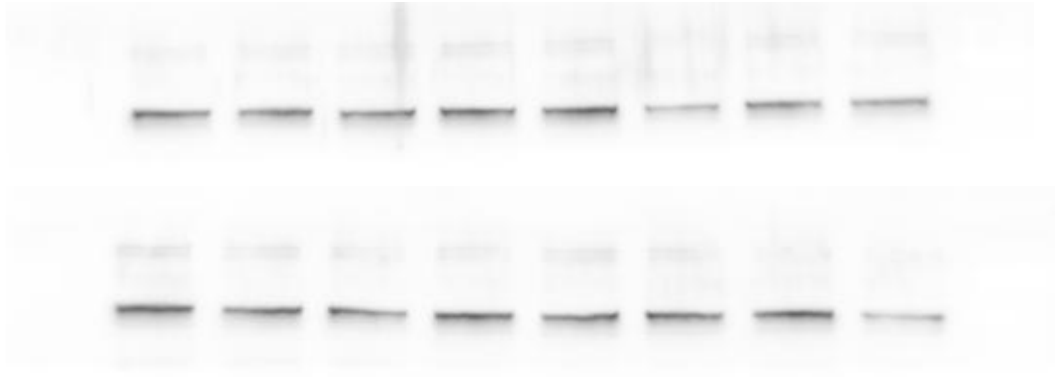

pS16 (Wildtype samples. From right to left: 4 control condition samples and 4 isoprenaline treated samples from 8 different animals on each blot, two blots. Lane far right is protein ladder. Membranes were cut before antibody application. The upper membrane contains samples #5-8 for each condition, the lower membrane samples #1-4 for each condition. The upper membrane (cropped) is presented in Supp. Figure S4.)

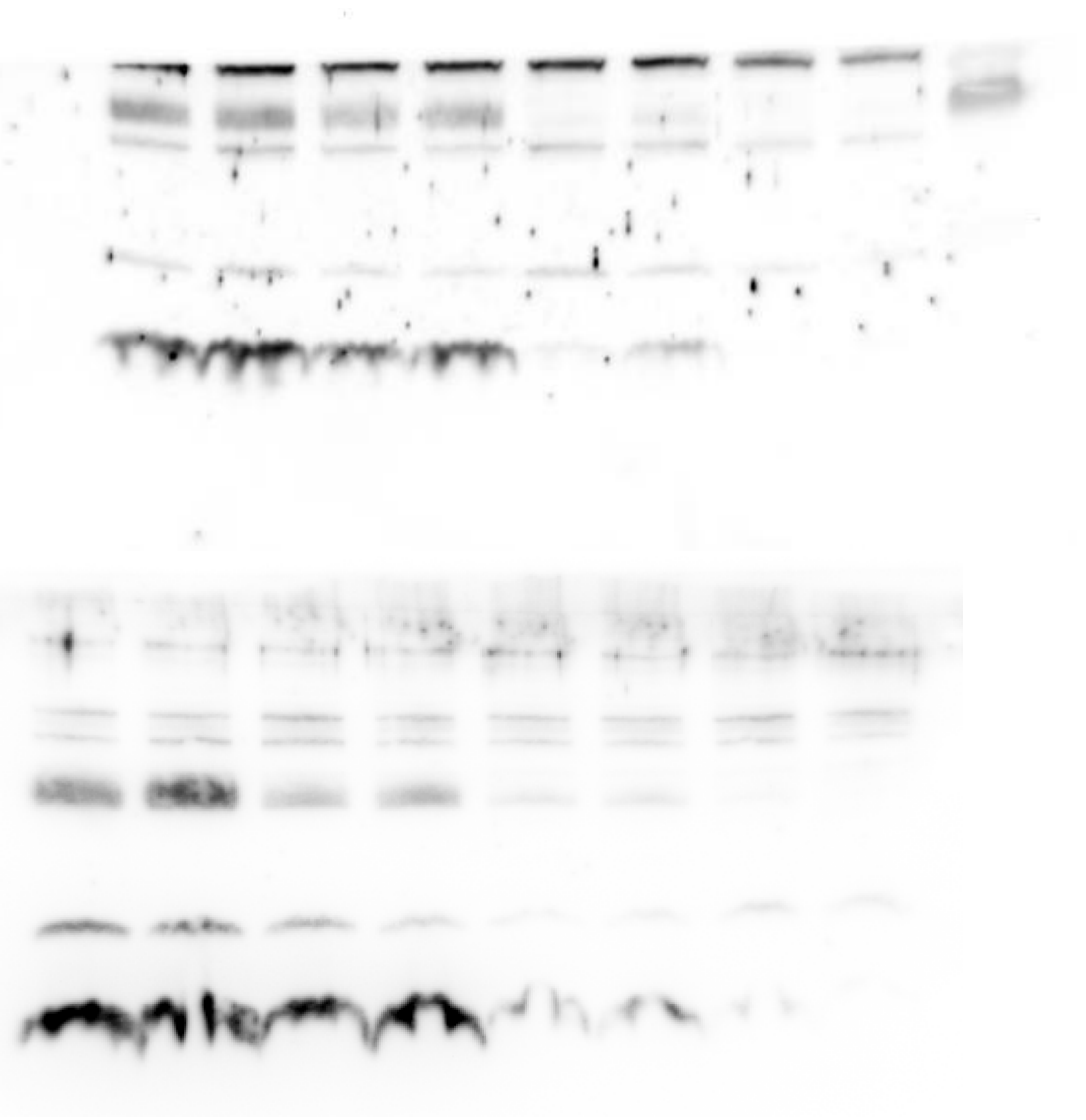

Associated CSQ membranes (The upper membrane contains samples #5-8 for each condition, the lower membrane samples #1-4 for each condition. The upper membrane (cropped) is presented in Supp. Figure S4.)

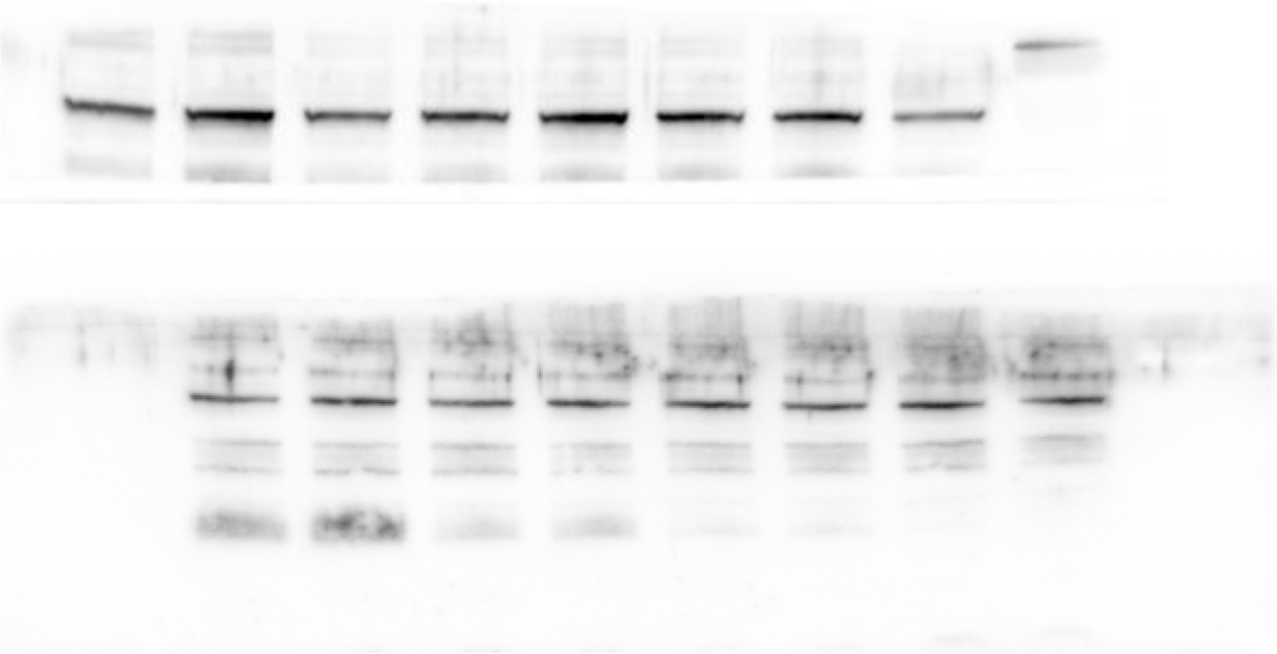

pS16 (*Cacna1c*$^{+/-}$ samples. From right to left: 4 control condition samples and 4 isoprenaline treated samples from 8 different animals on each blot, two blots. Lane far right is protein ladder. Membranes were cut before antibody application. The upper membrane contains samples #5-8 for each condition, the lower membrane samples #1-4 for each condition. The upper membrane (cropped) is presented in Supp. Figure S4.)

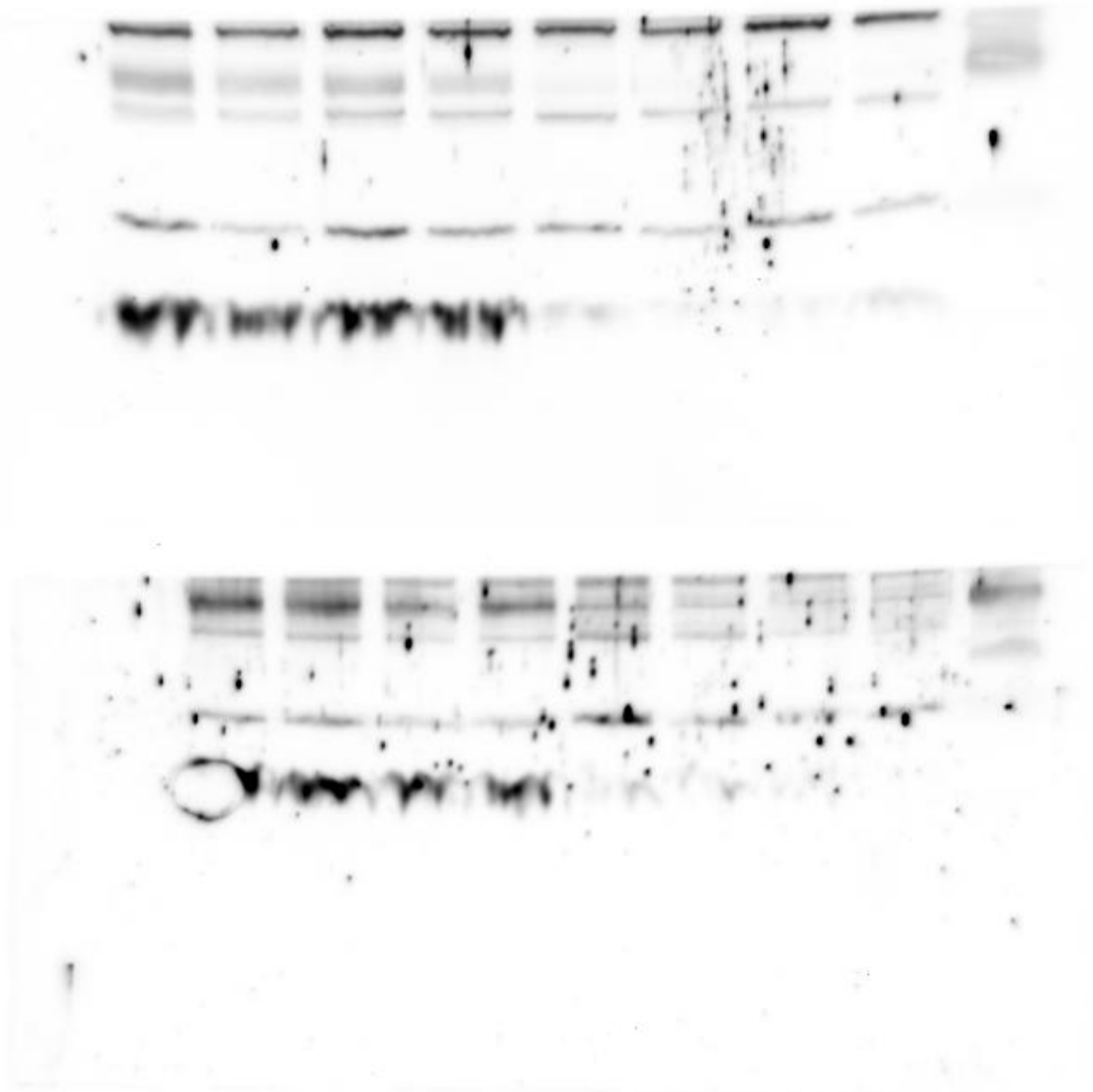

Associated CSQ membranes (The upper membrane contains samples #5-8 for each condition, the lower membrane samples #1-4 for each condition. The upper membrane (cropped) is presented in Supp. Figure S4.)

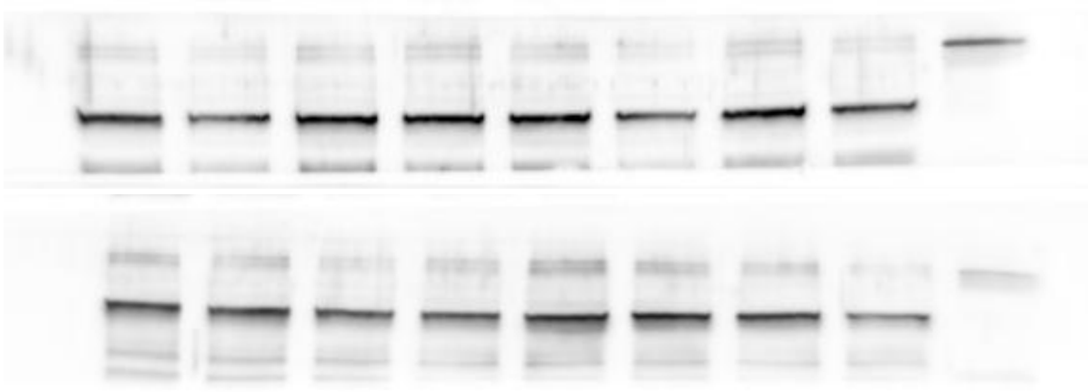

pT17 (Wildtype samples. From right to left: 4 control condition samples and 4 isoprenaline treated samples from 8 different animals on each blot, two blots. Lane far right is protein ladder. Membranes were cut before antibody application. The upper membrane contains samples #5-8 for each condition, the lower membrane samples #1-4 for each condition. The upper membrane (cropped) is presented in Supp. Figure S4.)

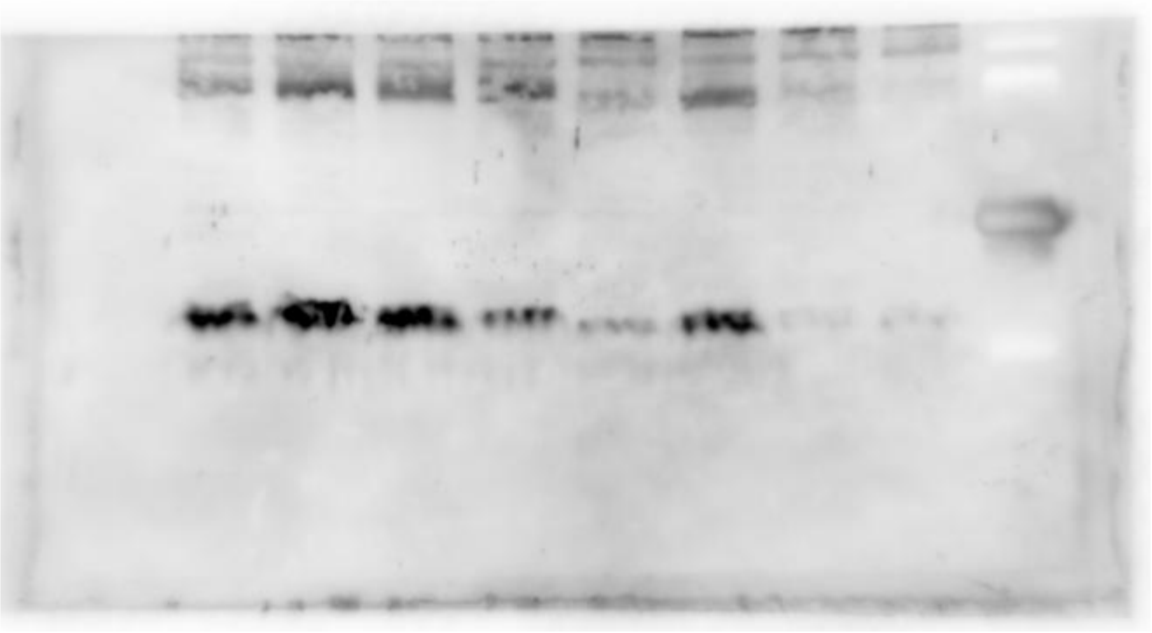

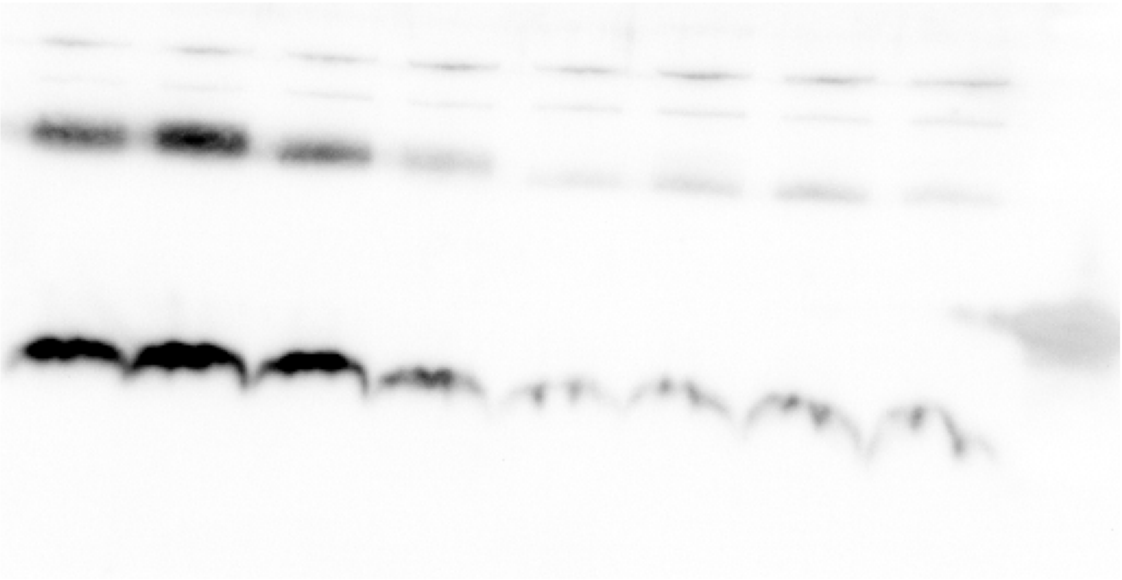

Associated CSQ membranes (The upper membrane contains samples #5-8 for each condition, the lower membrane samples #1-4 for each condition. The upper membrane (cropped) is presented in Supp. Figure S4.)

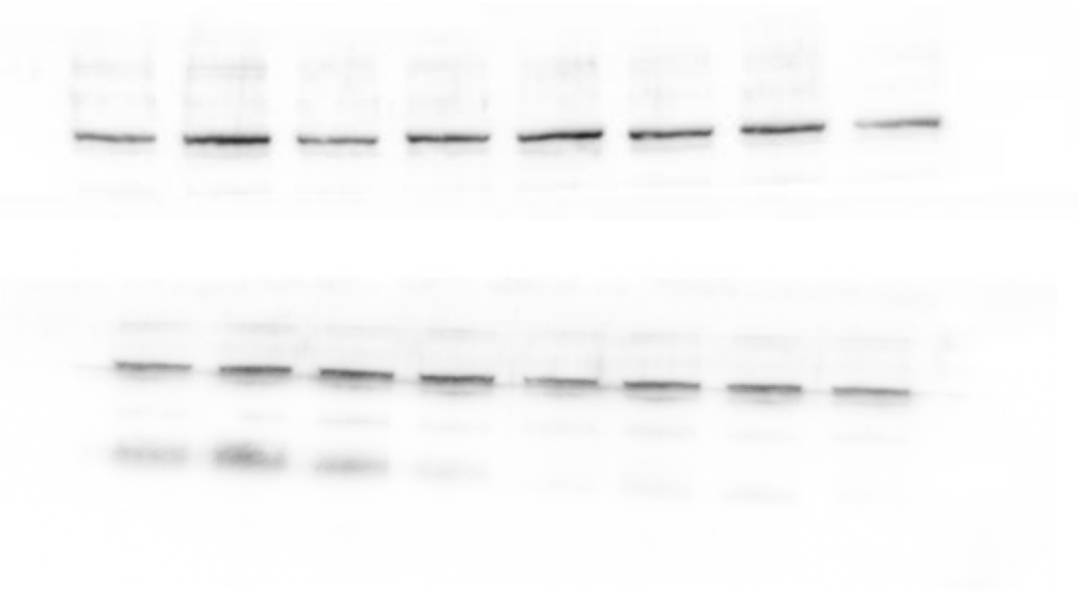

pT17 (*Cacna1c*$^{+/-}$ samples. From right to left: 4 control condition samples and 4 isoprenaline treated samples from 8 different animals on each blot, two blots. Lane far right is protein ladder. Membranes were cut before antibody application. The upper membrane contains samples #5-8 for each condition, the lower membrane samples #1-4 for each condition. The upper membrane (cropped) is presented in Supp. Figure S4.)

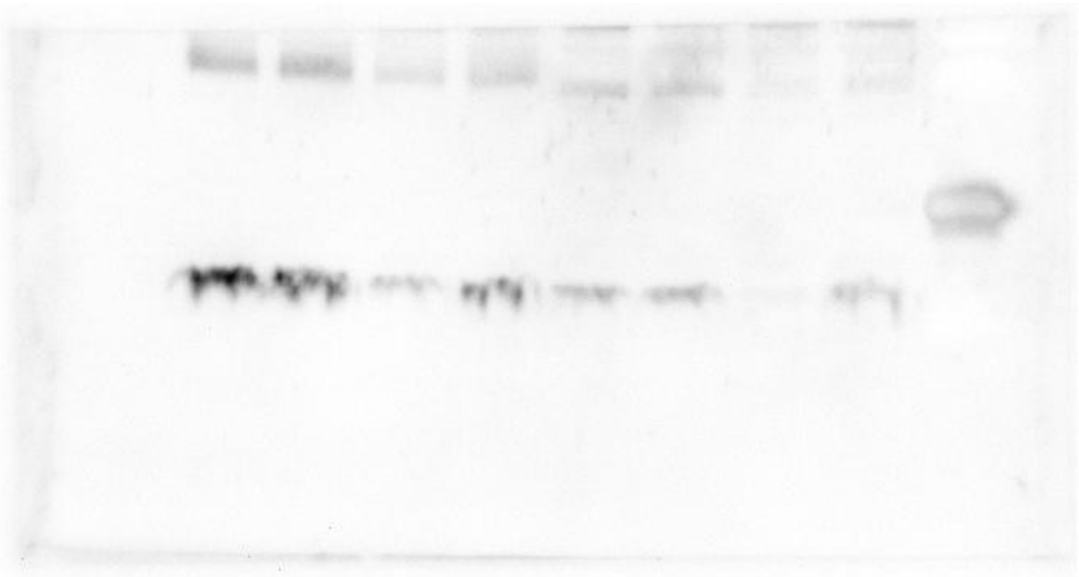

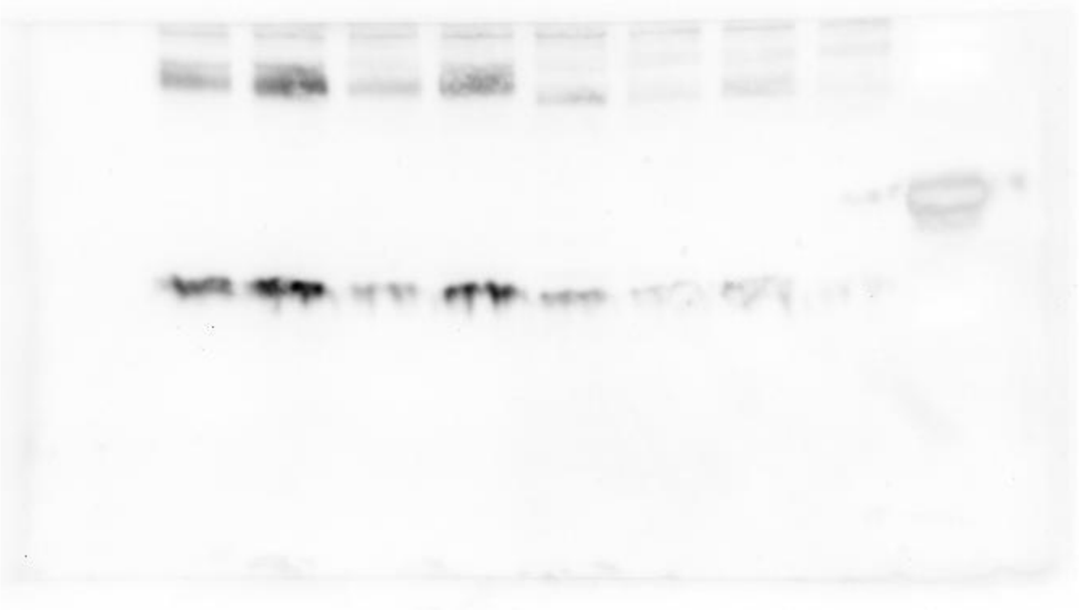

Associated CSQ membranes (The upper membrane contains samples #5-8 for each condition, the lower membrane samples #1-4 for each condition. The upper membrane (cropped) is presented in Supp. Figure S4.)

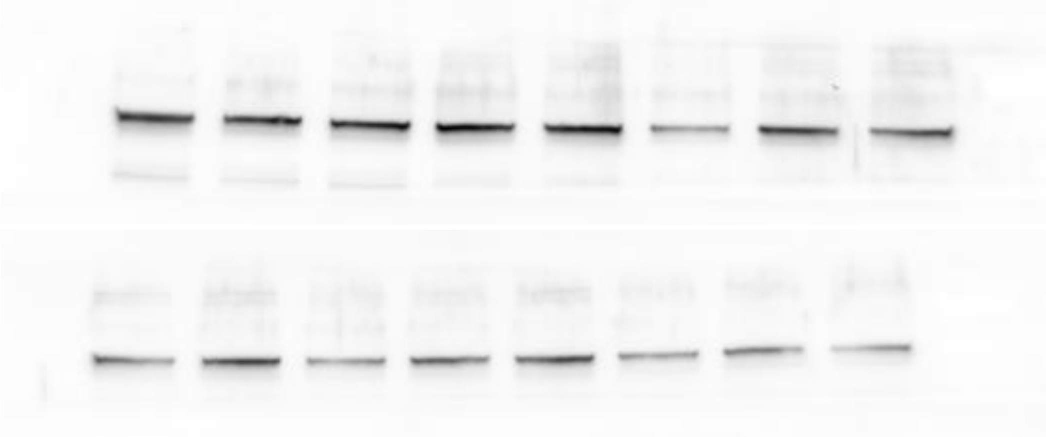

References

[1] J. Plackic, S. Preissl, Y. Nikonova, F. Pluteanu, L. Hein, J. Kockskamper, Enhanced nucleoplasmic Ca(2+) signaling in ventricular myocytes from young hypertensive rats, J Mol Cell Cardiol 101 (2016) 58-68.

[2] J. Plackic, J. Kockskamper, Isolation of Atrial and Ventricular Cardiomyocytes for In Vitro Studies, Methods Mol Biol 1816 (2018) 39-54.
